# Spatial consistency in drivers of population dynamics of a declining migratory bird

**DOI:** 10.1111/1365-2656.13834

**Published:** 2022-11-18

**Authors:** Chloé R. Nater, Malcolm D. Burgess, Peter Coffey, Bob Harris, Frank Lander, David Price, Mike Reed, Robert A. Robinson

**Affiliations:** ^1^ Norwegian Institute for Nature Research (NINA) Trondheim Norway; ^2^ Centre for Biodiversity Dynamics, Norwegian University of Science and Technology (NTNU) Trondheim Norway; ^3^ RSPB Centre for Conservation Science Sandy UK; ^4^ PiedFly.Net, Yarner Wood Devon UK; ^5^ Centre for Research in Animal Behaviour University of Exeter Exeter UK; ^6^ Merseyside Ringing Group Merseyside UK; ^7^ Forest of Dean Gloucestershire UK; ^8^ 143 Daniells Welwyn Garden City Hertfordshire UK; ^9^ British Trust for Ornithology, The Nunnery Thetford UK

**Keywords:** annual survival, comparative analysis, environmental effects, full annual cycle, integrated population model, LTRE, multi‐population, pied flycatcher

## Abstract

1. Many migratory species are in decline across their geographical ranges. Single‐population studies can provide important insights into drivers at a local scale, but effective conservation requires multi‐population perspectives. This is challenging because relevant data are often hard to consolidate, and state‐of‐the‐art analytical tools are typically tailored to specific datasets.
2. We capitalized on a recent data harmonization initiative (SPI‐Birds) and linked it to a generalized modelling framework to identify the demographic and environmental drivers of large‐scale population decline in migratory pied flycatchers (*Ficedula hypoleuca*) breeding across Britain.
3. We implemented a generalized integrated population model (IPM) to estimate age‐specific vital rates, including their dependency on environmental conditions, and total and breeding population size of pied flycatchers using long‐term (34–64 years) monitoring data from seven locations representative of the British breeding range. We then quantified the relative contributions of different vital rates and population structure to changes in short‐ and long‐term population growth rate using transient life table response experiments (LTREs).
4. Substantial covariation in population sizes across breeding locations suggested that change was the result of large‐scale drivers. This was supported by LTRE analyses, which attributed past changes in short‐term population growth rates and long‐term population trends primarily to variation in annual survival and dispersal dynamics, which largely act during migration and/or nonbreeding season. Contributions of variation in local reproductive parameters were small in comparison, despite sensitivity to local temperature and rainfall within the breeding period.
5. We show that both short‐ and long‐term population changes of British breeding pied flycatchers are likely linked to factors acting during migration and in nonbreeding areas, where future research should be prioritized. We illustrate the potential of multi‐population analyses for informing management at (inter)national scales and highlight the importance of data standardization, generalized and accessible analytical tools, and reproducible workflows to achieve them.

## Graphical abstract

Tackling large‐scale population declines requires multi‐population studies. Capitalizing on the SPI‐birds data standardization initiative, this study reveals that population changes of migratory flycatchers across its UK breeding distribution are driven by survival and dispersal. It also provides an integrated modelling workflow for analyses at even larger scales.

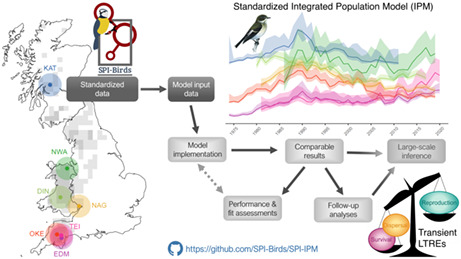

## 1 INTRODUCTION

Globally, many migratory species have been in decline over recent decades due to climate and land‐use changes (Kubelka et al., 2022). Nonetheless, implementing conservation actions that are effective at relevant regional, national, and international scales remains challenging because drivers of population decline can vary across space and time (Morrison et al., 2022) and large‐scale and long‐term multi‐population studies necessary to inform decision‐making are often lacking (but see e.g. Fisher et al., 2018). Major hurdles to implementing such studies include limited sharing of relevant data from across species ranges and challenges with harmonizing data collected and curated by different people and institutions (Culina et al., 2021).

The ongoing shift towards more open, transparent and inclusive science (Culina et al., 2018) and recent advances in data standardization have led to a number of accessible ecological databases (e.g. GBIF, eBird, trait databases). Combined with the flourishing of new statistical methods (e.g. Isaac et al., 2020), this has enabled impactful large‐scale studies in many key areas of ecological research including species distributions (Isaac et al., 2020), phenology (Bailey et al., 2022), extinction risk (Carlson et al., 2017), and abundance trends (Pagel et al., 2014). In the field of demography and population dynamics, however, this development has been lagging due to persistent concerns about data sharing and lack of resources for standardization (Culina et al., 2021; but note initiatives for processed data, such as the COMADRE database, Salguero‐Gómez et al., 2016). As a result, raw data necessary for modelling demographic processes under different environmental conditions and informing management are typically not available beyond single populations and sites.

For migratory species, this constraint is particularly acute as overlap and mixing of different populations in breeding or nonbreeding areas necessitates conservation effort at large spatial scales (Webster & Marra, 2005) at the same time as requiring detailed demographic modelling of the entire annual cycle for solid inference (Hostetler et al., 2015; Kubelka et al., 2022). Integrated population models (IPMs, Plard et al., 2019; Schaub & Kéry, 2021) have become key tools for studying links between environment, demographic rates, and population dynamics of migratory birds (e.g. Rushing et al., 2017; Woodworth et al., 2017). Through joint analysis of multiple types of individual‐ and population‐level data, IPMs provide in‐depth insights into demographic processes, even when data are scarce, and frequently increase precision of estimates (Schaub & Kéry, 2021). However, while the flexibility of Bayesian modelling frameworks allows tailoring IPMs to any combination of available data from any given study population, there have been few efforts to generalize such models to allow consistent applications across multiple populations.

In this study we develop and document a generalized IPM that is harmonized with the SPI‐Birds Network and Database (www.spibirds.org, Culina et al., 2021) and which can be readily fit to demographic data (mark–recapture and nest survey data) from many species and populations available through the database. We then use the model to identify environmental and demographic drivers of the large‐scale decline of a migratory passerine. Like many other Afro‐Palaearctic migrants, European pied flycatchers (*Ficedula hypoleuca*, hereafter ‘flycatchers’) have decreased substantially over recent decades: 29% since 1980 across Europe (PECBMS, 2020) and 43% since 1995 in the United Kingdom (Woodward et al., 2020). Despite local declines being linked to climatic factors and weather effects in both breeding and nonbreeding areas (e.g. Goodenough et al., 2009; Selonen et al., 2021), the drivers of large‐scale decline remain elusive due to a focus on single breeding populations or relatively small regions. Consequently, crucial questions such as to what degree breeding versus nonbreeding conditions influence population dynamics remain unanswered, hampering the ability to implement effective conservation measures at the most appropriate spatial scales. We address this question by fitting our IPM for seven flycatcher populations representative of the British breeding range, then use transient life table response experiments (Koons et al., 2016; Koons et al., 2017) to assess the relative importance of breeding season drivers (via reproduction) and nonbreeding season drivers (via survival and immigration) for both short‐term fluctuations in population growth rates and long‐term population trends.

## 2 MATERIALS AND METHODS

### 2.1 Study species

**FIGURE 1:**
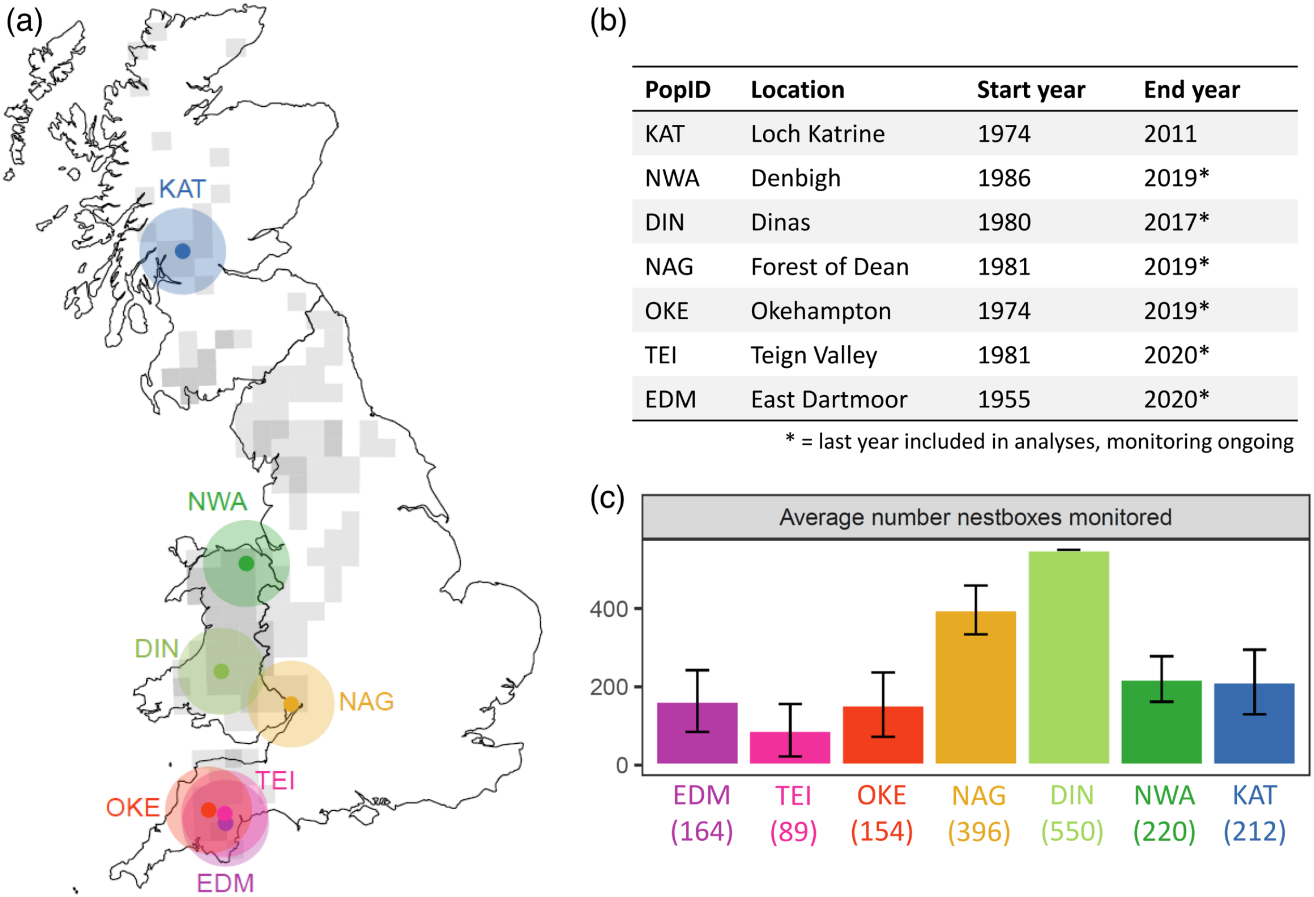
(a) Geographical location of flycatcher study populations (coloured dots) relative to the British breeding distribution (grey, with darker shading indicating higher relative abundance, data from EBBA, Keller et al., 2020). (b) Overview of location names and sampling years. (c) Mean number of nestboxes monitored per year in each study site (black bars indicate mean ± SD).

Pied flycatchers are short‐lived (<9 years) woodland songbirds that migrate annually between boreal/temperate breeding grounds in Europe and nonbreeding areas in western Africa. In Britain, the species breeds primarily in oak‐ (*Quercus* spp.) dominated woodlands across western England, Wales and Scotland (Figure 1). Flycatchers are hole‐nesting and readily take to human‐provided nestboxes. Within a breeding season, British flycatchers typically lay a single clutch of six to seven eggs (Lundberg & Alatalo, 1992). The incubation period is 13–15 days, and young typically fledge 16–17 days after hatching. Among flycatcher populations breeding in Britain, first breeding is often delayed until 2 years old, but both breeding and nonbreeding birds migrate to the breeding areas each spring (Both et al., 2017; Harvey et al., 1985). Despite a high degree of philopatry, some individuals disperse, with breeding dispersal up to 8 km, and natal dispersal as far as 660 km but more typically <3 km (Both et al., 2012; Lundberg & Alatalo, 1992).

### 2.2 Study areas and durations

We collected individual‐ and population‐level data from seven populations of flycatchers (encompassing on average 89–550 nestboxes monitored for 34–64 years) representative of the British breeding distribution (Figure 1). Most of the breeding populations initially established after the first nestboxes had been provided (e.g. Campbell, 1965) and nestboxes were provided in excess of all hole‐nesting bird species in all subsequent years (e.g. excess calculated to average 40%–44% in populations TEI, EDM and OKE).

### 2.3 Data collection and preparation

Individual‐ and population‐level data on flycatchers were largely collected by volunteers, many organized through PiedFly.Net and holding individual ringing permits granted by the British Trust for Ornithology (no additional ethical approval was required). Datasets were obtained in a harmonized standard format via the SPI‐Birds database (www.spibirds.org, Culina et al., 2021) and reformatted for analysis in R (v4.0.3, R Core Team, 2020) as detailed in the following sections (code available via GitHub, Nater, 2022).

#### 2.3.1 Breeding survey data

Flycatchers display a strong preference for breeding in nestboxes as opposed to natural cavities (Slagsvold, 1987), which allows for comprehensive monitoring (nearly all nesting attempts recorded) when nestboxes are provided in excess. For all study locations, nestboxes were surveyed at least weekly throughout the breeding season (April–July). For each nest, we recorded the total number of incubated eggs and fledglings (= number of young alive at the last survey prefledging) and, where necessary, estimated first egg laying date through back‐calculations from observations of incomplete clutches (assuming one egg is laid daily). Additionally, we identified the social parents of each nest from uniquely numbered leg rings whenever possible (see Section 2.3.2).

For our IPM analysis, we extracted five structurally different types of data from the entire breeding survey data of each population. At the population level, these were the annual number of females laying clutches (approximated as the number of first clutches laid, *n* = 18,893), and the annual sums of eggs and fledglings produced by all surveyed nests. At the individual level, we extracted data on clutch sizes (*n* = 6670) and fledgling numbers (*n* = 4836) observed in nests laid by females of known age in each year.

#### 2.3.2 Mark–recapture data

Most (>95%) nestlings hatched in nestboxes in our study sites were marked with a uniquely numbered leg ring when 8–12 days old. Breeding adults were captured in nestboxes using one‐way traps inside entrances and assumed to be immigrants into the focal breeding population if they were not yet ringed at first capture. Immigrant status was also assigned to the small subset (*n* = 148) of birds ringed as nestlings or adults in any of the six other study populations before capture in the focal population.

We re‐arranged the mark–recapture data for each population into two different types of input data for the IPM. First, we formatted the individual‐level data for all birds marked as nestlings (*n* = 83,263) and adult females (*n* = 6123) into unique capture histories. Initial captures and recaptures of adult males were omitted since the present analyses focused on females. Second, we extracted the annual number of newly immigrated breeding females, approximated as the annual number of females newly ringed as adults.

#### 2.3.3 Environmental data

Environmental factors are known to affect reproductive output of flycatchers, resulting in lower reproduction and recruitment in years with more rainfall and lower temperatures during the nestling stage (Siikamaki, 1996; Veistola et al., 1997). Accounting for effects of rainfall and temperature should therefore improve estimation of vital rates. To capture the relevant time periods for environmental covariates affecting nestlings we estimated relevant time windows for each year and population (to account for spatial and temporal variation in breeding phenology) as 8 days either side of the 0.25 quartile mean annual hatch date. Hatch date was typically not observed directly but approximated as the observed date of first egg + number of days spent laying + incubation period (14 days).

Little is known about environmental or other impacts on flycatchers after fledging and before southward migration, although it is likely that weather—and rainfall in particular—affects juvenile survival during this period (Cox et al., 2014; Naef‐Daenzer & Grüebler, 2016). We thus defined a second set of time windows for environmental impacts corresponding to this postfledging period as 7 days from the assumed date of fledging (= hatch date + 16‐day nestling period + 1).For three of our seven study populations, information on date of first egg was sparse or missing so we used the estimated windows for the closest population in which windows could be estimated. Year‐specific time windows estimated for EDM were thus used for the TEI and OKE populations (distance 11 and 27 km respectively), and windows for NWA were used for the DIN population (distance 195 km).

We downloaded data on daily interpolated minimum temperature and total precipitation for 5 × 5 km squares encompassing each study location for May–August each year 1955–2019 (2020 was not yet available) from CEDA (Met Office, 2019) using the R packages raster and ncdf4 (Hijmans et al., 2015; Pierce, 2019). We then averaged the daily temperature and rainfall values over each population‐ and year‐specific time interval and used the resulting aggregated values as environmental covariates. All covariates were z‐standardized prior to analysis.

### 2.4 IPM construction and implementation

We developed a workflow for fitting a generalized IPM (‘SPI‐IPM’) to any dataset on hole‐nesting birds contained in the SPI‐Birds database. It is immediately applicable to any species with a life‐history similar to that of flycatchers, and straightforward to extend to others (e.g. multiple clutches per year). Data formatting, model specification and model implementation are documented in detail in a code manual that accompanies the code on the SPI‐IPM GitHub repository. We therefore keep the following description of model specification and implementation to a minimum, and refer the reader to Chapters 2–4 in the online code manual for more details (static version: supplementary file ‘SPI‐IPM_CodeManual_Ch2‐4.pdf’). All parameters in the model are defined in Table S1.1.

#### 2.4.1 Age‐structured population model and data likelihoods

We describe population dynamics using a female‐based age‐structured population model with a prebreeding census. Females were divided into two age classes: ‘yearling’ (1‐year‐old birds hatched in the preceding breeding season) and ‘adult’ (birds older than 1 year), since reproductive output is expected to differ substantially between them (Fay et al., 2021). As accurate age information is often missing for considerable parts of monitored flycatcher populations, we did not further divide the adult age class but note that doing so (e.g. to account for senescence) constitutes a simple extension of the model presented here. The dynamics of the female segment of the population over the time interval from spring in year t to spring in year t+1 can be described as:

$$N_{\mathrm{tot},t+1}=\left[\begin{array}{c} N_{Y,t+1}\\ N_{A,t+1} \end{array}\right]=\left[\begin{array}{cc} 0.5\;F_{Y,t}\;{\mathrm{sJ}}_{t}\; & 0.5\;F_{A,t}\;{\mathrm{sJ}}_{t}\\ {\mathrm{sA}}_{t} & sA_{t} \end{array}\right]\left[\begin{array}{c} N_{Y,t}\\ N_{A,t} \end{array}\right]+\left[\begin{array}{c} {\mathrm{Imm}}_{Y,t+1}\\ {\mathrm{Imm}}_{A,t+1} \end{array}\right]$$

$N_{\mathrm{tot},t+1}$ represents the total number of yearling and adult females in the population upon arrival in the breeding areas in year t+1. We refer to $N_{\mathrm{tot}}$ as ‘total population size’ as it includes all females, irrespective of whether they breed in a nestbox or not. The number of yearling and adult females in the population in year t+1 ($N_{Y,t+1}$ and $N_{A,t+1}$ respectively) consists of local survivors and recruits from the previous breeding season, as well as immigrant yearling (${\mathrm{Imm}}_{Y}$) and adult (${\mathrm{Imm}}_{A}$) females. The age‐specific fecundity terms $F_{a,t}$ are products of breeding probability (${\mathrm{pB}}_{a,t})$, clutch size (${\mathrm{CS}}_{a,t}$), probability of nest success (${\mathrm{pNS}}_{t}$, *p*(complete clutch failure) = $1-{\mathrm{pNS}}_{t}$) and survival probabilities of every egg/nestling to fledging when there is no complete nest failure (${\mathrm{sN}}_{a,t}$, with a = age class of the mother). Fledglings and yearlings/adults can survive to the next breeding season and remain within the population with annual apparent survival probabilities ${\mathrm{sJ}}_{t}$ and ${\mathrm{sA}}_{t}$, respectively.

Data on various aspects of reproductive output (${\mathrm{CS}}_{a,t}$, ${\mathrm{pNS}}_{t}$, and ${\mathrm{sN}}_{a,t}$) were analysed within the IPM via generalized linear mixed models. Annual apparent survival rates (${\mathrm{sJ}}_{t}$ and ${\mathrm{sA}}_{t}$), as well as breeding probabilities (${\mathrm{pB}}_{a,t}$) were estimated by specifying an age‐structured Cormack–Jolly–Seber model for the mark–recapture data. Normally, breeding probabilities would be confounded with probabilities of recapture given breeding (CJS model recapture probability = ${\mathrm{pB}}_{a,t}\times p_{t}^{\text{CapB}}$, where $p_{t}^{\text{CapB}}$ is the probability of capture and identification given breeding in a nestbox). In our model we therefore made estimation of ${\mathrm{pB}}_{a,t}$ possible by approximating $p_{t}^{\text{CapB}}$ as the proportion of monitored nests in each breeding season t for which the breeding female had been identified (see chapter 2.2.4 in the code manual for more details).

#### 2.4.2 Temporal variation in vital rates

We accounted for among‐year variation in all (age‐specific) vital rates using environmental covariates and random effects assumed to be normally distributed on the link scale as described in Chapter 3 of the code manual.

We included previously established effects of posthatching temperature and rainfall on nest success and survival to fledging (Siikamaki, 1996; Veistola et al., 1997) Additionally, we modelled an effect of rainfall in the 7‐day period postfledging on juvenile annual survival as this is likely to affect fledglings. No additional environmental covariates were included for clutch size, which is known to be relatively invariable (Lundberg & Alatalo, 1992), nor for breeding probability and adult survival due to limited knowledge of relevant drivers and likely presence of complex indirect and delayed effects (e.g. Selonen et al., 2021) that are beyond the scope of this study.

#### 2.4.3 Bayesian implementation

We implemented the IPM for each study population separately in a Bayesian framework using Nimble v0.12.1 (de Valpine et al., 2017) and estimated parameters via Markov chain Monte Carlo (MCMC). We used noninformative priors with biologically sensible upper bounds for all parameters (see code manual Chapter 2.3 for details) and simulated initial values for all nodes to avoid initialization problems. Missing covariate values were imputed within the model where necessary. We ran four MCMC chains of 200,000 iterations each, of which the first 50,000 were discarded as burn‐in, and which were subsequently thinned to retain every 30th sample. Chain convergence was verified using visual inspection and the Gelman–Rubin statistic (Gelman & Rubin, 1992).

### 2.5 Model testing

Since there are no global goodness‐of‐fit tests available for IPMs (Plard et al., 2019), we used three complementary approaches to assess our IPM ability to produce biologically relevant estimates of vital rates and realistic representations of flycatcher population dynamics. We first plotted predictions of the numbers of breeders and breeding immigrants, and several measures of reproductive output, against relevant observational data to ensure that predictions were not substantially biased (Gelman et al., 2013). Second, we checked for major discrepancies among datasets and between datasets and the population model by comparing posterior distributions of vital rate parameters obtained from the IPM to those obtained from models estimating each vital rate independently (Gelman et al., 2013; Kéry & Schaub, 2012). Lastly, we verified that models could make realistic predictions of population dynamics by running stochastic forward projections based on posterior median estimates (Gabry et al., 2019). The three‐step model testing procedure, including results, is further described in Supporting Information S2.

### 2.6 Transient life table response experiments (LTRE)

Life table response experiments (LTREs) are retrospective analyses that allow quantification of the relative contributions of changes in different vital rates to changes in population growth rates (Caswell, 2000). Transient LTREs can further evaluate contributions from changes in population structure under nonstationary conditions and are particularly suited for IPMs, which provide estimates of both vital rates and population size/structure (Koons et al., 2016; Koons et al., 2017). We used different types of transient LTREs to investigate the drivers of both short‐term and long‐term changes in growth rates of all seven focal populations.

Random‐design LTREs (Caswell, 2000, chapter 10.2) quantify contributions of among‐year variation in a vital rate/population structure components $\theta_{i}$ to total annual variation in realized population growth rates, $\mathrm{var}\left(\lambda_{t}\right)$:

$$\text{Contributio}n_{\theta_{i}}^{\mathrm{var}\left(\lambda_{t}\right)}\approx {\left.\sum_{i}\mathrm{cov}\left(\theta_{i,t},\theta_{j,t}\right)\frac{\delta \lambda_{t}}{\delta \theta_{i,t}}\frac{\delta \lambda_{t}}{\delta \theta_{j,t}}\right|}_{\bar{\theta }}$$

where $\mathrm{cov}\left(\theta_{i,t},\theta_{j,t}\right)$ is the covariance of the quantity of interest ($\theta_{i}$) with all other quantities ($\theta_{i-}$), and $\frac{\delta \lambda_{t}}{\delta \theta_{i,t}}$ is the sensitivity of $\lambda_{t}$ with respect to $\theta_{i,t}$. Fixed‐design LTREs (Caswell, 2000, chapter 10.1), on the other hand, calculate the contribution of a change in the value of $\theta_{i}$ from year t to year t+1 to the change in annual growth rate over the same time interval ($\Delta \lambda_{t}$):

$$\text{Contributio}n_{\theta_{i}}^{\Delta \lambda_{t}}\approx {\left.\left(\theta_{i,t+1}-\theta_{i,t}\right)\frac{\delta \lambda_{t}}{\delta \theta_{i,t}}\right|}_{\bar{\theta }}$$

Koons et al. (2016) introduced an additional LTRE design (here referred to as the ‘period design’) which focuses on long‐term population changes by calculating contributions of changes in vital rate means ($\mu_{i}$) and standard deviations ($\sigma_{i}$) to changes in geometric mean growth rates ($\Delta \lambda_{g}$) between two time periods (P1 and P2):

$$\text{Contributio}n_{\theta_{i}}^{\text{logΔ}\lambda_{g}}\approx \left(\log\mu_{i,P2}-\log\mu_{i,P1}\right)\left({\bar{e}}_{\mu_{i}}^{T}+{\bar{e}}_{\mu_{i}}^{\hat{n}}\right)+\left(\log\sigma_{i,P2}-\log\sigma_{i,P1}\right)\left({\bar{e}}_{\sigma_{i}}^{T}+{\bar{e}}_{\sigma_{i}}^{\hat{n}}\right)$$

In addition to partitioning contributions into those due to changes in mean and standard deviation, the period‐design LTRE further distinguishes between direct effects of changes in the vital rate (expressed by real‐time elasticities ${\bar{e}}_{\mu_{i}}^{T}$ and ${\bar{e}}_{\sigma_{i}}^{T}$) and indirect changes mediated by perturbation of population structure as a consequence of vital rate changes (real‐time elasticities ${\bar{e}}_{\mu_{i}}^{\hat{n}}$ and ${\bar{e}}_{\sigma_{i}}^{\hat{n}}$). The two time periods compared need to have the same duration, and we selected two equal length periods capturing different population trajectories for all seven study populations (Figure S1.1). We derive the sensitivities for all parameters in the IPM in Supporting Information S3, additional details on the period‐design LTRE are provided in Supporting Information S4, and we refer to Koons et al. (2016, 2017) for more information.

The implementation of transient LTREs for IPMs as introduced by Koons et al. (2017) assumes closed populations. Since the flycatcher IPM includes immigration, we accounted for this in the LTRE analyses. Calculating sensitivities for immigration rates for use in random‐ and fixed‐design LTREs is straightforward (Supporting Information S3, see also Nater et al., 2021; Paquet et al., 2021). The derivation of real‐time elasticities for immigration rates (for use in the period‐design LTRE) is new, and we detail our approach in Supporting Information S4. Code for implementing and running all three types of LTRE is provided in the GitHub repository (Nater, 2022).

## 3 RESULTS

Comparison of IPM predictions to observed data suggested no major lack of fit for any population (Supporting Information S2.1). Posterior distributions from independent and integrated models largely overlapped, although the IPM tended to estimate lower adult survival and, in some cases, adult clutch size and nest success probability compared to the independent data models (Supporting Information S2.2). Stochastic projections indicated that the IPMs were able to predict realistic population dynamics (S2.3).

The results presented here are based on the posterior samples of three of four run chains for each population (the third chain was excluded from the posterior of all models since it did not converge within 200,000 iterations in the model for OKE, and convergence issues for this model persisted when using different initial values and/or MCMC seeds) and are reported as Median [95% credible interval]. Posterior summaries for vital rate parameters are also provided in Table S1.2 (separate supplementary file ‘TableS2.csv’).

### 3.1 Temporal dynamics of seven populations

**FIGURE 2:**
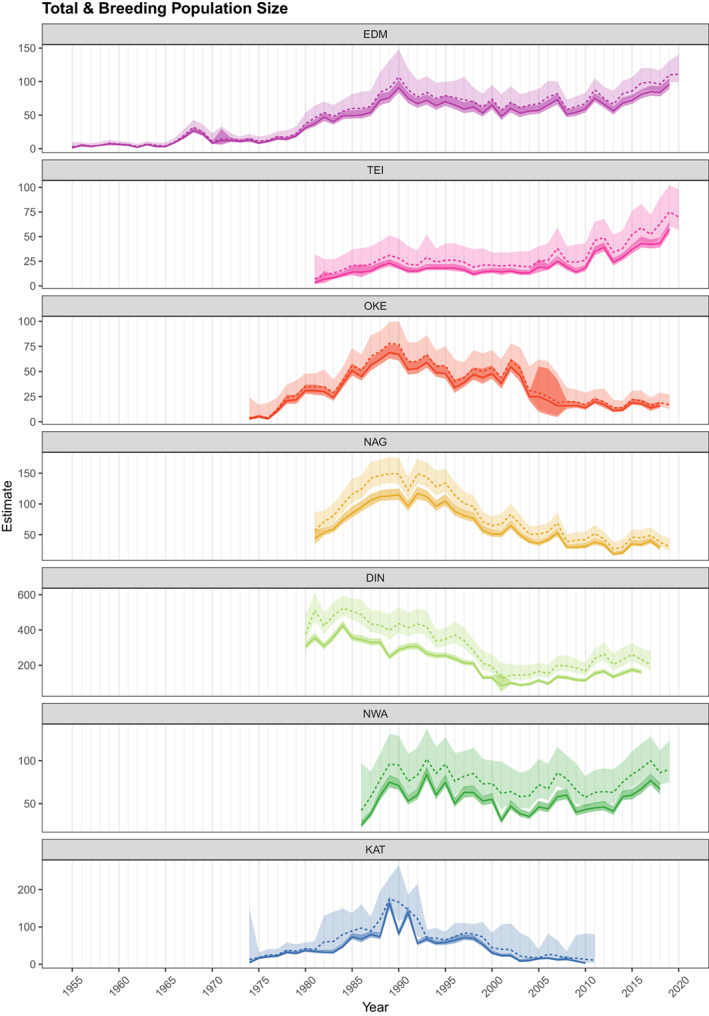
Annual estimates of the total number of females (dashed line) and the number of females breeding in nestboxes (solid line) for all seven study populations. The difference between these two estimates is that the former includes nonbreeders, temporary emigrants and birds nesting in natural cavities as opposed to nestboxes. Lines represent the posterior median estimates, ribbons mark the 95% credible intervals (pale = total number of females, more coloured = number of females breeding in nestboxes).

Across all seven populations total population sizes showed periods of increase, decrease and stability (Figure 2). Variation in breeding population size (defined as the number of females breeding in nestboxes in a given year) largely tracked the temporal pattern in total population size, with an average of between 68[65, 73]% (DIN) and 86[77, 95]% (OKE) of the total population being reproductively active. The two southernmost populations (TEI and EDM) showed overall positive trends in population sizes over their study periods (Figure 2; Table S1.3). Correlation coefficients indicated negative population trends for four populations (OKE, NAG, DIN and KAT, Table S1.3) although most of these also saw a period of population increase early in their study periods (Figure 2). A post‐hoc covariation analysis further provided evidence for substantial (primarily) positive associations of year‐by‐year changes in population size across study sites (Figure S1.2).

### 3.2 Time‐average age‐specific vital rates

**FIGURE 3:**
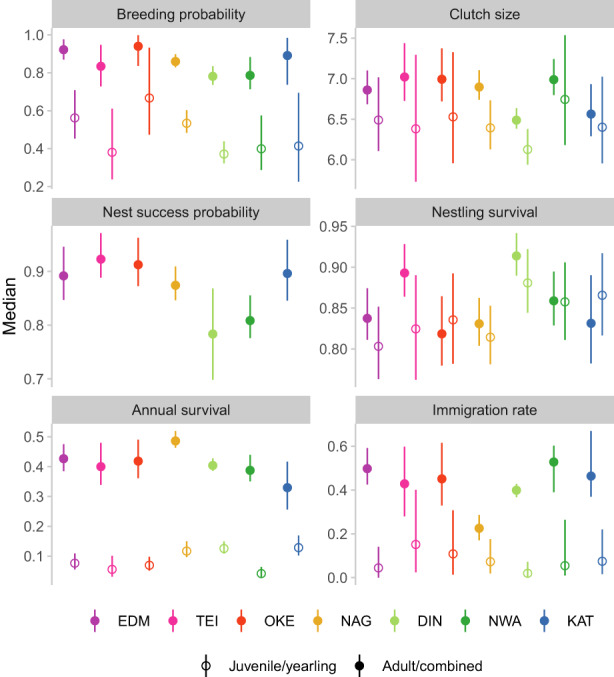
Posterior medians (dots) and 95% credible intervals (lines) for estimated time‐average vital rates for the seven study populations. Open symbols = younger age class (juveniles for annual survival, yearlings otherwise). Filled symbols = adults (combined age class for nest success probability). For numerical summaries, see Table S1.2.

Within populations, vital rates associated with both survival and reproduction were higher for older birds (Figure 3; Figure S1.3–S1.9). In all populations, most immigrants were adults, with yearling immigration rates estimated below 0.2 (Figure 3). The degree of variation in average vital rates differed depending on the vital rate: breeding and juvenile survival probabilities, for example, varied substantially across populations while clutch size and nestling survival were more similar (Figure 3; Figure S1.10). There were no strong associations between vital rate averages and study site latitude, but more northern populations (DIN, NWA and KAT) tended to have higher nestling survival and lower adult annual survival. Furthermore, the two Welsh populations (DIN and NWA) were characterized by substantially lower nest success probabilities than the other populations.

### 3.3 Among‐year variation in and environmental effects on vital rates

The degree of among‐year variation in vital rates varied both across parameters and populations (Table S1.2; Figures S1.11–S1.18). Models estimated substantial variation in juvenile and adult annual survival, nest success probability, nestling survival and adult immigration rates. Estimated breeding probabilities were relatively invariable in the more southern populations (TEI, EDM, OKE and NAG) but showed more variation in Wales and Scotland (DIN, NWA and KAT, Figure S1.13). Clutch size and yearling immigration rates were relatively constant in all populations (Figures S1.14 and S1.17). Overall, there was very little evidence for time trends in vital rates; the exceptions were an indication of decreasing breeding probability and increasing immigration rate for DIN and increasing adult survival and decreasing nestling survival for NWA (Table S1.3).

**FIGURE 4:**
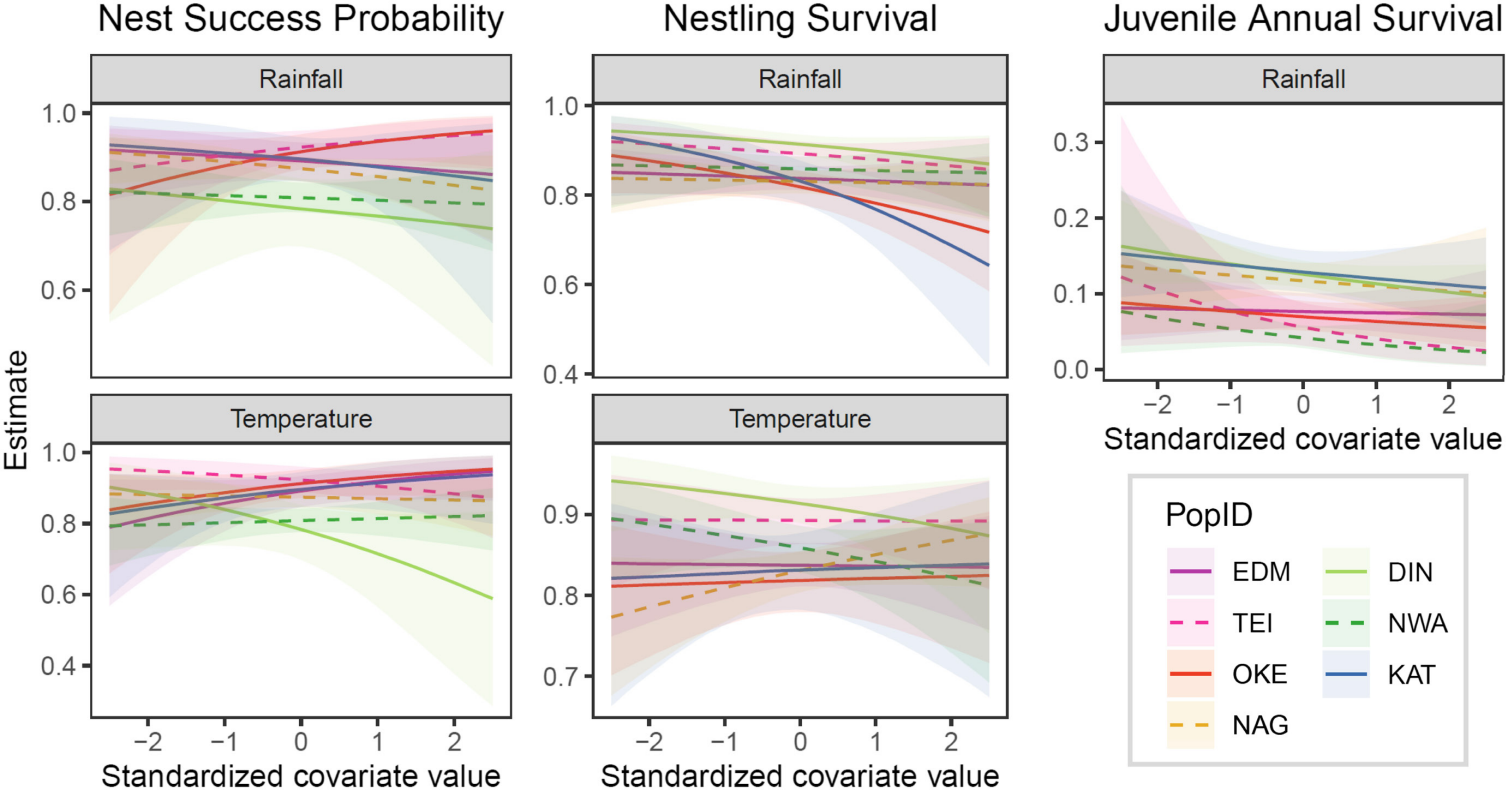
Effects of rainfall (top row) and temperature (bottom row) on nest success probability, nestling survival and juvenile annual survival (columns) of the seven study populations. For nest success probability and nestling survival, environmental covariates represented conditions during a 16‐day window posthatching. For juvenile annual survival, the rainfall covariate covered a 7‐day period postfledging. Environmental variables are plotted on a standardized scale for easier comparison across populations. An alternative representation of the relationships, including visualization of raw data, can be found in Figure S1.25.

Posterior estimates of the slope parameters for environmental effects provided mixed evidence for rainfall and temperature directly impacting nest success probabilities, nestling survival and juvenile survival (Figure 4; Table S1.4). While the 95% credible intervals of all estimated effects in all populations overlapped with 0, their posterior distributions (also summarized through additional 90% and 50% credible intervals in Table S1.4) still provided insights into potential rainfall and temperature effects. Notably, almost all estimated effects of rainfall on vital rates had negative posterior medians, and for several, a decrease in the vital rate with increasing rainfall was clearly visible (Figure 4). Temperature effects, on the other hand, did not show a general direction and were estimated anywhere between moderately positive and moderately negative (Figure 4; Table S1.4).

### 3.4 Demographic contributions to year‐by‐year variation in population growth rate

**FIGURE 5:**
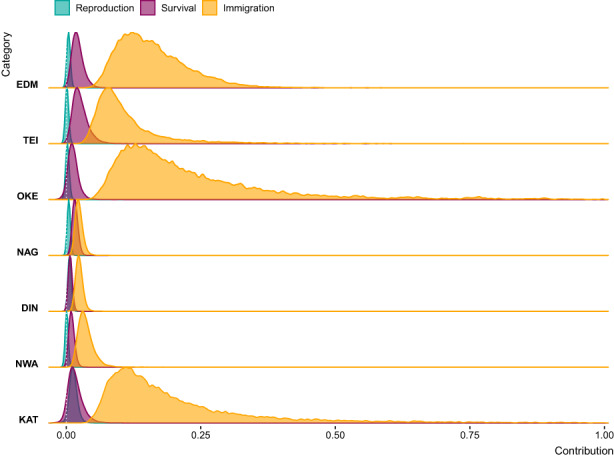
Posterior distributions of the contributions of reproduction (breeding probability, nest success probability, nestling survival probability), survival (juvenile and adult annual survival) and immigration rates to variation in realized annual population growth rates. Contributions from local population structure were negligible and are omitted here (but see Figure S1.20).

Results from the random‐design LTRE indicated that among‐year variation in annual population growth rates was driven primarily by changes in immigration rates, followed by changes in survival (Figure 5). Contributions from changes in reproductive parameters and local population structure had little influence on short‐term population growth rates in most locations (Figure 5; Figures S1.19 and S1.20). For two populations (DIN and KAT), however, variation in annual survival and reproductive output (primarily nest success and breeding probability respectively) were of similar importance. In all populations, contributions from changes in reproductive parameters of adults were larger than for yearlings, and adult immigration was consistently more influential than yearling immigration (Figure S1.20). For survival contributions, juveniles made a bigger impact than adults in four populations (TEI, EDM, NAG and DIN), similar impact in one (NWA), and smaller impact in two (OKE and KAT, Figure S1.20). Results remained consistent when the first 5 years were excluded from analyses.

**FIGURE 6:**
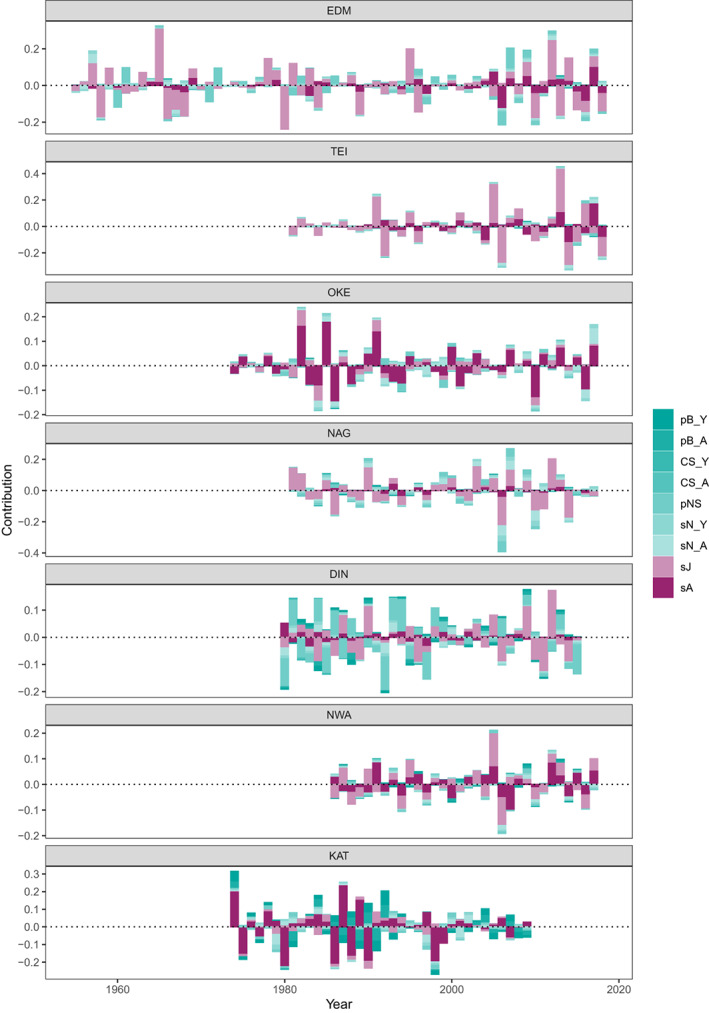
Posterior medians of stacked contributions of vital rates representing reproduction (turquoise shades) and survival (pink shades) to year‐by‐year changes in annual population growth rate over time for each study population. The sum of all contributions approximates the total rate of change in population size from 1 year to the next. Contributions from local population structure and immigration are omitted here to facilitate comparison of reproduction versus survival contributions but see Figure S1.21 for the same figure including all types of contributions.

Conclusions regarding relative importance of different demographic processes from the fixed‐design LTRE generally aligned with those obtained from the random‐design LTRE (above), but further revealed that ‘atypical’ years, that is, years in which changes in reproduction had more impact than changes in survival, occurred in all populations (Figure 6; Figure S1.21). Furthermore, population growth rate changes in some years were driven by opposing contributions from reproduction and survival (clearly visible for KAT where changes in breeding probability were often the opposite to other vital rates, Figure 6). The largest population growth rates tended to coincide with disproportionately large contributions from changes in adult immigration rates (Figure S1.21). Otherwise, patterns in relative contributions of different vital rates were not clearly related to the magnitude or direction of population change in any given year, nor were there any clear trends of long‐term changes in relative importance of different demographic components (Figure S1.22).

### 3.5 Demographic contributions to long‐term trends in population growth rate

**FIGURE 7:**
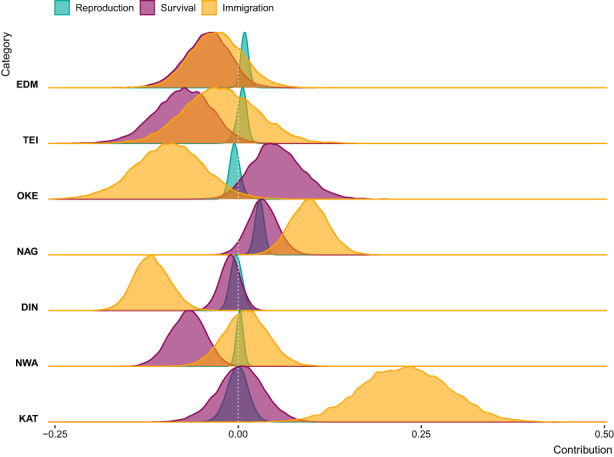
Posterior distributions of the contributions of reproduction (breeding probability, nest success probability, nestling survival probability), survival (juvenile and adult annual survival) and immigration rates to changes in long‐term population trends within the study period. The time periods compared for each population are shown in Figure S1.1. Contributions include both effects of direct changes in vital rates and indirect effects caused by perturbation of population structure due to vital rate changes.

Results from the period‐design LTREs indicated that changes in long‐term trends from one time period to the next (Figure S1.1) were driven by similar relative contributions of changes in survival and reproduction as variation in short‐term population growth rates, but that changes in immigration rates were much less influential at longer time‐scales (Figure 7). As in the random‐design LTRE, changes in reproductive and immigration rates of adults were generally more influential than changes in the equivalent rates of yearlings (Figure S1.23). Survival contributions of juveniles and adults to changes in long‐term trends, however, were more balanced in several populations (Figure S1.23). Among reproductive parameters, changes in nest success probability had the strongest effect on changes in long‐term population trends (except NAG and KAT, where nestling survival and breeding probability had stronger contributions respectively; Figure S1.23). In all populations, direct changes in vital rate means were responsible for most changes in population trajectories from one time period to the next; contributions from direct effects of changes in vital rate variation and from indirect effects (through perturbation of population structure) were negligible in comparison (Figure S1.24).

## 4 DISCUSSION

### 4.1 Population trajectories and environmental effects

Across the seven study sites, populations showed periods of increase, decrease and stability (Figure 2). We found substantial variation in both averages of, and environmental impacts on, key demographic parameters across breeding locations. For example, populations located further north tended to have higher nestling survival, which may be related to longer photoperiods providing more time for parents to forage and provision nestlings (Lundberg & Alatalo, 1992), while generally wetter conditions in Wales may contribute to lower average nest success there. The relationships between reproductive parameters and temperature varied substantially across breeding sites, suggesting localized effects. Consistent with other studies (e.g. Burgess, 2014; Siikamaki, 1996), higher rainfall had predominantly negative effects on nest success and nestling survival. Importantly, we also found clear negative effects on juvenile annual survival from higher rainfall experienced during the postfledging period before migration. This period is associated with high mortality in songbirds, yet is markedly understudied compared to other parts of the annual life cycle (Cox et al., 2014; Naef‐Daenzer & Grüebler, 2016). High or prolonged rainfall between fledging and postbreeding migration is likely to be detrimental to young that need to achieve good body condition before migration (Cox et al., 2014) and may be an overlooked, but key cause of mortality affecting population growth. This warrants more studies, especially given that temperate latitudes are predicted to experience an increased frequency of short, but intense bouts of rainfall (Westra et al., 2014).

Analysing population dynamics in multiple breeding locations simultaneously enabled us to compare trajectories across populations. Population sizes were broadly positively correlated, but covariation was not necessarily strongest for neighbouring locations (Figure S1.2). This points towards large‐scale drivers acting beyond individual breeding sites. While some drivers may act at a regional scale during the breeding period, weak connectivity (i.e. different breeding populations mixing during migration and/or in nonbreeding areas) is common among long‐distance migrants (Finch et al., 2017) and may generate population synchrony via conditions encountered on shared migration routes and/or nonbreeding areas (discussed in more detail in 4.2).

Breeding population size followed changes in nestbox provision only in some locations, which, along with sometimes large estimated fractions of nonbreeders (Figure 2), indicates that high densities of nestboxes alone may not be sufficient to increase breeding populations. The presence of nonbreeding individuals is common for many bird species, including flycatchers across their breeding range (Both et al., 2017; Newton, 1998). Loman (2006) showed experimentally that targeted provision of nest sites may only be an effective measure for increasing breeding density in high‐quality habitat/territory, and so simply increasing nestbox density without other considerations is not necessarily the most effective approach for increasing reproductive output and population growth.

### 4.2 Population dynamics driven by survival and dispersal

For all study populations, LTRE results consistently showed that both short‐term fluctuations and long‐term trends in population size were primarily driven by changes in survival and immigration (Figures 5, 6, 7; Figures S1.19–S1.23). In comparison, changes in reproduction played a small role, consistent with the cross‐population covariation we found indicating large‐scale drivers of population dynamics likely acting outside the breeding season (Section 4.1). Taken together, these results provide compelling evidence that the key drivers of flycatcher population dynamics primarily operate outside breeding areas, and that this applies across the British breeding distribution. Mallord et al. (2016) arrived at a similar conclusion for flycatchers, and three other Afro‐Palaearctic migratory bird species breeding across the United Kingdom, and further found that structural changes in breeding habitat could not explain population declines.

Another mechanism often invoked as a cause of declines is a breeding season trophic mismatch between the peak food requirements of nestlings and the peak availability of seasonal invertebrate prey (see Both et al., 2006). Our results show relatively small contributions of reproduction to population growth rates, and an absence of time trends in reproductive output (Table S1.3), suggesting that trophic mismatch is unlikely to explain declines of British breeding flycatchers. This is not surprising, given little recent or historic phenological matching of the British flycatcher nestling period with peak caterpillar abundance in oak woodlands (Burgess et al., 2018).

In only two populations (DIN and KAT) did reproduction contribute similarly to both short‐ and long‐term population dynamics as survival (Figures 5 and 7; Figure S1.19). Notably, these two populations not only declined markedly, but also represent the two sites with the lowest nestbox density (per area). They may therefore be small relative to the environment's carrying capacity, a state that Sæther et al. (2016) found to lead to relatively larger contributions of reproduction in birds generally. For all other populations survival contributions outweighed reproduction substantially (Figures 5 and 7; Figure S1.19), suggesting that the drivers of variation in annual survival rates are also important drivers of population dynamics. These drivers are difficult to study in long‐distance migrants such as flycatchers, as they may act during migration, at stop‐over sites, and in the nonbreeding areas. A growing body of evidence suggests that nonbreeding conditions can impact population growth rate through survival across seasons in migratory animals, including birds using both the American (Rushing et al., 2017; Saracco & Rubenstein, 2020; Woodworth et al., 2017) and Afro‐Palaearctic flyway (Howard et al., 2020; Selonen et al., 2021).Where studies have been able to examine different nonbreeding stages separately (e.g. Rushing et al., 2017; Woodworth et al., 2017), lower survival during spring migration is frequently found responsible for reductions to population growth rate. This illustrates that further studies of conditions, resource requirements, and fitness constraints during the different stages of the nonbreeding period, and how effects can cascade to breeding stages, are required to identify the mechanisms underlying changes in annual survival of pied flycatchers and migratory animals more generally.

### 4.3 Immigration: Crucial and cryptic

Our LTRE analyses revealed that immigration rates were more crucial than survival for local population dynamics, at least with regards to fluctuations in annual population growth rates (Figure 5). While this is commonly found for birds (Millon et al., 2019) transient LTREs frequently overestimate contributions of immigration to population dynamics when immigration rates are estimated as latent parameters within IPMs (Paquet et al., 2021). The IPMs in our analysis, however, estimate immigration based on observed counts of newly marked individuals, which limits the amount of unexplained variation that can be absorbed into immigration rates and hence results in more robust LTRE estimates. Contributions of immigration to population dynamics still need to be interpreted carefully as immigration rates are inherently scale dependent (Reichert et al., 2021; Schaub et al., 2013). First, when nestlings are marked in nestboxes only, any hatched in natural cavities will be considered immigrants, even if the natural cavities are within or very close to a study site (Millon et al., 2019). This is likely rare in our seven study populations as natural cavities were relatively scarce (Burgess, 2014), and flycatchers have a strong preference for breeding in nestboxes (Slagsvold, 1987), which were provided in excess. Second, the smaller the spatial scale at which immigration contributions are considered, the larger these are likely to be (e.g. Schaub et al., 2013). This can, for example, explain the relatively higher and more influential immigration rates in the three relatively smaller study sites in Devon (TEI, EDM, and OKE, Figures 1 and 5). Third, our results also highlight that assessments of the importance of immigration need to consider temporal scales in addition to spatial scales, as changing immigration rates were less important for long‐term population trends than for short‐term fluctuations (Figures 5 and 7). All caveats considered, our study still provides evidence for an important role of dispersal for flycatcher population dynamics across British breeding sites and highlights a need for studying the drivers of dispersal and immigration.

### 4.4 Moving forward: IPMs For comparative and range‐wide studies

While the present study focused on British breeding sites, declines of flycatcher populations are pan‐European (PECBMS, 2020), and the British breeding range is but a small component (Keller et al., 2020). SPI‐Birds currently hosts data from over 30 breeding populations across Europe, and our standardized modelling framework and analysis workflow are designed for the straightforward inclusion of additional populations. Importantly, this can grant studies more inferential power by considering both temporal and spatial replicates (effective space‐for‐time substitution)—as demonstrated in this study—and opens new possibilities for data integration beyond the population level. Datasets from many sites can be linked in multi‐population models to improve conservation outcomes through estimating and matching actions to large‐scale spatio‐temporal variation in demography and population dynamics (Morrison et al., 2022) and enable demographic studies at range‐wide scales through shared hyperparameters (e.g. Horswill et al., 2019). Similarly, the generalized IPM could be extended into an integrated meta‐population model by formally linking datasets from different sites through movement parameters (McCrea et al., 2010; Paquet et al., 2020). This is particularly relevant for identifying drivers and consequences of dispersal dynamics. In practice, estimating movement parameters for a meta‐population model could benefit from extending data sources beyond SPI‐Birds and integrating with other large‐scale databases such as the EURING bird ringing database (Du Feu et al., 2016). Standardized integrated population models combining data from both SPI‐Birds and EURING will enable formal estimation of dispersal dynamics, overcoming a key challenge with comparative demographic studies: making rates of survival, emigration, and immigration comparable across populations by disentangling them from each other and from sizes and features of local study areas (e.g. Kendall et al., 2006). Finally, our framework can readily be extended into a multi‐species IPM (Quéroué et al., 2021), for example, to model the dynamics of entire guilds of interacting hole‐nesting bird species and so potentially improve guidance for conservation management (e.g. Engelhardt et al., 2020). Given the large potential for future extensions of our generalized IPM into multi‐population, meta‐population, and multi‐species frameworks, as well as its potential applicability to other species groups (e.g. temperate‐nesting waterfowl), we have strived to increase accessibility and re‐usability of not just the model but the entire workflow through publishing our analysis toolbox including detailed, dynamic and user‐friendly documentation.

## 5 CONCLUSIONS

We show that both annual variation in population growth rate and long‐term population trends of pied flycatchers across the British breeding range are driven by survival and dispersal dynamics. Even though reproduction was sensitive to temperature and rainfall, our results reinforce the need to identify and quantify the largely unknown factors generating variation in survival and immigration rates. Future study and conservation efforts need to focus on migratory routes and nonbreeding areas and consider connectivity among different breeding populations. The latter can be greatly facilitated by the link between our IPM and the SPI‐Birds database, and the resulting ease of including additional study sites. Our well‐documented and generalized modelling framework can also serve as a starting point for a multitude of large‐scale comparative and range‐wide population analyses of both single and multiple bird species.

## AUTHOR CONTRIBUTIONS

Chloé R. Nater, Malcolm D. Burgess and Robert A. Robinson conceived and designed the methodology; Malcolm D. Burgess, Peter Coffey, Bob Harris, Frank Lander, David Price and Mike Reed collected substantial parts of the data; Chloé R. Nater analysed the data; Chloé R. Nater and Malcolm D. Burgess led the writing of the manuscript and share first authorship. All authors contributed critically to the drafts and gave final approval for publication.

## CONFLICT OF INTEREST

The authors have no conflict of interest.

## Supporting information


Appendix S1

Table S2

Table S3

Table S4

## Data Availability

The processed data and MCMC samples necessary for reproducing results and graphs presented in this study are available in the Dryad Digital Repository https://doi.org/10.5061/dryad.rbnzs7hf9 (Nater et al., 2022). Original data can be requested from SPI‐Birds through their website https://spibirds.org/. Code for formatting data, implementing and running models and analyses, and plotting results is available on GitHub: https://github.com/SPI‐Birds/SPI‐IPM. The version of code used for this study is archived on Zenodo https://doi.org/10.5281/zenodo.7253297 (Nater, 2022). An up‐to‐date version of the code manual is published here: https://spi‐birds.github.io/SPI‐IPM/.
