# Supplementary material for: Spatial consistency in drivers of population dynamics of a declining migratory bird
Source: J Anim Ecol. 2022 Nov 18;92(1):97–111. doi: 10.1111/1365-2656.13834 (PMC10099983; doi:10.1111/1365-2656.13834)
Supplement: Supplementary file 1 — Appendix S1 [file JANE-92-97-s002.zip › JANE_13834_FlycatcherIPM_Supporting_Information.pdf]

Supporting Information S1–S4

This file contains the SI for:
'Spatial consistency in drivers of population dynamics of a declining migratory bird'

Chloé R. Nater*[1,2], Malcolm D. Burgess[3,4,5], Bob Harris[6], Peter Coffey[6], Frank Lander[4,7], David Price[4], Mike Reed[8] and Rob Robinson[9]

[1]Norwegian Institute for Nature Research (NINA), Trondheim, Norway
[2]Centre for Biodiversity Dynamics, Norwegian University of Science and Technology, Trondheim, Norway
[3]RSPB Centre for Conservation Science, Sandy, UK
[4]PiedFly.Net, Yarner Wood, Bovey Tracey, Devon, UK
[5]Centre for Research in Animal Behaviour, University of Exeter, Exeter, UK
[6]Merseyside Ringing Group, UK
[7]Forest of Dean, Lydney, Gloucestershire, UK
[8]143 Daniells Welwyn Garden City, Hertfordshire, AL7 1QP, UK
[9]British Trust for Ornithology, The Nunnery, Thetford, Norfolk, UK

Contents

S1 Supplementary Figures and Tables **2**
S1.1 Figures . . . 2
S1.2 Tables . . . 27

S2 Model Assessment **29**
S2.1 Comparing predictions to data . . . 29
S2.2 Comparing estimates from integrated vs. independent analyses . . . 37
S2.3 Projecting stochastic population dynamics . . . 45

S3 Sensitivity derivation for random- and fixed-design LTRE **48**
S3.1 Sensitivities with regards to vital rates . . . 49
S3.2 Sensitivities with regards to population structure . . . 49

S4 Period-design LTRE with immigration **50**
S4.1 Calculating real-time elasticities . . . 50
S4.2 Extension to open populations . . . 52
S4.3 Dealing with zeros in vital rate estimates . . . 55

*chloe.nater@nina.no

S1 Supplementary Figures and Tables

S1.1 Figures

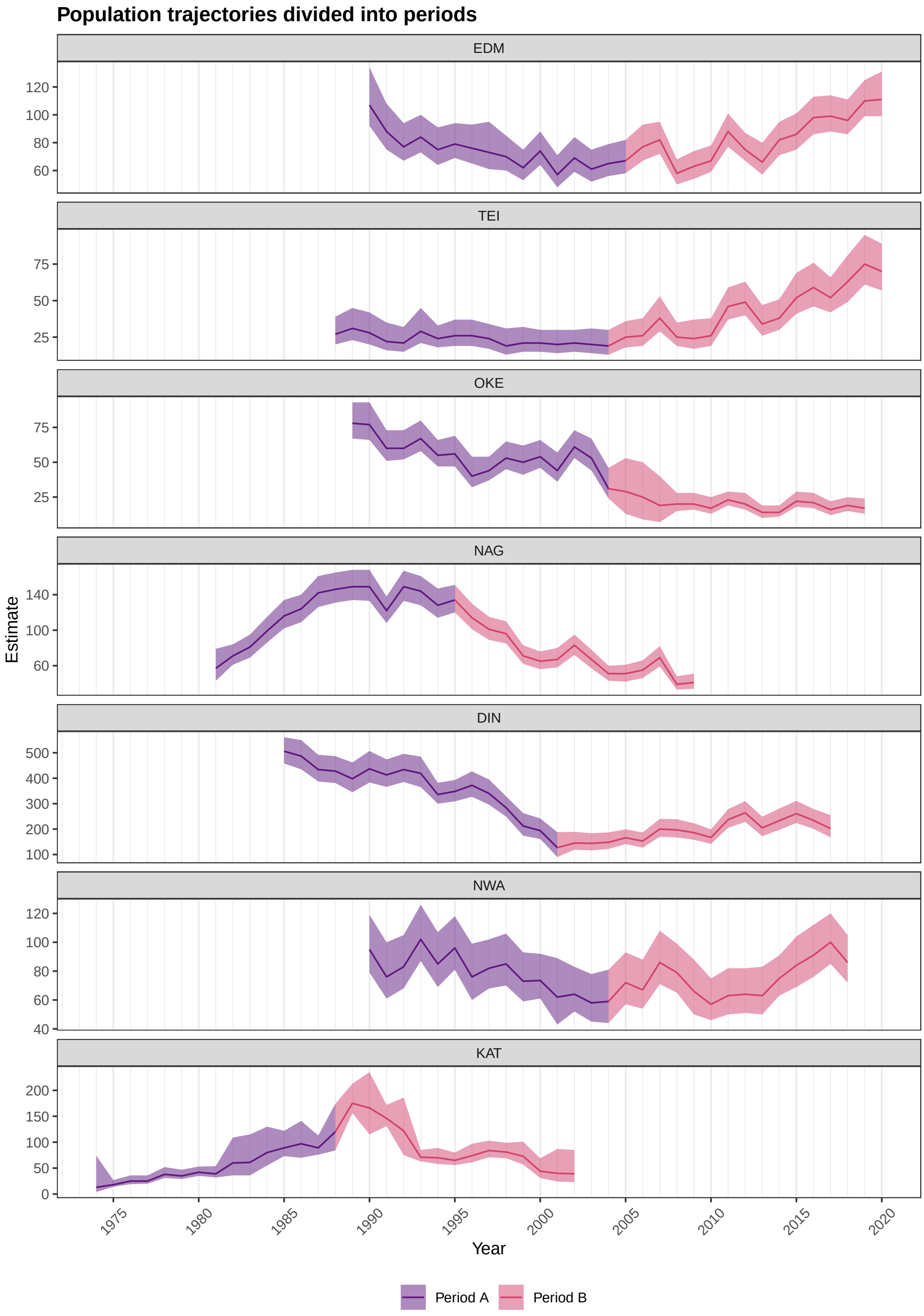

Figure S1.1: Estimated population trajectories for all populations, with time-periods compared within the period-design LTRE highlighted. Lines represent the posterior median estimates, ribbons mark the 95% credible intervals.

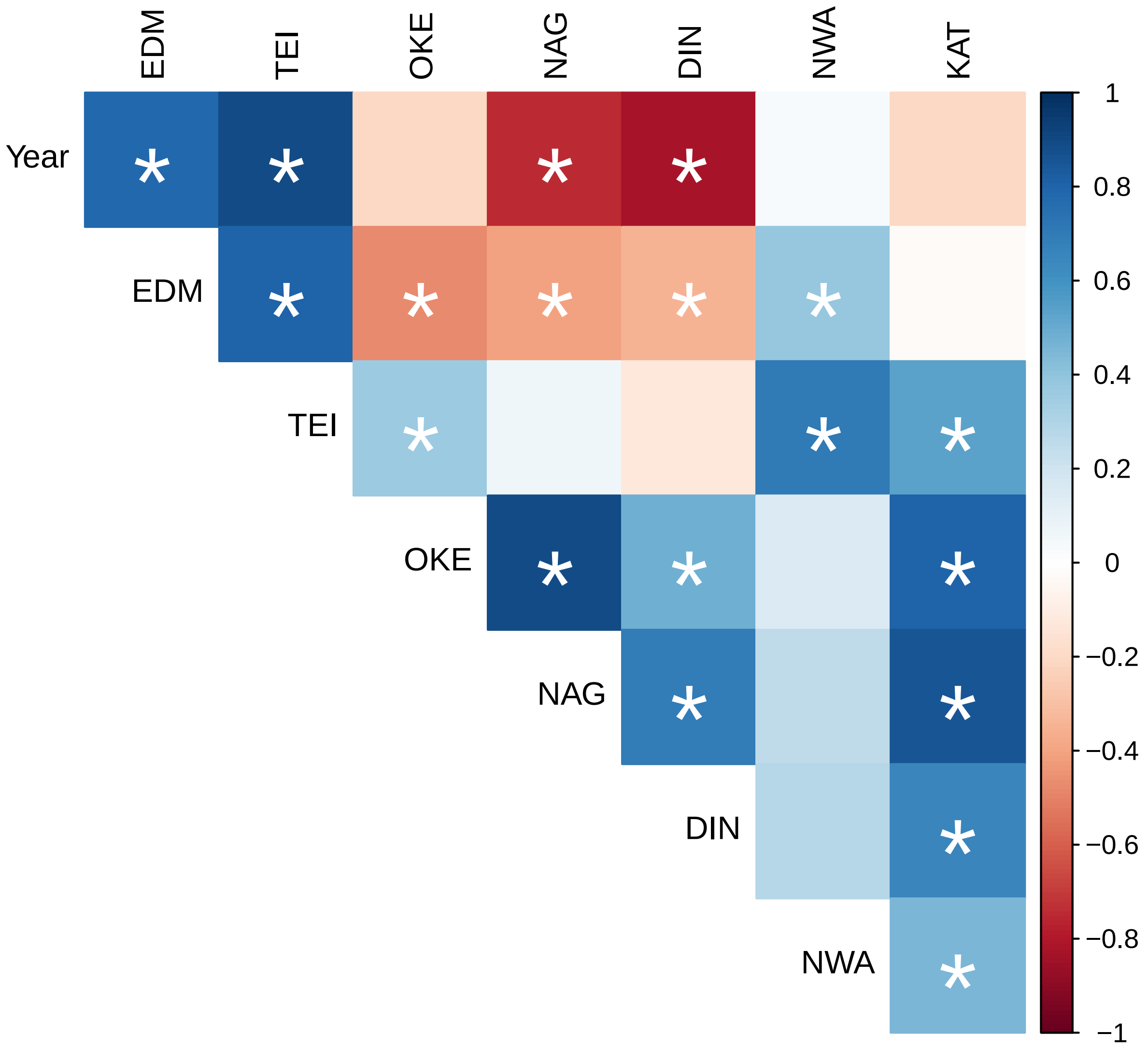

Figure S1.2: Post-hoc correlation analysis of annual population sizes in different study sites. The color gradient visualizes the Pearson correlation coefficient for the annual number of females in two populations (blue = positive correlations, red = negative correlations). A white star marks relationships for which the p-value of the correlation test was below 0.05. Note that each correlation test was done for all the years available for the specific pair of populations, and not just for the sequence of years common to the study periods of all populations. Furthermore, the correlation analysis was performed on the posterior median estimates of population size and not on a sample-by-sample basis. For that reason, there is no uncertainty displayed in this plot, and the correlation coefficients with year (top row) are not identical to those calculated as part of the trend-analysis (Table S1.3).

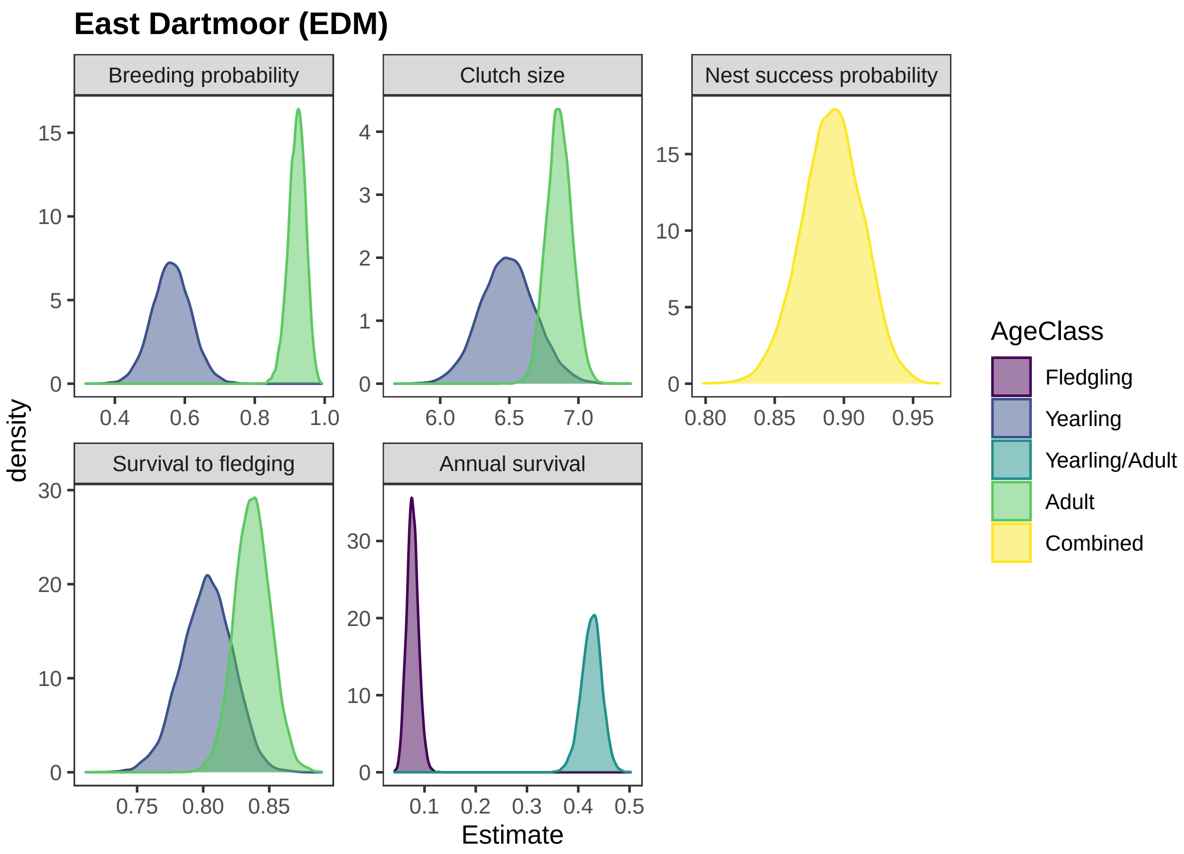

Figure S1.3: Posterior distributions for estimates of time-average vital rates by age class for the East Dartmoor (EDM) population. Reproductive parameters are estimated for yearlings and adults, while survival parameters are estimated for fledglings (young-of-the-year) and a joint yearling/adult class. Nest success probability was estimated independent of age class.

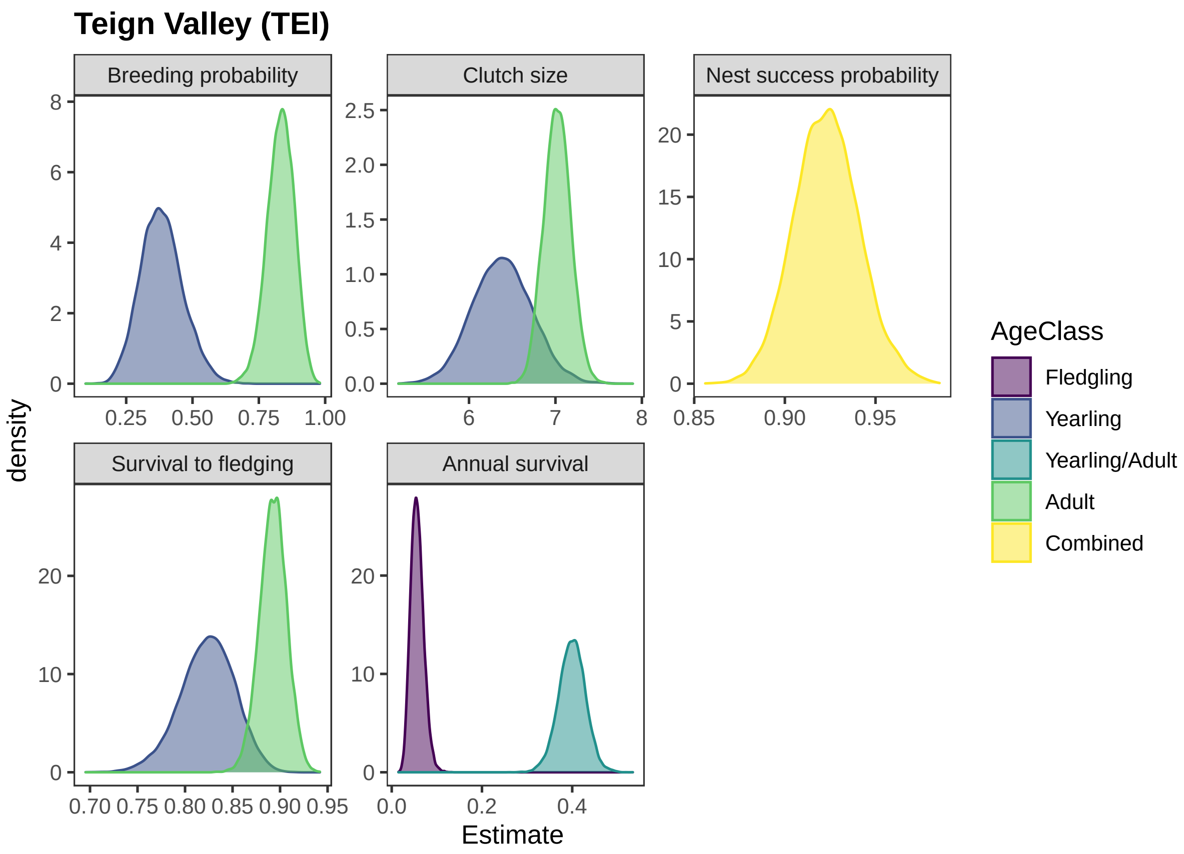

Figure S1.4: Posterior distributions for estimates of time-average vital rates by age class for the Teign Valley (TEI) population. Reproductive parameters are estimated for yearlings and adults, while survival parameters are estimated for fledglings (young-of-the-year) and a joint yearling/adult class. Nest success probability was estimated independent of age class.

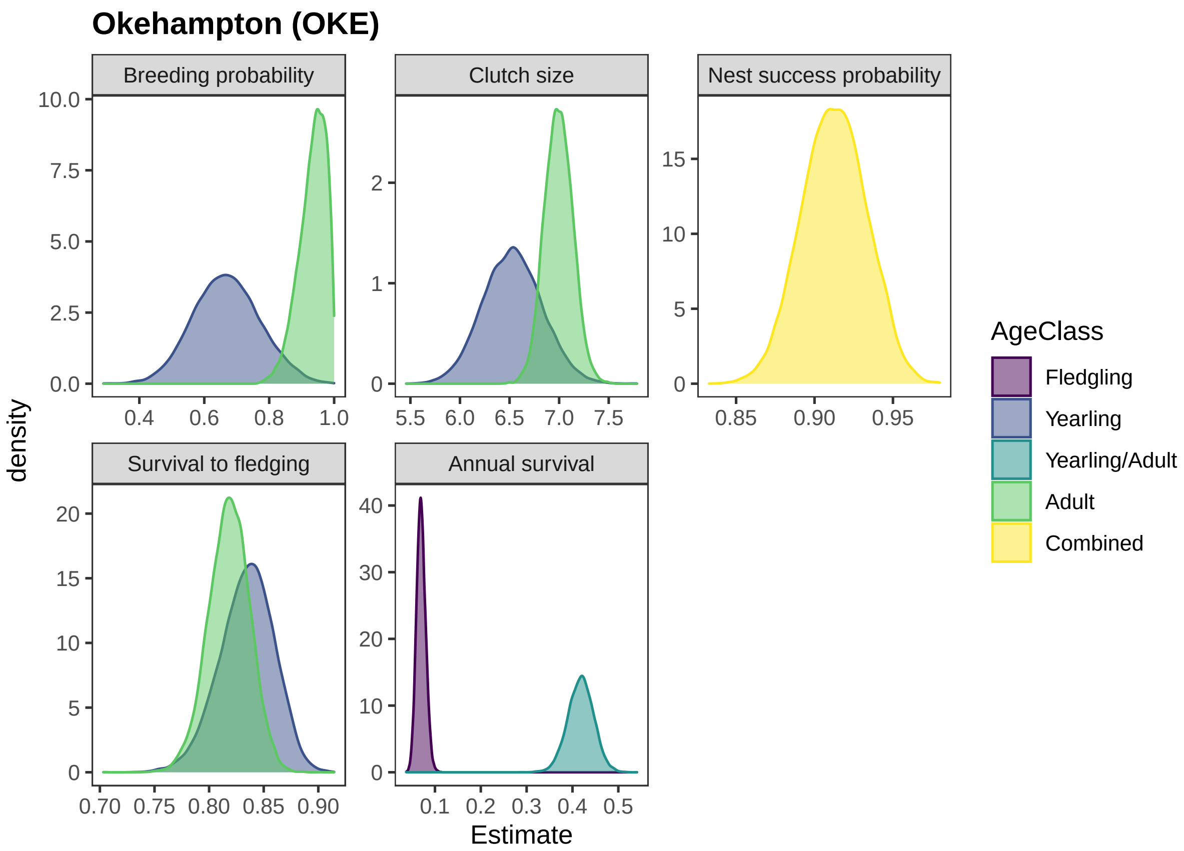

Figure S1.5: Posterior distributions for estimates of time-average vital rates by age class for the Okehampton (OKE) population. Reproductive parameters are estimated for yearlings and adults, while survival parameters are estimated for fledglings (young-of-the-year) and a joint yearling/adult class. Nest success probability was estimated independent of age class.

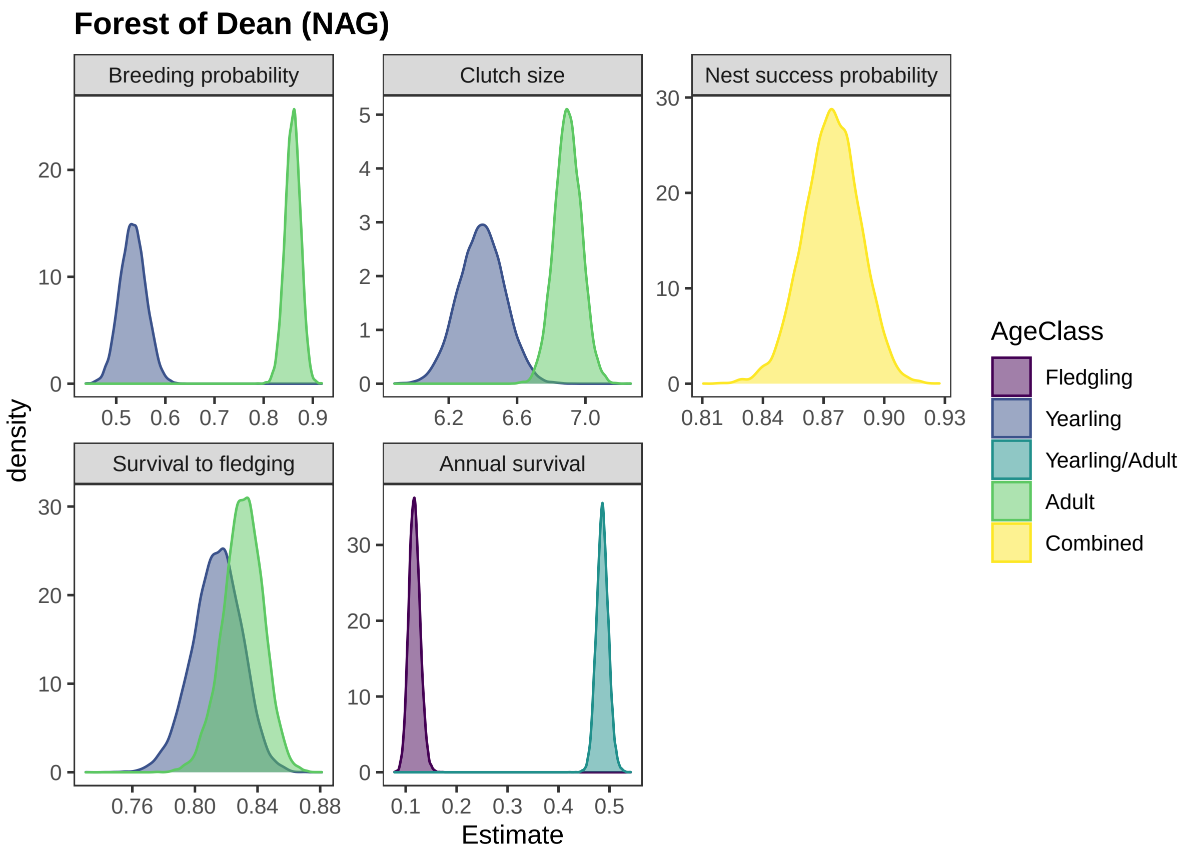

Figure S1.6: Posterior distributions for estimates of time-average vital rates by age class for the Forest of Dean (NAG) population. Reproductive parameters are estimated for yearlings and adults, while survival parameters are estimated for fledglings (young-of-the-year) and a joint yearling/adult class. Nest success probability was estimated independent of age class.

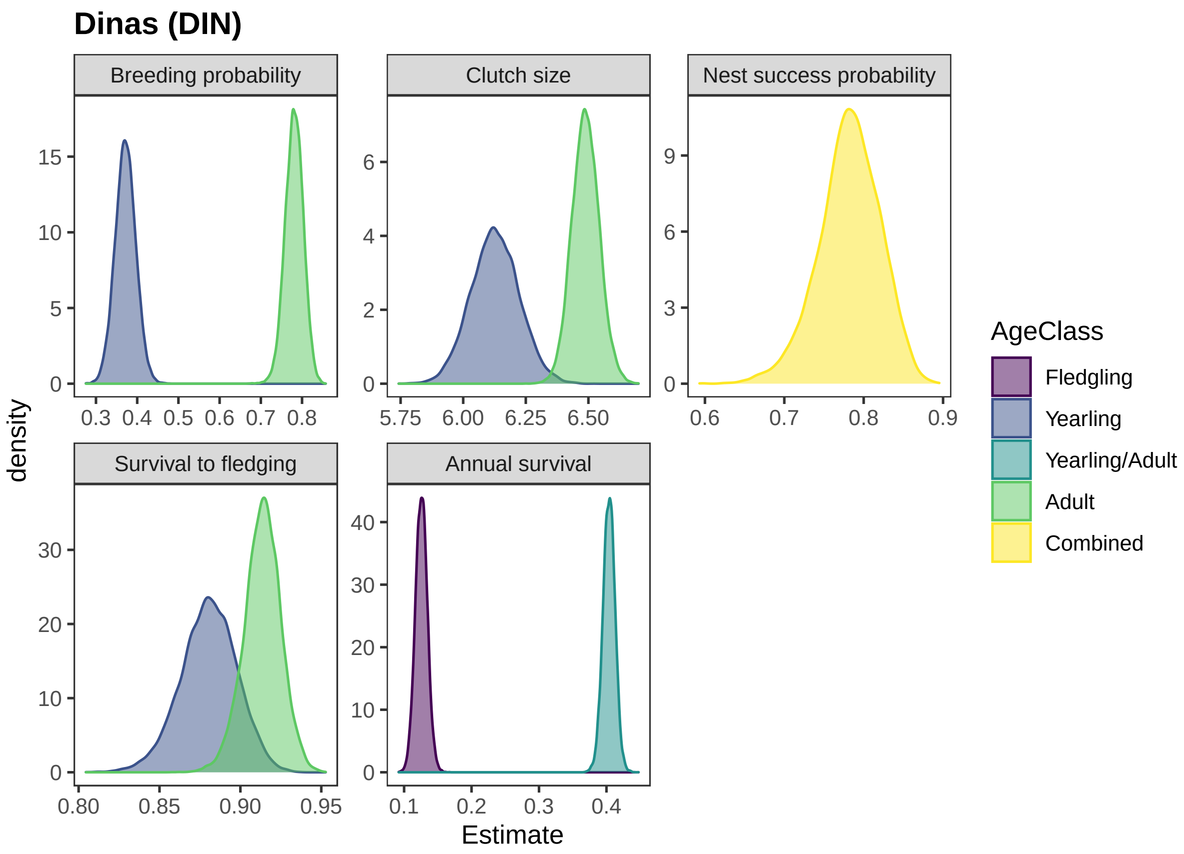

Figure S1.7: Posterior distributions for estimates of time-average vital rates by age class for the Dinas (DIN) population. Reproductive parameters are estimated for yearlings and adults, while survival parameters are estimated for fledglings (young-of-the-year) and a joint yearling/adult class. Nest success probability was estimated independent of age class.

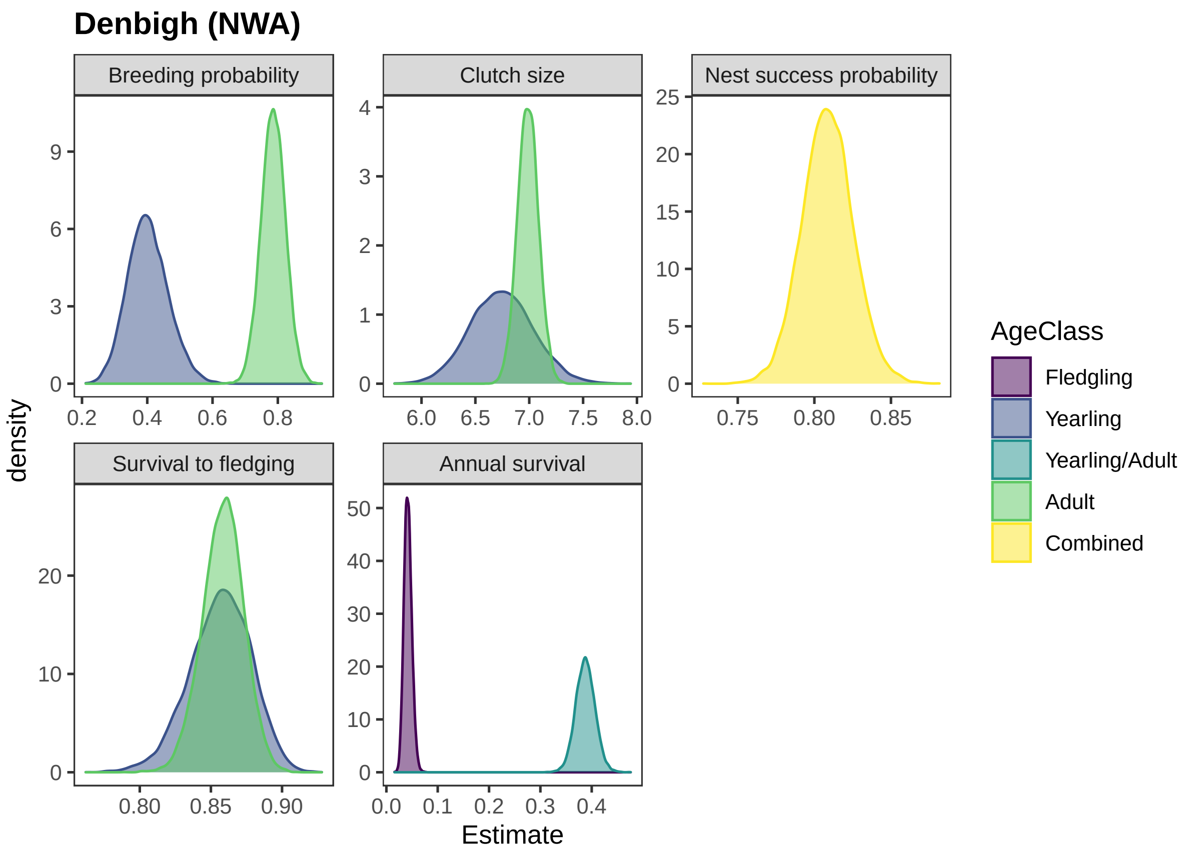

Figure S1.8: Posterior distributions for estimates of time-average vital rates by age class for the Denbigh (NWA) population. Reproductive parameters are estimated for yearlings and adults, while survival parameters are estimated for fledglings (young-of-the-year) and a joint yearling/adult class. Nest success probability was estimated independent of age class.

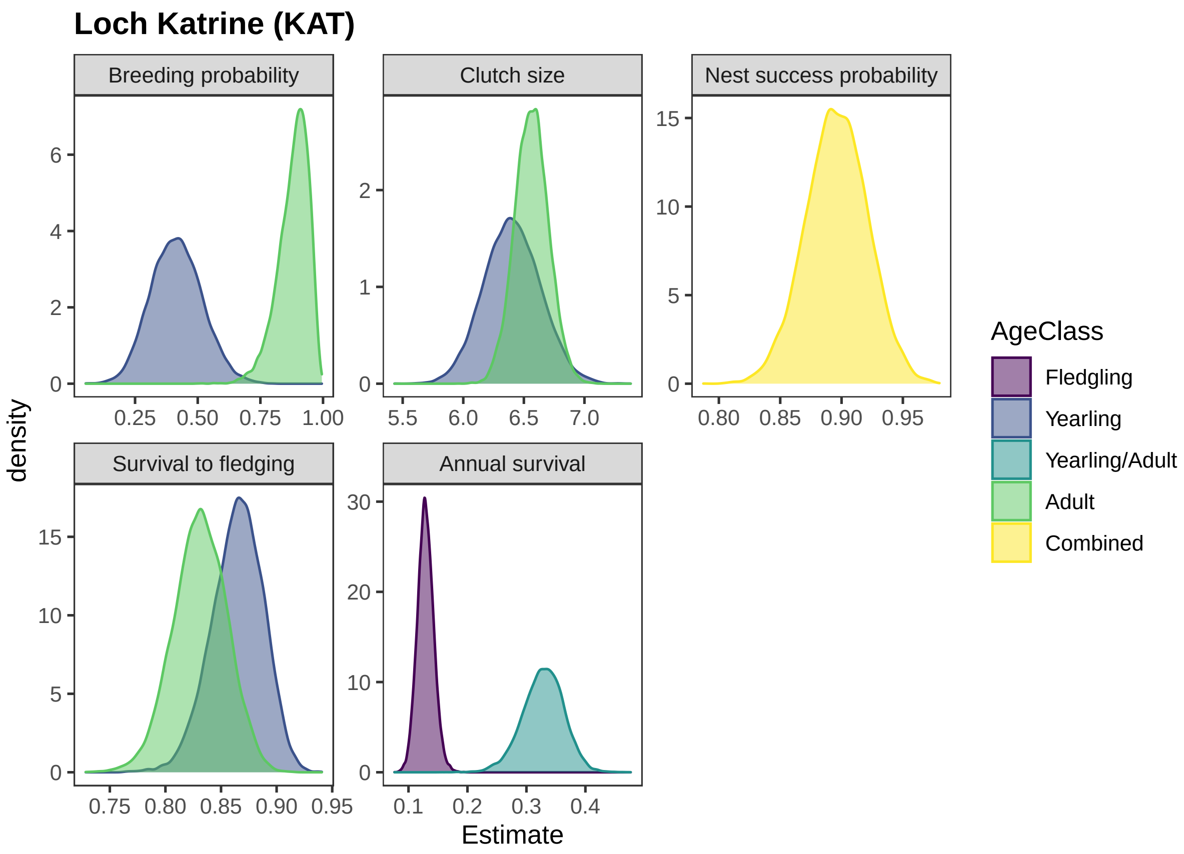

Figure S1.9: Posterior distributions for estimates of time-average vital rates by age class for the Loch Katrine (KAT) population. Reproductive parameters are estimated for yearlings and adults, while survival parameters are estimated for fledglings (young-of-the-year) and a joint yearling/adult class. Nest success probability was estimated independent of age class.

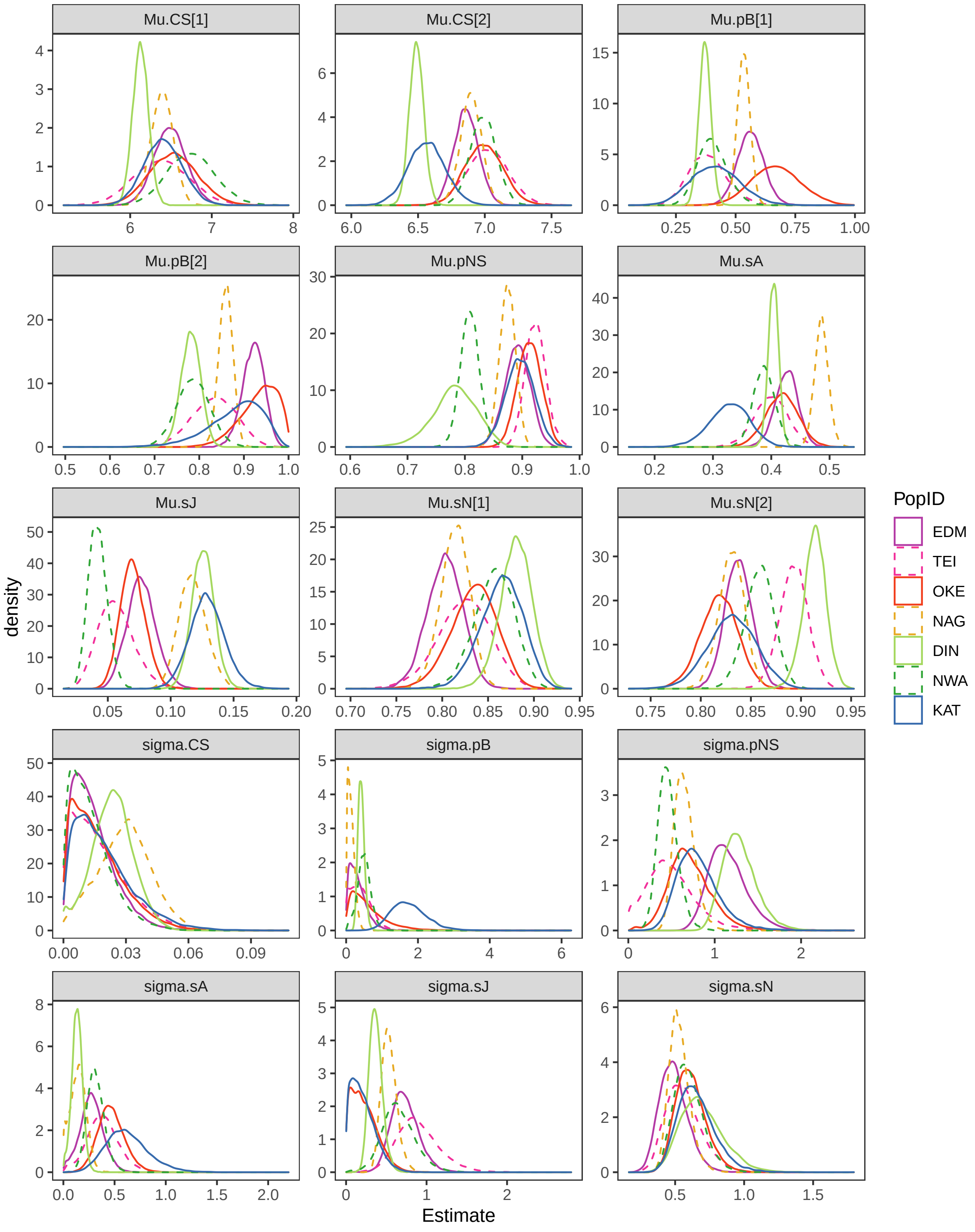

Figure S1.10: Posterior distributions for estimates of time-average vital rates by age class for all populations. Parameters names are as defined in Table S1.1.

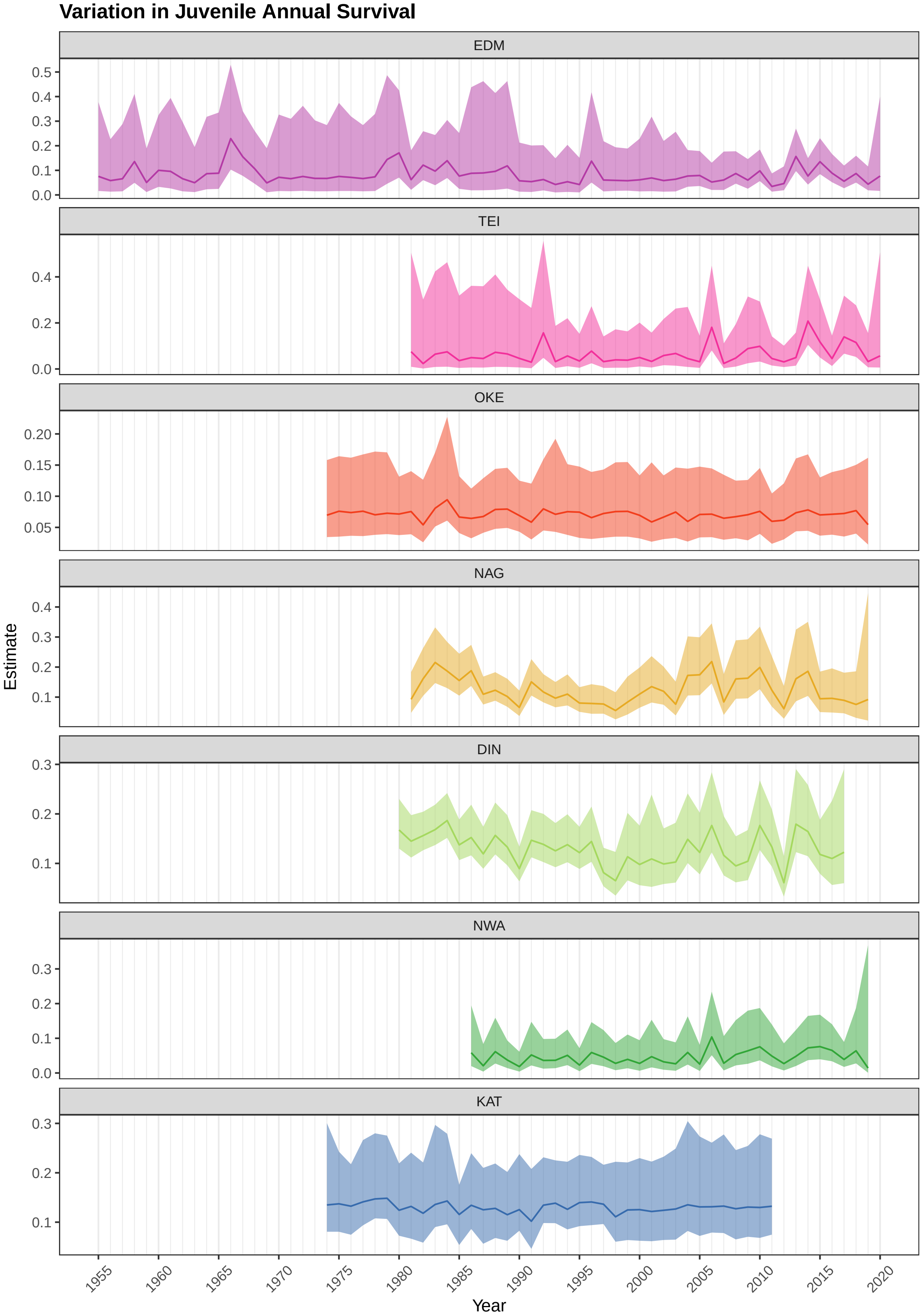

Figure S1.11: Among-year variation in juvenile annual survival (sJ_t) over the course of the study period for each population. Solid lines represent posterior medians, ribbons mark 95% credible intervals.

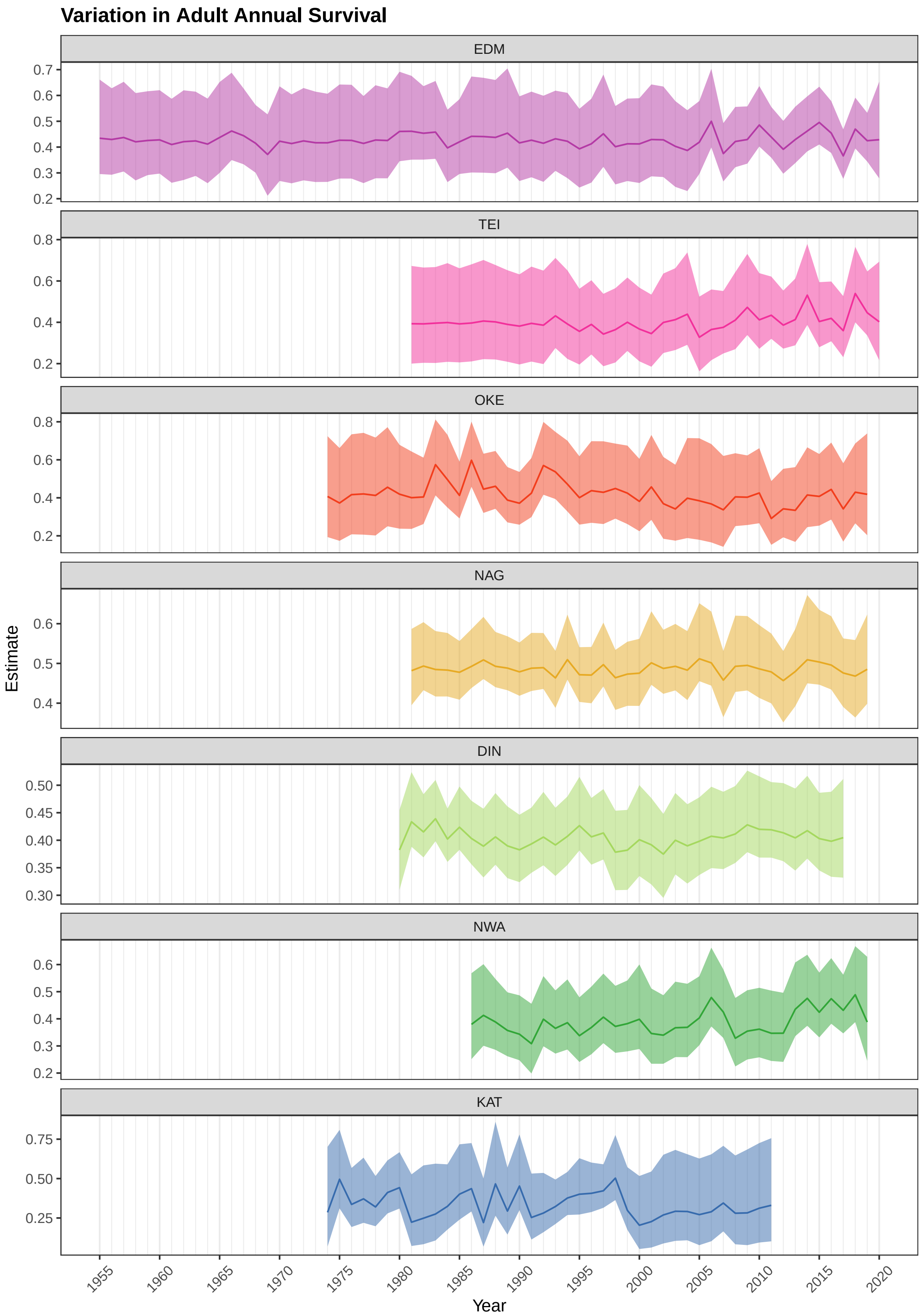

Figure S1.12: Among-year variation in adult annual survival (sA_t) over the course of the study period for each population. Solid lines represent posterior medians, ribbons mark 95% credible intervals.

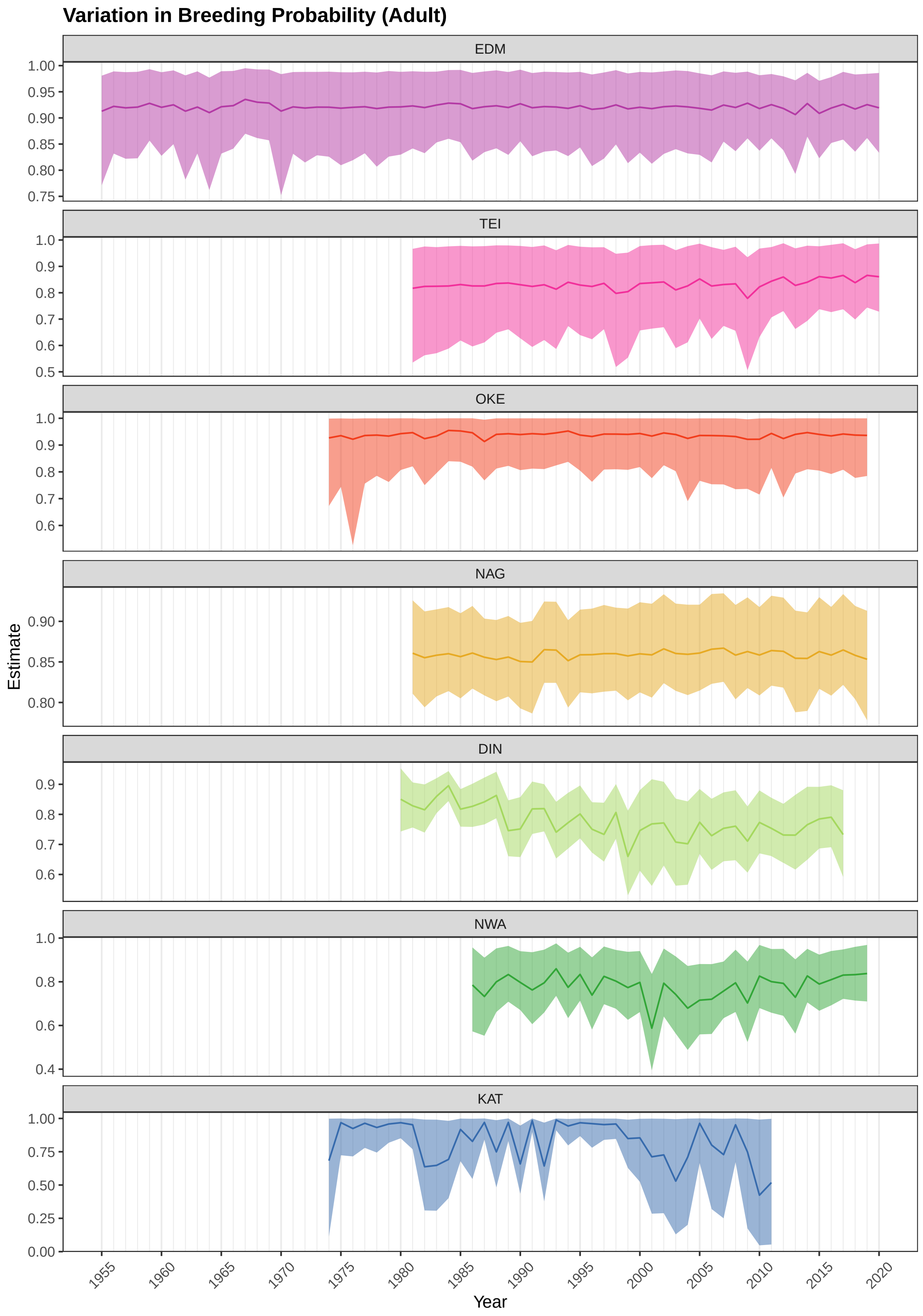

Figure S1.13: Among-year variation in adult breeding probability ($pB_{A,t}$) over the course of the study period for each population. Solid lines represent posterior medians, ribbons mark 95% credible intervals. Temporal variation in yearling breeding probability follows the same pattern as year random effects and environmental effects are shared.

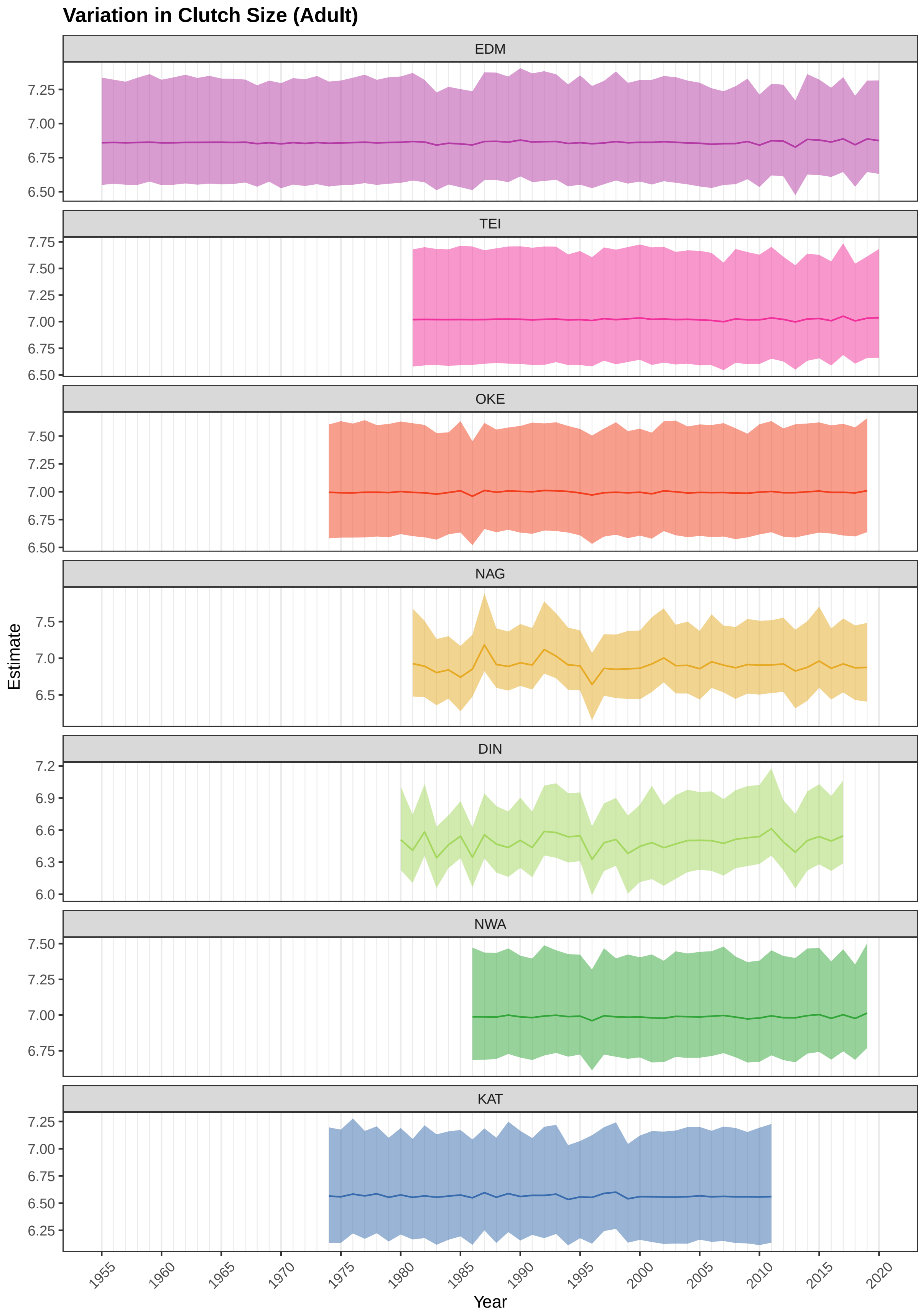

Figure S1.14: Among-year variation in adult clutch size ($CS_{A,t}$) over the course of the study period for each population. Solid lines represent posterior medians, ribbons mark 95% credible intervals. Temporal variation in yearling yearling clutch size follows the same pattern as year random effects and environmental effects are shared.

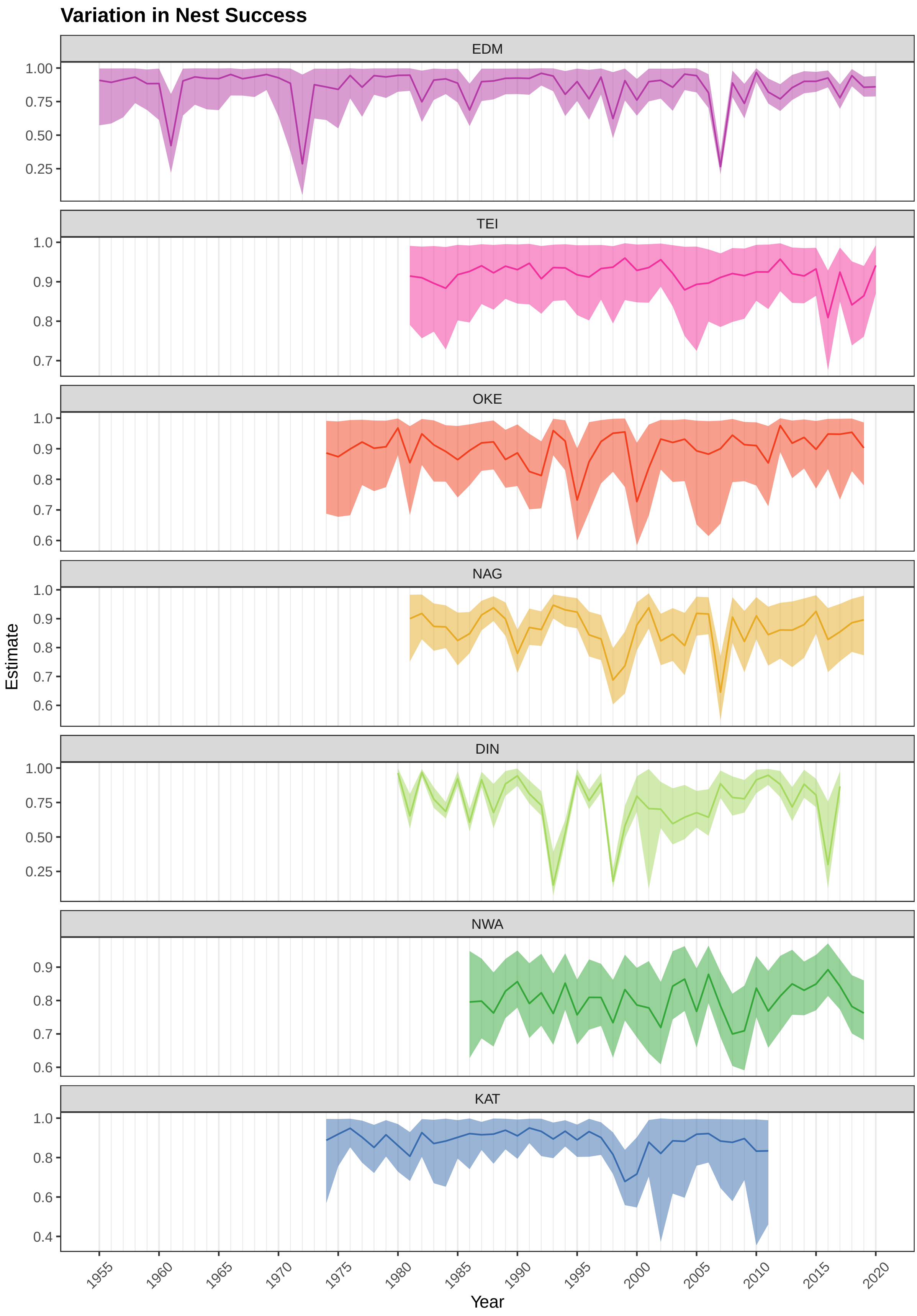

Figure S1.15: Among-year variation in nest success probability (pNS_t) over the course of the study period for each population. Solid lines represent posterior medians, ribbons mark 95% credible intervals.

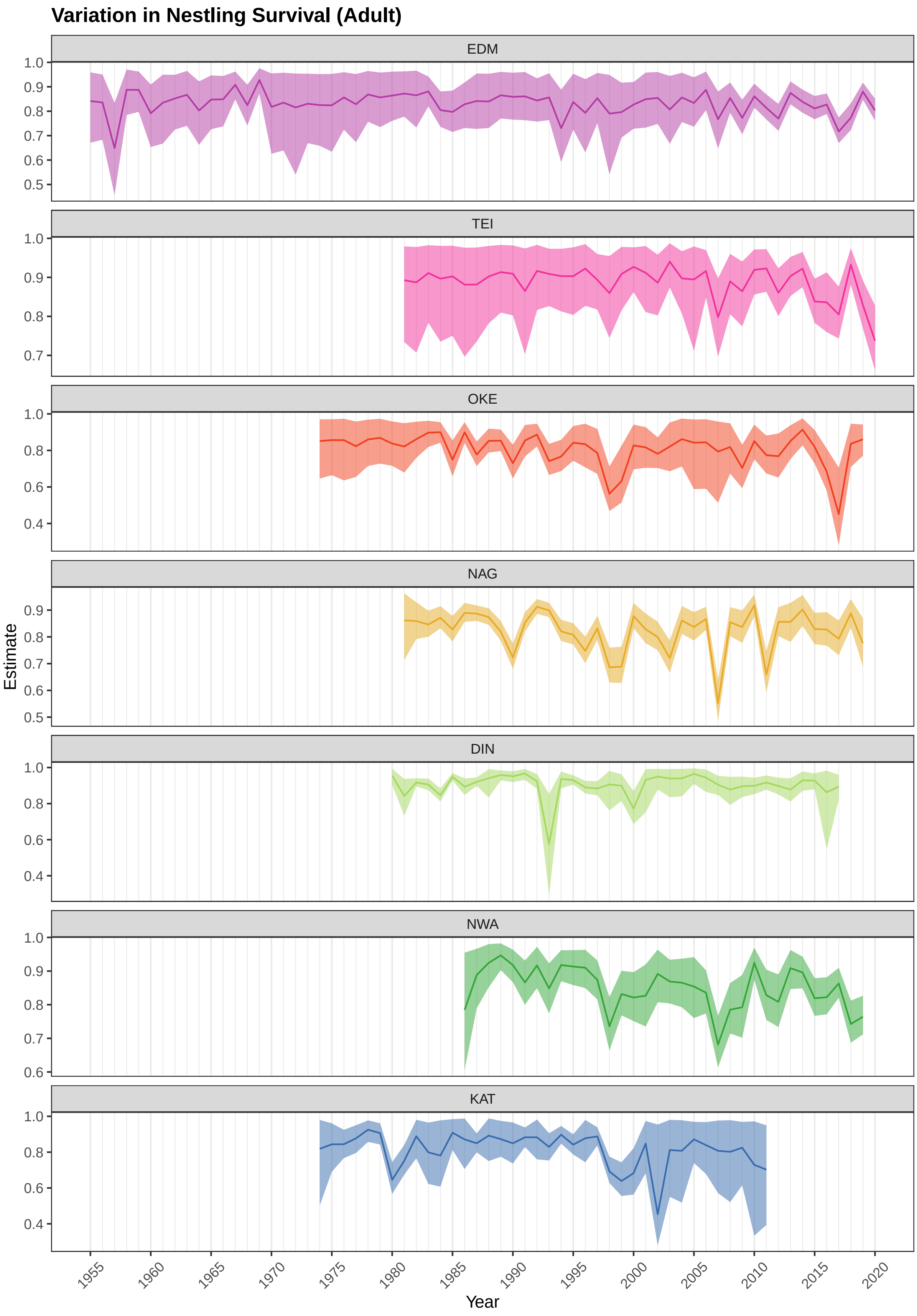

Figure S1.16: Among-year variation in nestling survival of chicks with adult mothers ($sN_{A,t}$) over the course of the study period for each population. Solid lines represent posterior medians, ribbons mark 95% credible intervals. Temporal variation in nestling survival with yearling mothers follows the same pattern as year random effects and environmental effects are shared.

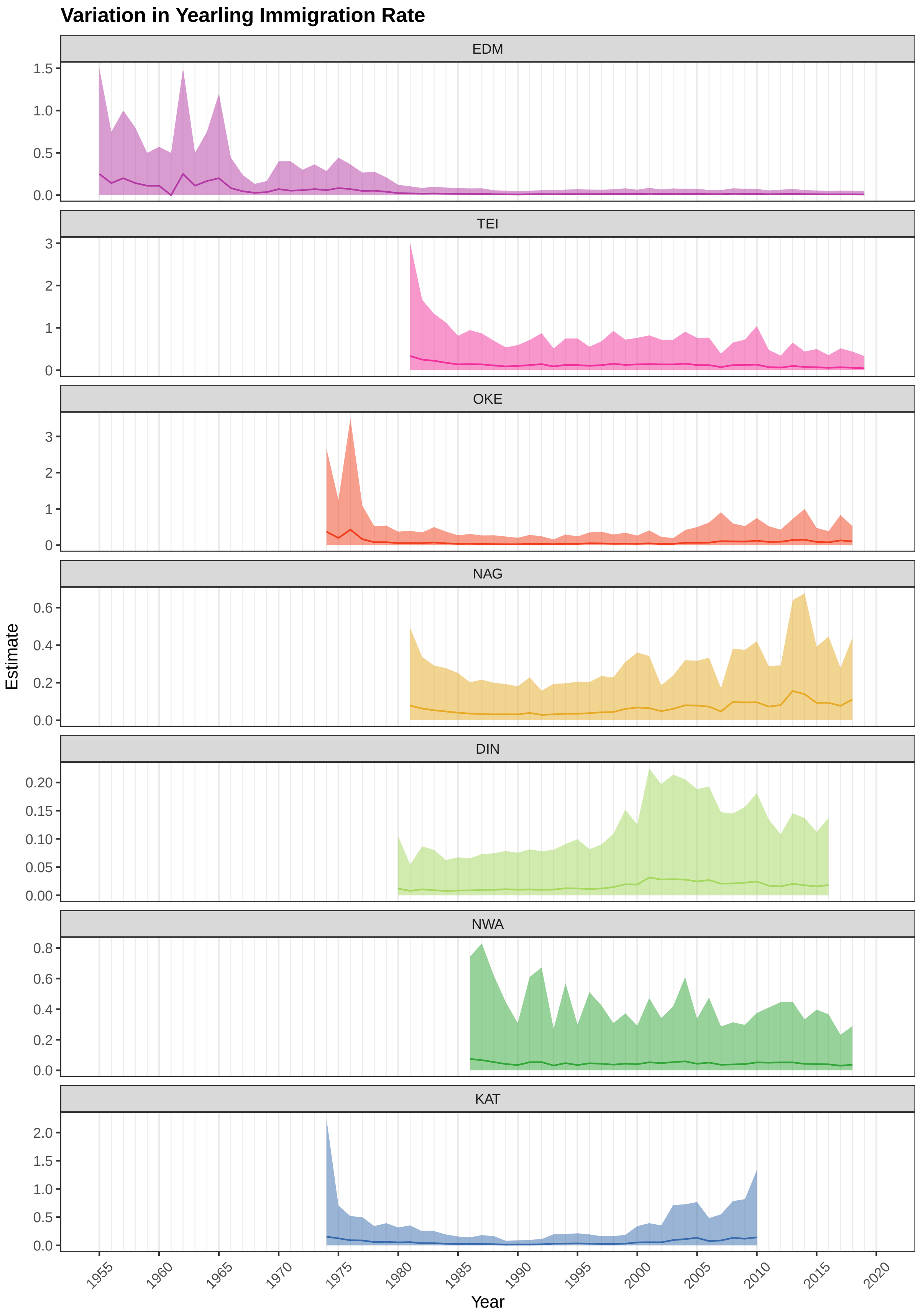

Figure S1.17: Among-year variation in yearling immigration rate ($imm_{Y,t}$) over the course of the study period for each population. Solid lines represent posterior medians, ribbons mark 95% credible intervals.

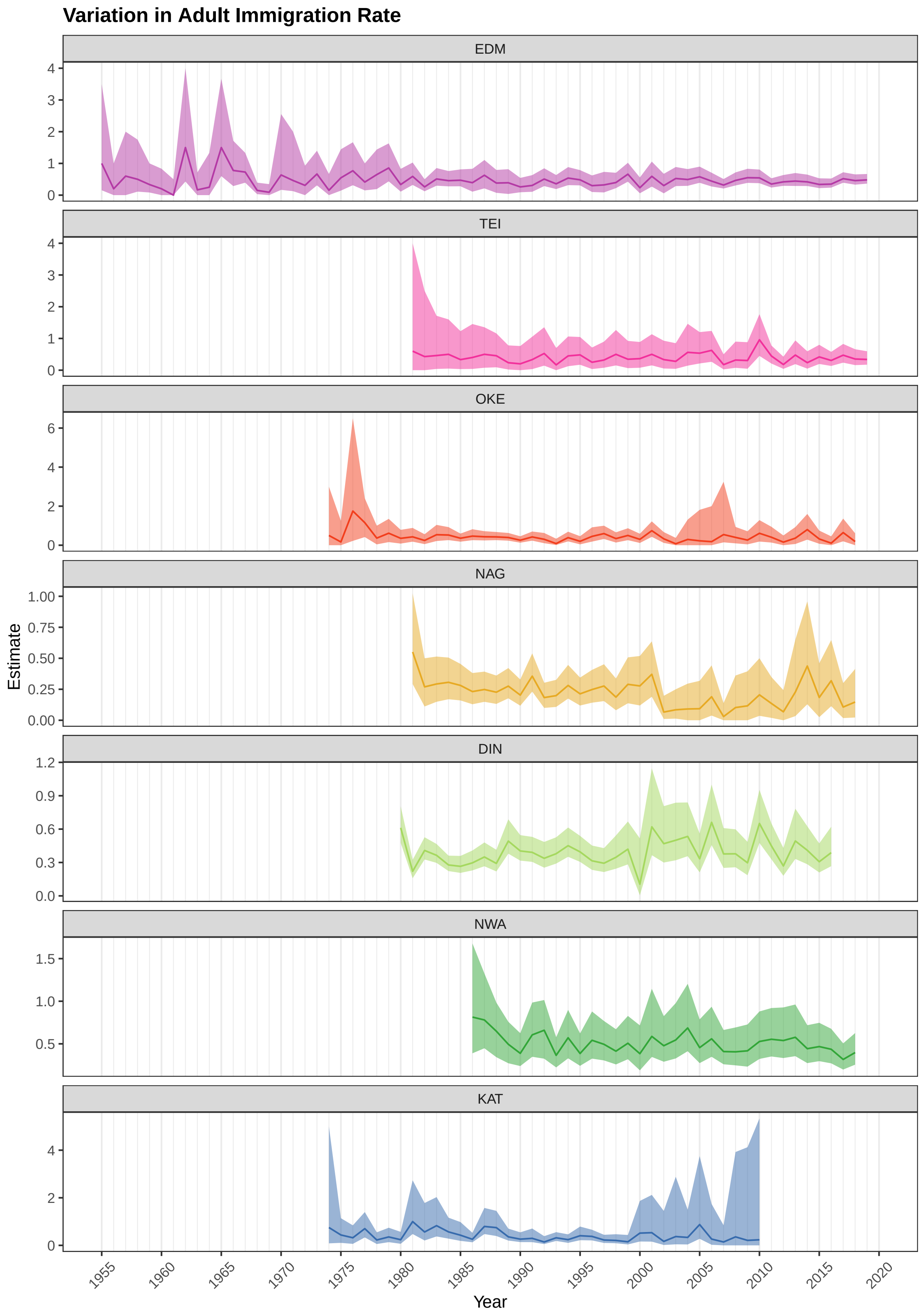

Figure S1.18: Among-year variation in adult immigration rate ($imm_{A,t}$) over the course of the study period for each population. Solid lines represent posterior medians, ribbons mark 95% credible intervals.

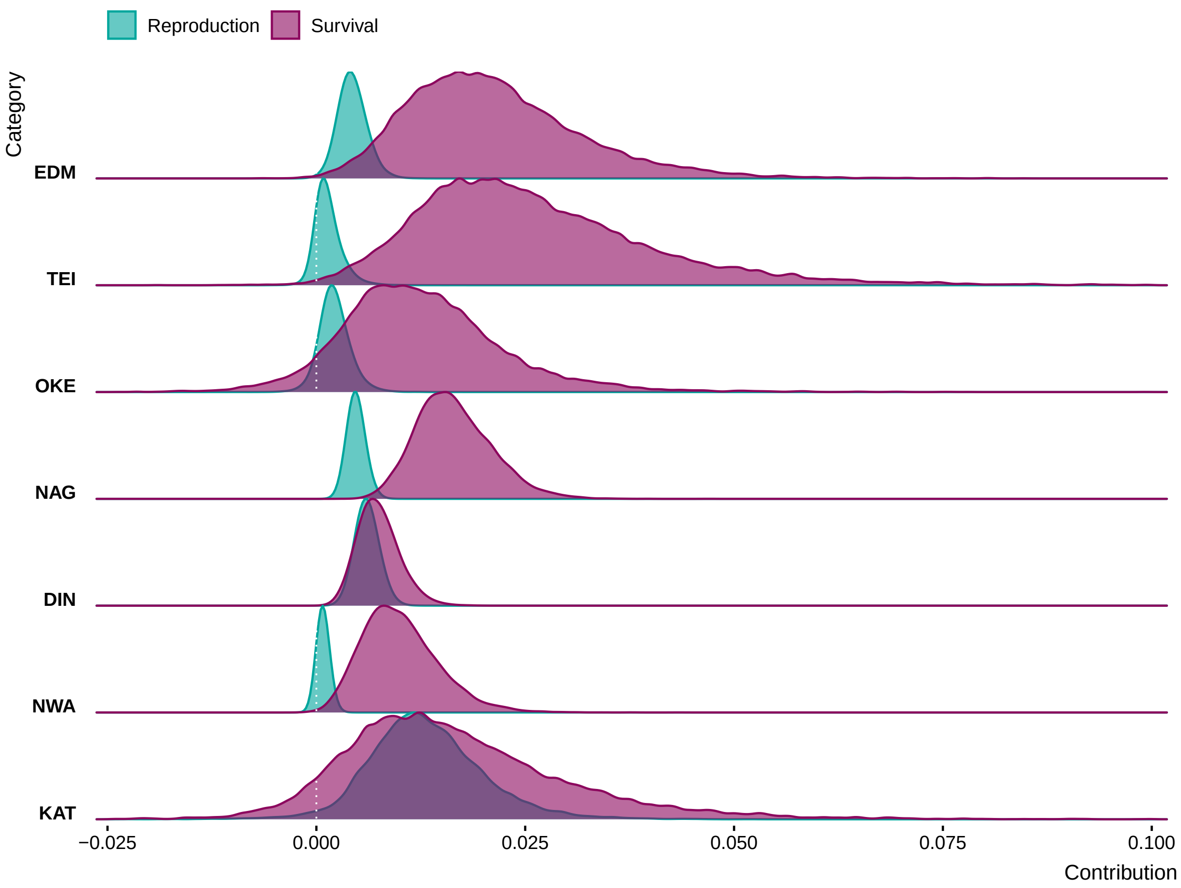

Figure S1.19: Posterior distributions of the contributions of reproduction (breeding probability, nest success probability, nestling survival probability) versus survival (juvenile and adult annual survival) to variation in realized annual population growth rates. Contributions from local population structure and from immigration are omitted here.

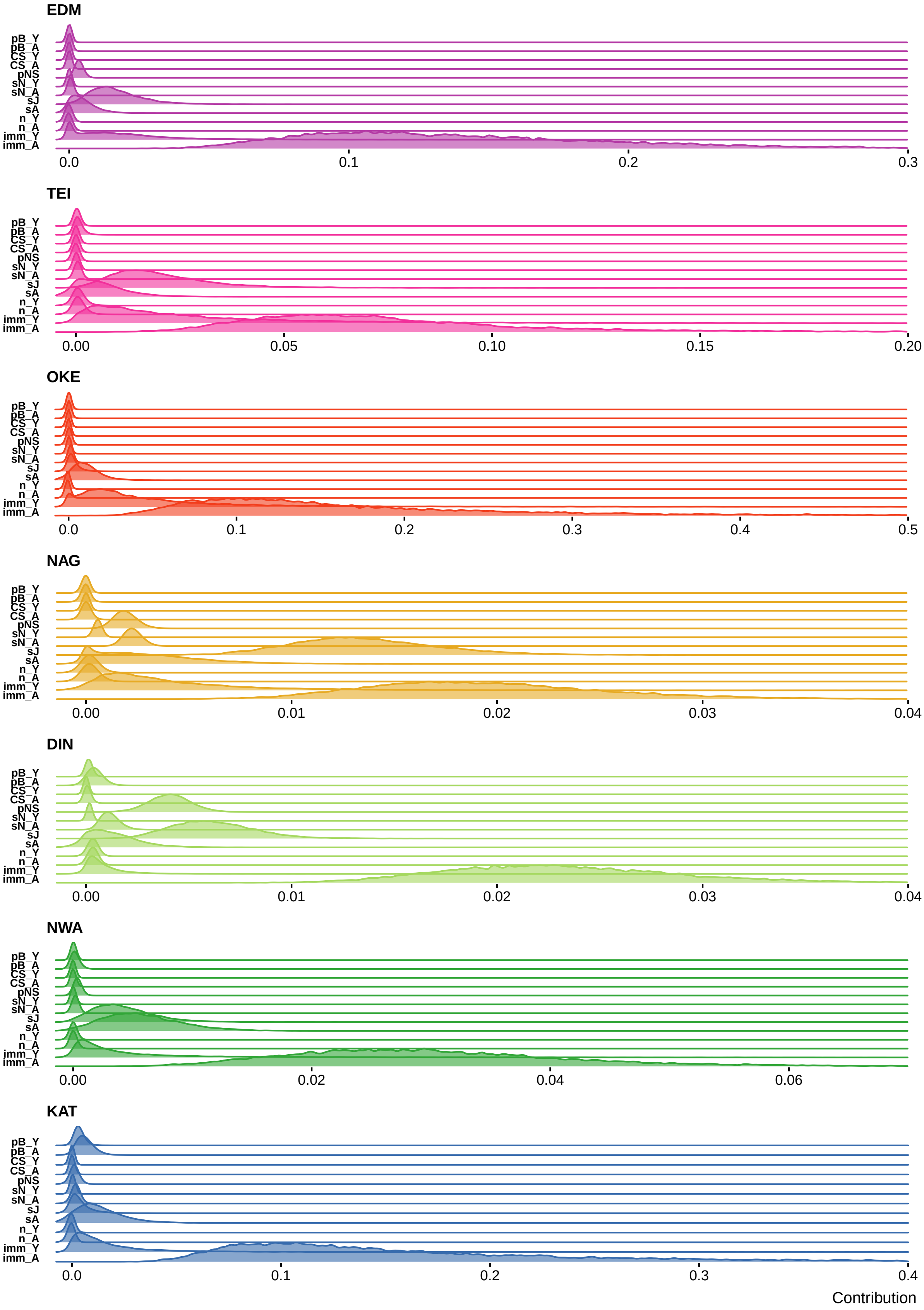

Figure S1.20: Posterior distributions of the contributions of age-specific vital rates and population structure components to variation in realized annual population growth rates. Parameter definitions are as outlined in Table S1.1. x-axes have been restricted (removing outliers) to facilitate visualization.

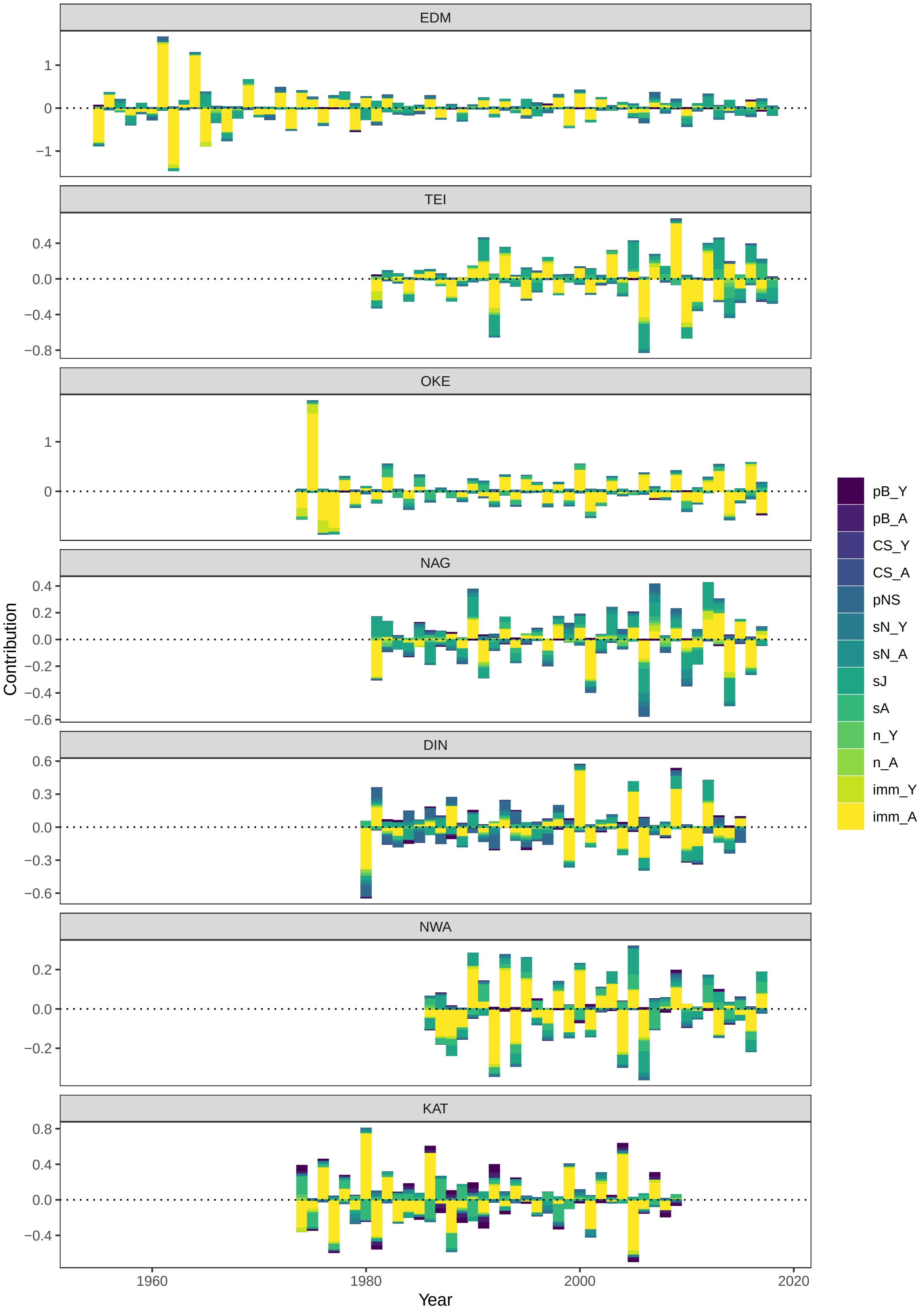

Figure S1.21: Posterior medians of stacked contributions of vital rates and age structure among the local population and immigrants to year-by-year changed in annual population growth rate over time for each study population. The sum of all contributions approximates the total rate of change in population size from one year to the next.

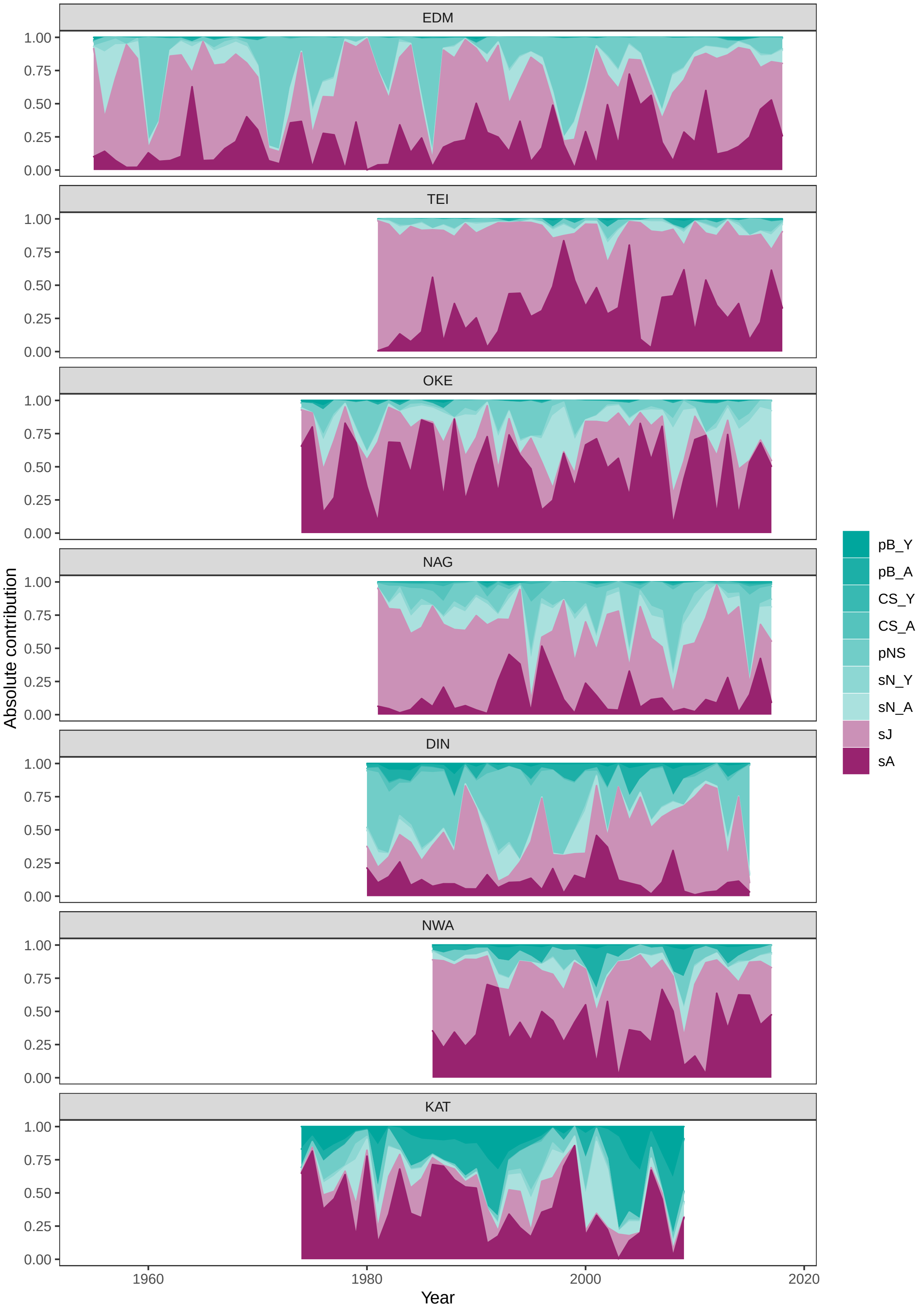

Figure S1.22: Posterior medians of stacked relative contributions of vital rates within the local population to year-by-year changed in annual population growth rate over time for each study population. Contributions are standardized by taking the absolute value, and scaling it such that the sum of all contributions equals 1.

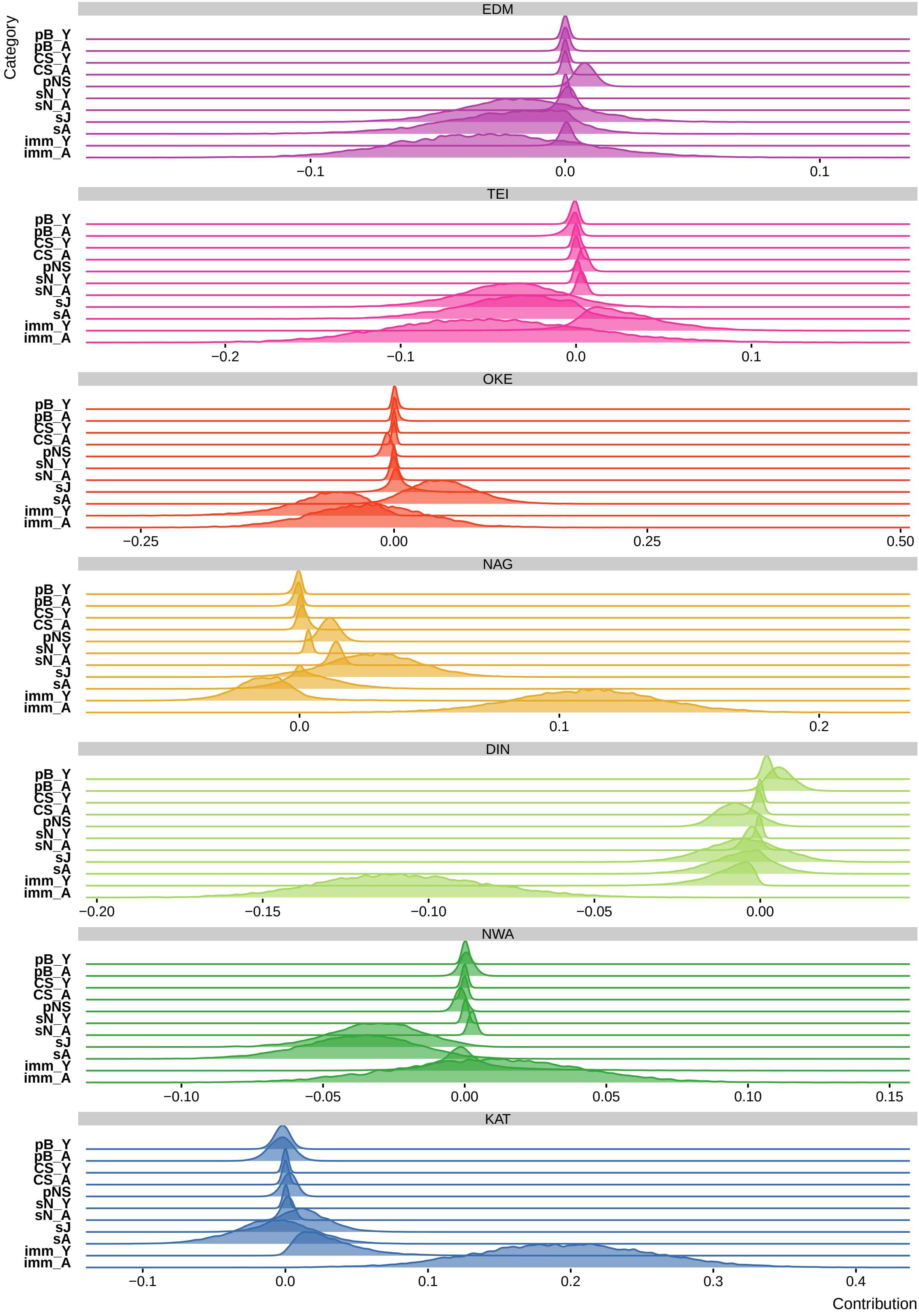

Figure S1.23: Posterior distributions of the contributions of age-specific vital rates (incl. immigration rates) to changes in long-term population trends. Parameter definitions are as outlined in Table S1.1.

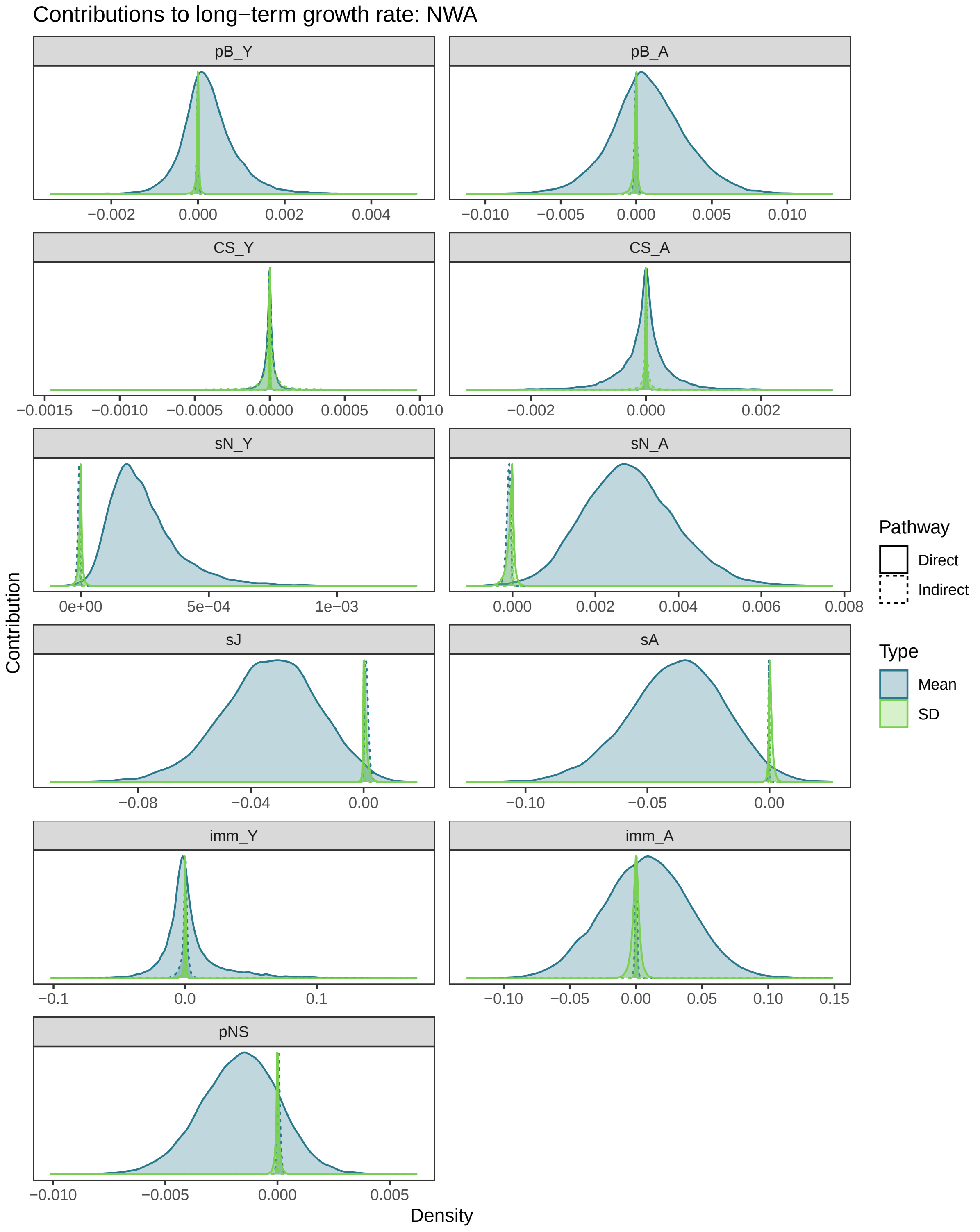

Figure S1.24: Posterior distributions of the contributions of age-specific vital rates (incl. immigration rates) to changes in long-term population trends. Contributions are partitioned into direct (solid lines) and indirect effects (through perturbation of population structure, dashed lines) of changes in the mean of (blue) and variation in (green) vital rates. Parameter definitions are as outlined in Table S1.1. The figure depicts results for population NWA; results for the other six populations were consistent and very similar.

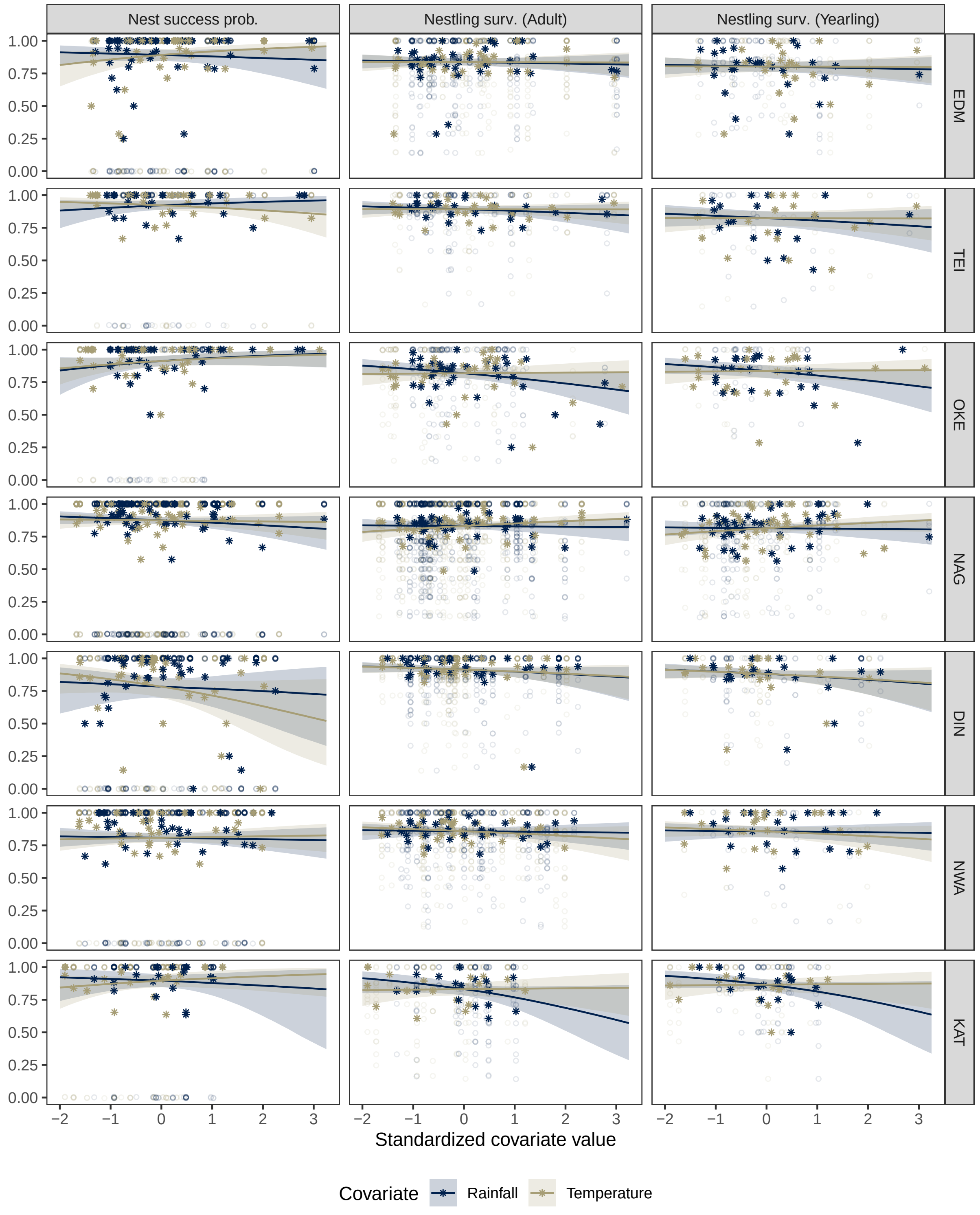

Figure S1.25: Predicted effects of rainfall (blue) and temperature (ochre) on nest success probability and nestling survival of the seven study populations (solid lines = posterior Medians, ribbons = 95% credible intervals). Rings represent raw data collected at the nest level, stars represent annual summaries of these data. Environmental covariates represented conditions during a 16-day window post hatching and are are plotted on a standardized scale.

S1.2 Tables

Table S1.1: Overview over the parameters used in the integrated population model and LTRE analyses. "Parameter (text)" gives the notation used in text and equations (main text, supplementary material, code manual). "Parameter (code)" gives the notation used in the code files (note that depending on the code, age class is indicated as either `_Y`/`_A` or as `[1,]`/`[2,]` but those notations are equivalent). The time subscript t refers to the breeding season of year t for most parameters. The exceptions are sJ_t and sA_t, which constitute annual survival from the end of the breeding season in year t to the start of the breeding season in year $t + 1$.

Parameter (text)	Parameter (code)	Description
$N_{Y,t}$	`N_Y[t] / N[1,t]`	Number of yearling females in the population
$N_{A,t}$	`N_A[t] / N[2,t]`	Number of adult females in the population
$n_{Y,t}$	`n_Y[t]`	Proportion of yearling females in the population
$n_{A,t}$	`n_A[t]`	Proportion of yearling females in the population
$pB_{Y,t}$	`pB_Y[t] / pB[1,t]`	Breeding probability of yearling females
$pB_{A,t}$	`pB_A[t] / pB[2,t]`	Breeding probability of adult females
$CS_{Y,t}$	`CS_Y[t] / CS[1,t]`	Clutch size of yearling females
$CS_{A,t}$	`CS_A[t] / CS[2,t]`	Clutch size of adult females
pNS_t	`pNS[t]`	Nest success probability
$sN_{Y,t}$	`sN_Y[t] / sN[1,t]`	Nestling survival (mother = yearling)
$sN_{A,t}$	`sN_A[t] / sN[2,t]`	Nestling survival (mother = adult)
sJ_t	`sJ[t]`	Juvenile annual survival
sA_t	`sA[t]`	Adult annual survival
$Imm_{Y,t}$	`Imm_Y[t] / Imm[1,t]`	Number of yearling female immigrants
$Imm_{A,t}$	`Imm_A[t] / Imm[2,t]`	Number of adult female immigrants
$imm_{Y,t}$	`imm_Y[t]`	Immigration rate (yearling females)
$imm_{A,t}$	`imm_A[t]`	Immigration rate (adult females)

Table S1.2: This is a stand-in for supplementary Table S1.2, which is provided as a .csv file. The table contains summaries of estimates of vital rate parameters for all seven populations. Parameter summaries consist of posterior means, medians, and 95% credible intervals (*lowerCI* = 2.5% quantile, *upperCI* = 97.5% quantile). Parameter names in the .csv table are consistent with naming within model code).

Parameter	PopID	Mean	Median	lowerCI	upperCI

Check supplementary file "Table_S2.csv"

Table S1.3: This is a stand-in for supplementary Table S1.3, which is provided as a .csv file. The table contains summaries of post-hoc tests for time trends in population sizes and vital rates for all seven populations. Besides the quantities defined in Table S1.1, several other parameters appear here: Ntot = total population size, Btot = breeding population size, Rtot = total number of fledglings produced per year, Immtot = total number of immigrants, immtot = total immigration rates (immigrants per local bird), YAratio = ratio of yearlings to adults in the population, Rrate = reproductive rate (average number of fledglings produced per breeding female). Test summaries are presented as posterior means, medians, and 95% credible intervals (*r_lowerCI* = 2.5% quantile, *r_upperCI* = 97.5% quantile) of Pearson correlation coefficients (between the quantity in question and year). The column *Evidence* contains * for correlation coefficients whose 95% CI did not overlap 0, and - for all others.

PopID	Parameter	r_mean	r_lowerCI	r_median	r_upperCI	Evidence

Check supplementary file ”Table_S3.csv”

Table S1.4: This is a stand-in for supplementary Table S1.4, which is provided as a .csv file. The table contains summaries of estimates of the slopes of environmental effects for all seven populations. beta1.pNS = effect of temperature on nest success, beta1.sN = effect of temperature on nestling survival, beta2.pNS = effect of rainfall on nest success, beta2.sN = effect of rainfall on nestling survival, beta3.sJ = effect of rainfall on juvenile annual survival. Parameter summaries consist of posterior medians, 95%, 90%, and 50% credible intervals (*lCI95* = 2.5% quantile, *uCI95* = 97.5% quantile, *lCI90* = 5% quantile, *uCI90* = 95% quantile, *lCI50* = 25% quantile, *uCI50* = 75% quantile). The column *Evidence* contains ** for effect posteriors whose 90% CI does not overlap 0, * for effect posteriors whose 50% CI does not overlap 0, and - for all others.

Parameter	PopID	lCI95	lCI90	lCI50	Median	uCI50	uCI90	uCI95	Evidence

Check supplementary file ”Table_S4.csv”

S2 Model Assessment

We checked the general fit and performance of the IPM fit to all seven datasets using a combination of three approaches. First, we compared model predictions to relevant observational data. Second, we compared posterior distributions of parameter estimates obtained from the IPM to equivalent estimates obtained from independently-run models. Third, we used the posterior medians of population sizes and posterior estimates to run stochastic simulations of population dynamics and assessed whether these could be considered realistic.

S2.1 Comparing predictions to data

We compared posterior predictions from the IPMs to observational data for several quantities:

1. Annual breeding population size ($B_{tot,t}$)
2. Annual number of breeding immigrants ($ImmB_{Y,t} + ImmB_{Y,t}$)
3. Year- and age-specific clutch size ($CS_{a,t}$)
4. Year-specific proportion of successful nests (pNS_t)
5. Year- and age-specific proportion of surviving nestlings ($sN_{a,t}$)
6. Year- and age-specific reproductive output ($Juv_{a,t}/B_{a,t}$)

The comparisons are presented graphically for each population in the following Figures S2.1-S2.7. Overall, there was good agreement between model estimates and observational data, providing no evidence of severe lack of fit and/or substantial directional bias.

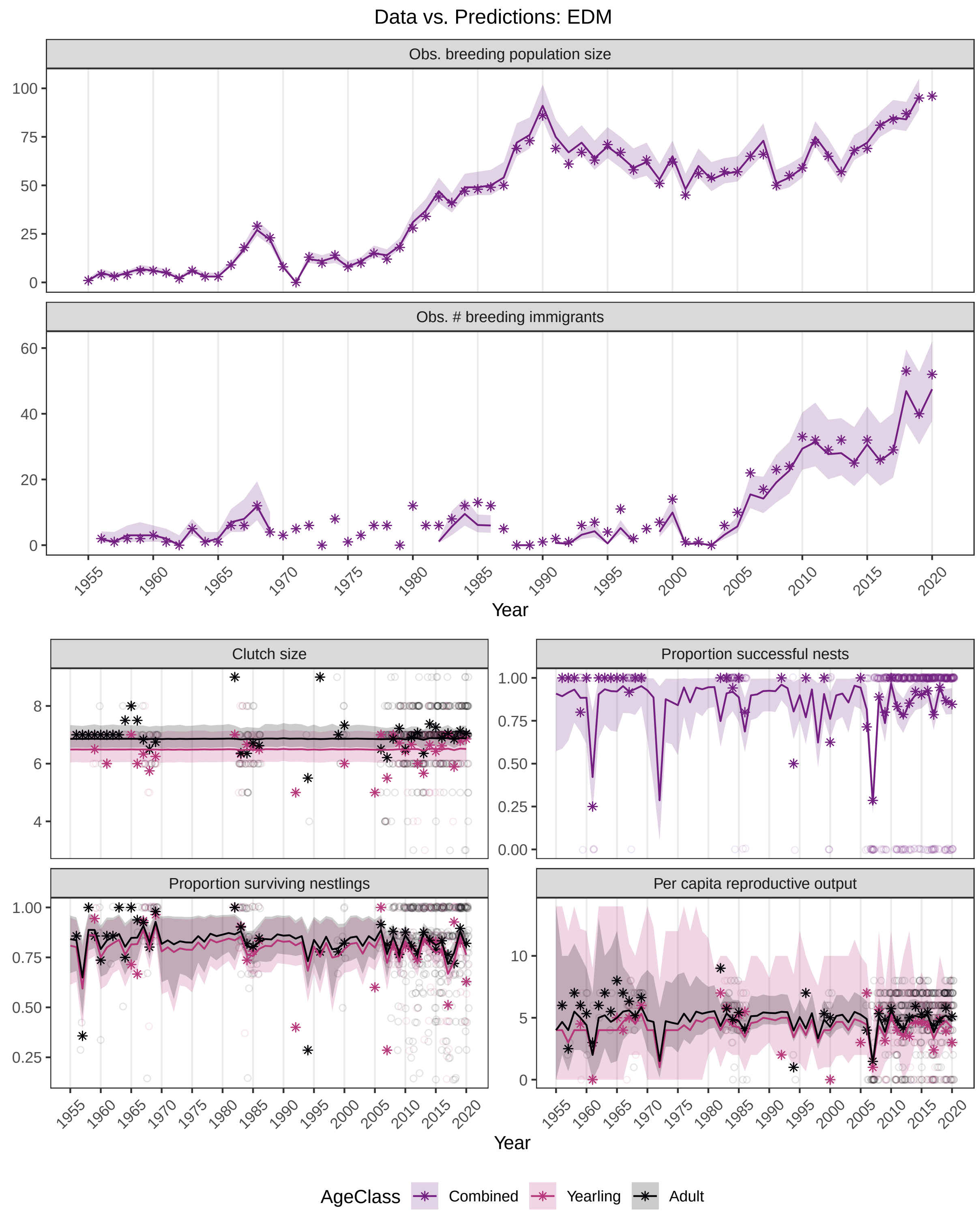

Figure S2.1: Comparison of model predictions to observational data for a variety of quantities for population EDM. Stars represent data values, solid lines are posterior median estimates, ribbons mark the associated 95% credible intervals. Purple is used for quantities pertaining to all age classes, while black indicates adult females and pink indicates yearling females. Note predictions of the number of breeding immigrants (second panel from the top) were not plotted in years in which the proportion of immigrant birds captured was unknown a priori and needed to be estimated within the IPM.

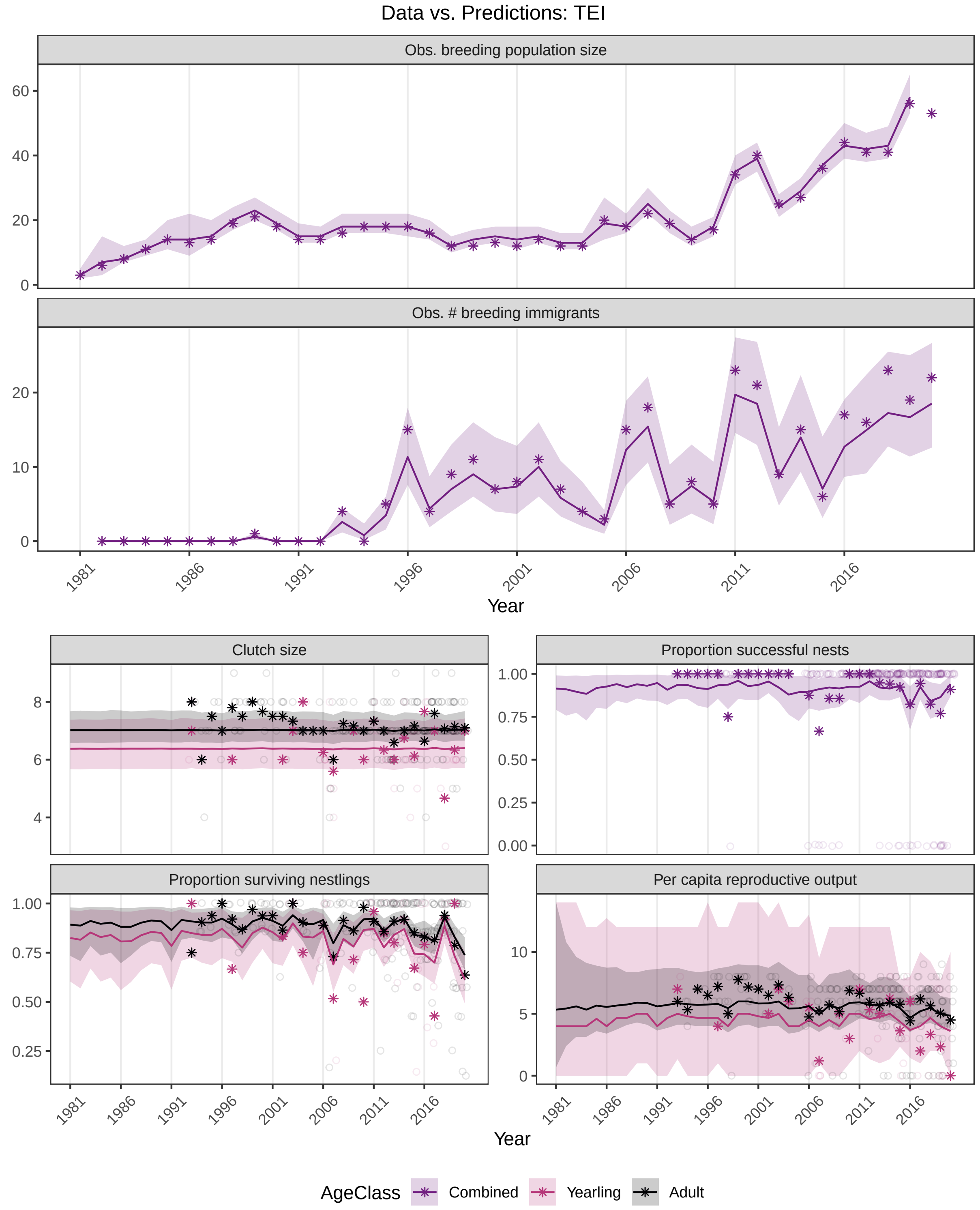

Figure S2.2: Comparison of model predictions to observational data for a variety of quantities for population TEI. Stars represent data values, solid lines are posterior median estimates, ribbons mark the associated 95% credible intervals. Purple is used for quantities pertaining to all age classes, while black indicates adult females and pink indicates yearling females.

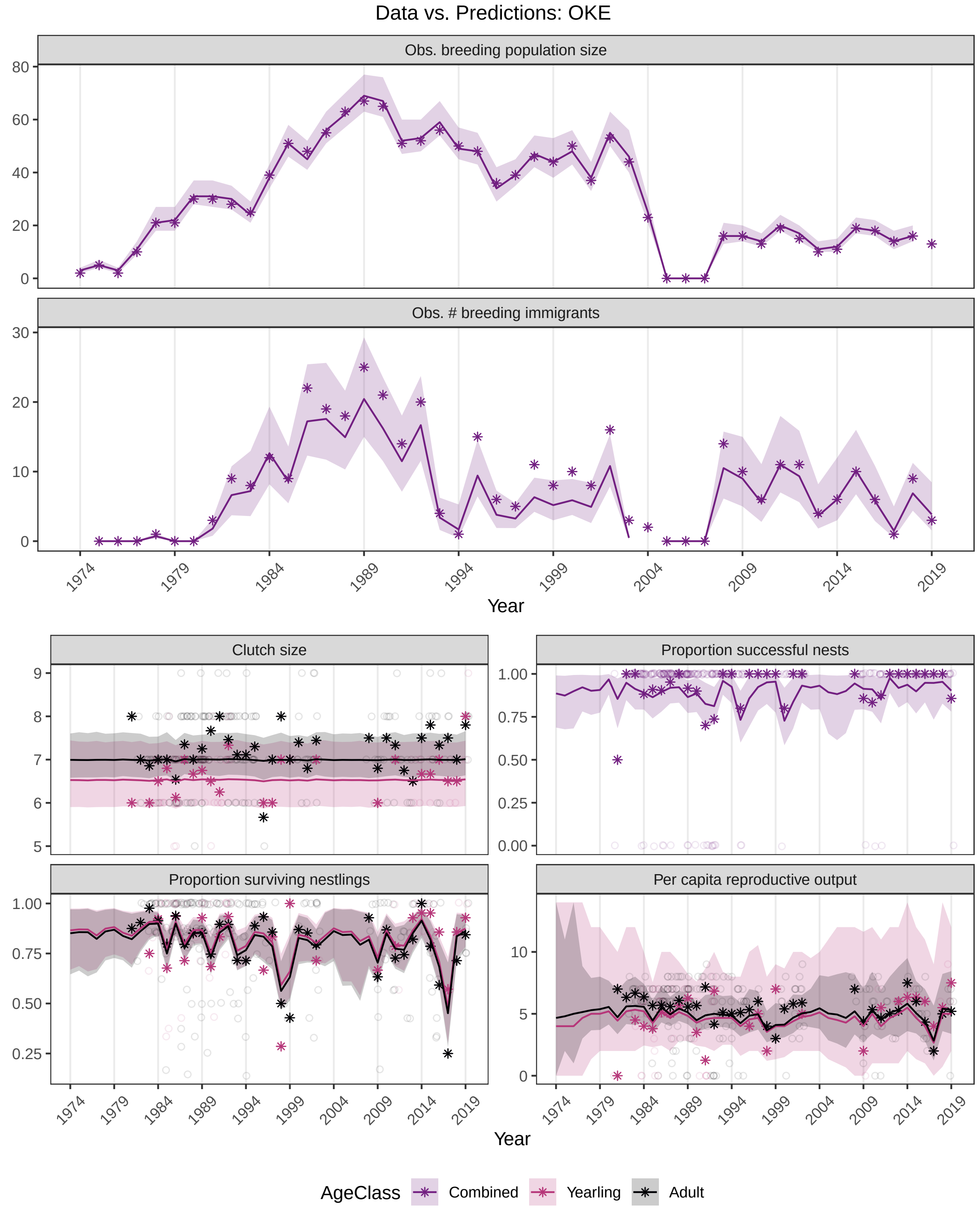

Figure S2.3: Comparison of model predictions to observational data for a variety of quantities for population OKE. Stars represent data values, solid lines are posterior median estimates, ribbons mark the associated 95% credible intervals. Purple is used for quantities pertaining to all age classes, while black indicates adult females and pink indicates yearling females. Note predictions of the number of breeding immigrants (second panel from the top) were not plotted in years in which the proportion of immigrant birds captured was unknown a priori and needed to be estimated within the IPM.

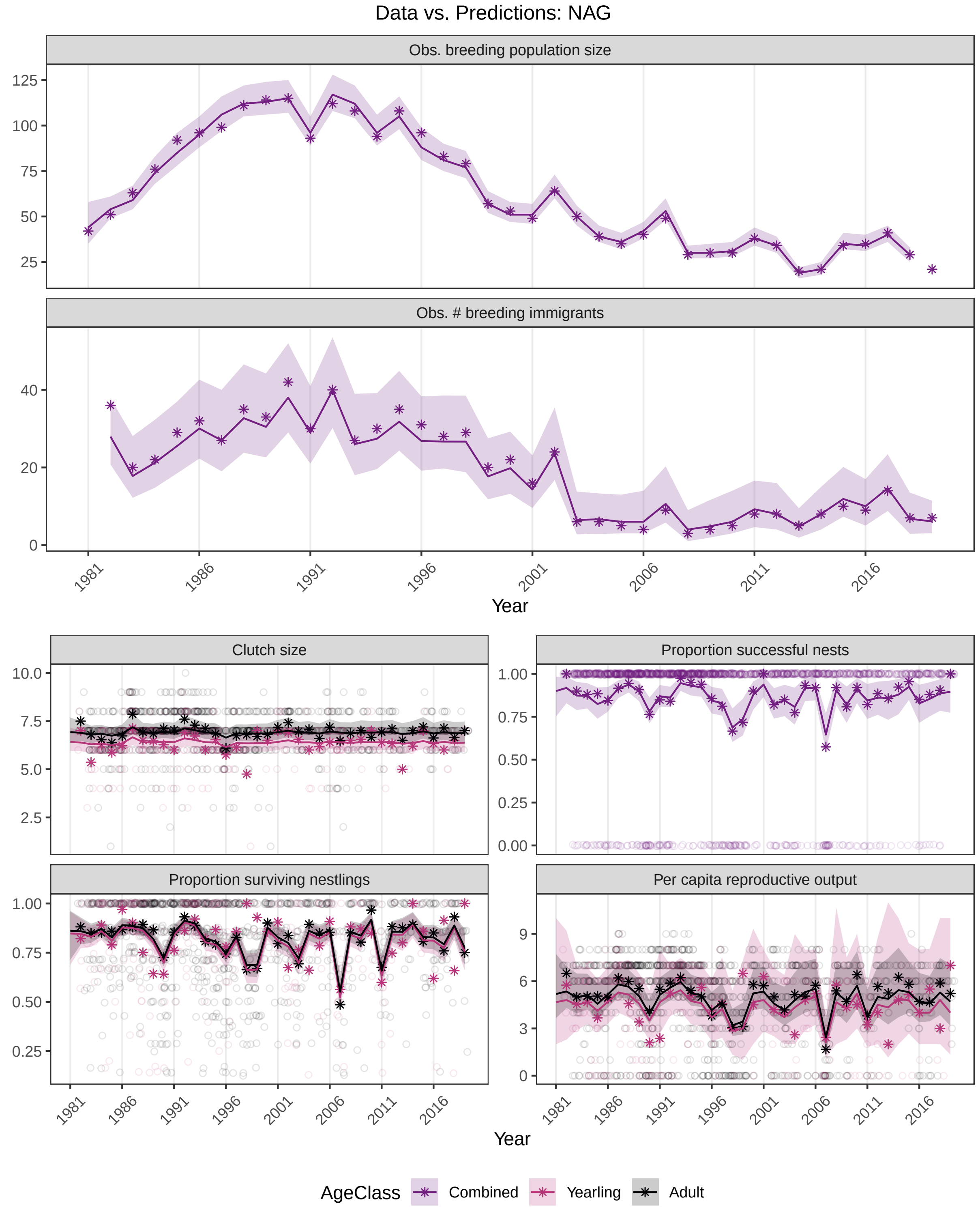

Figure S2.4: Comparison of model predictions to observational data for a variety of quantities for population NAG. Stars represent data values, solid lines are posterior median estimates, ribbons mark the associated 95% credible intervals. Purple is used for quantities pertaining to all age classes, while black indicates adult females and pink indicates yearling females.

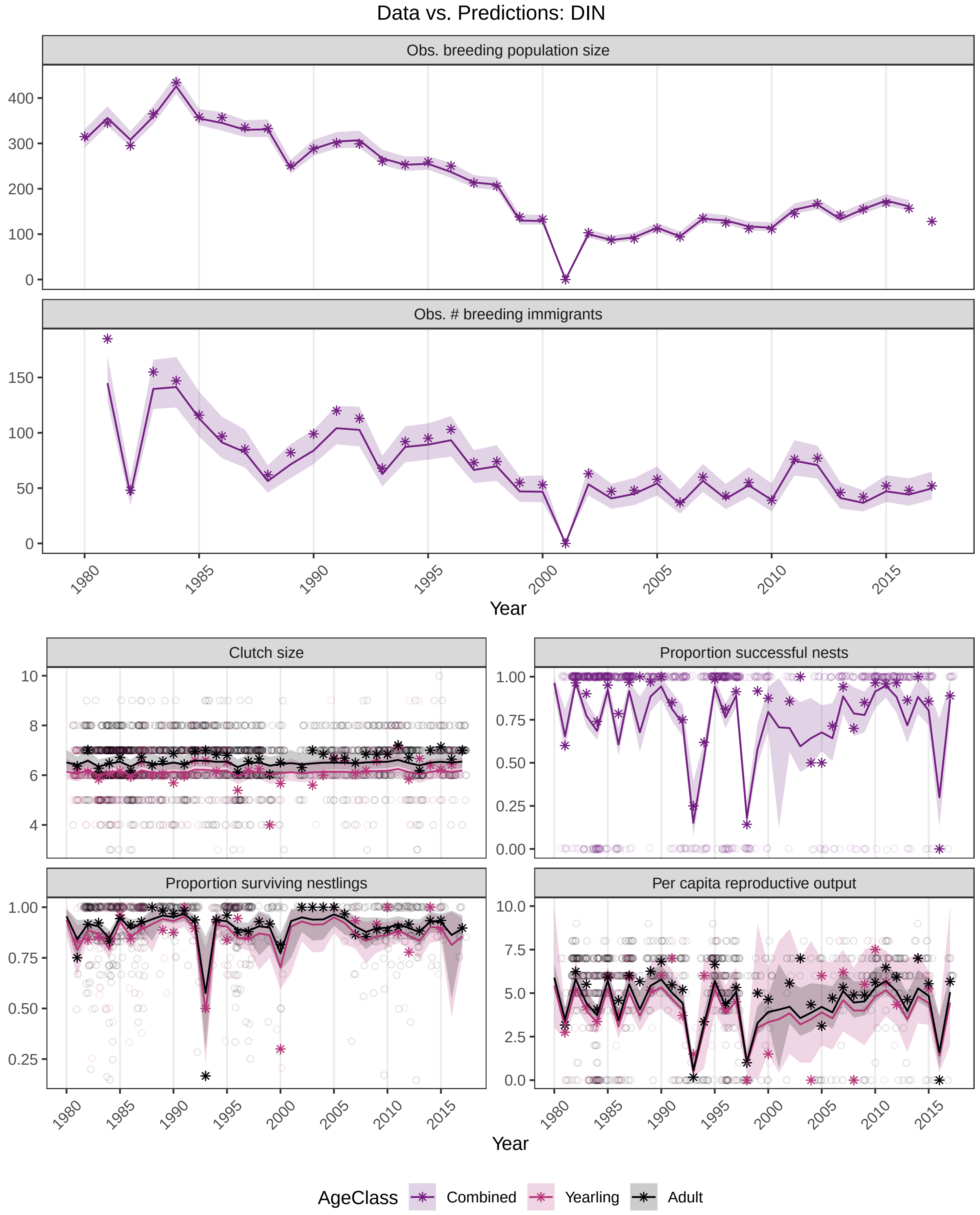

Figure S2.5: Comparison of model predictions to observational data for a variety of quantities for population DIN. Stars represent data values, solid lines are posterior median estimates, ribbons mark the associated 95% credible intervals. Purple is used for quantities pertaining to all age classes, while black indicates adult females and pink indicates yearling females.

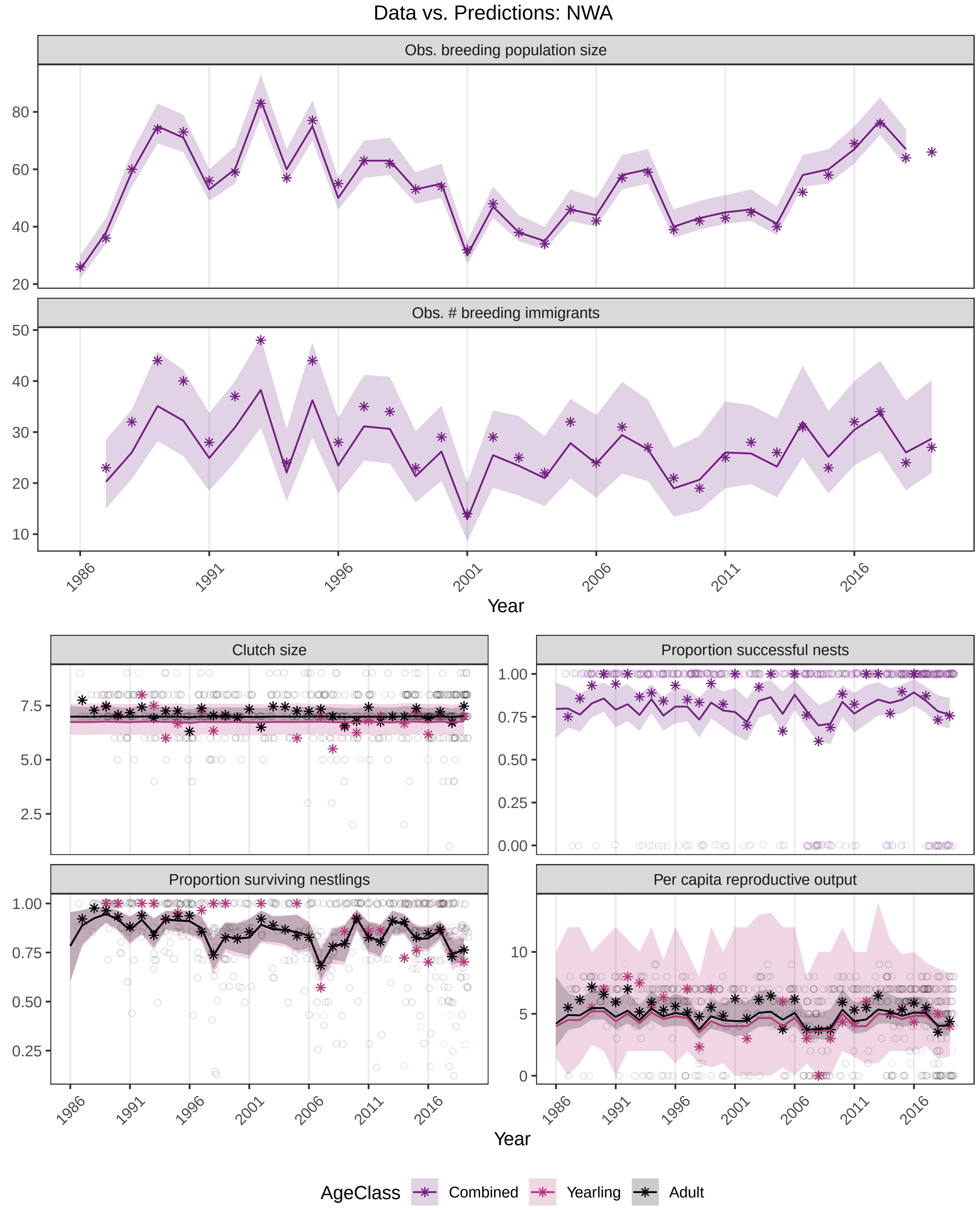

Figure S2.6: Comparison of model predictions to observational data for a variety of quantities for population NWA. Stars represent data values, solid lines are posterior median estimates, ribbons mark the associated 95% credible intervals. Purple is used for quantities pertaining to all age classes, while black indicates adult females and pink indicates yearling females.

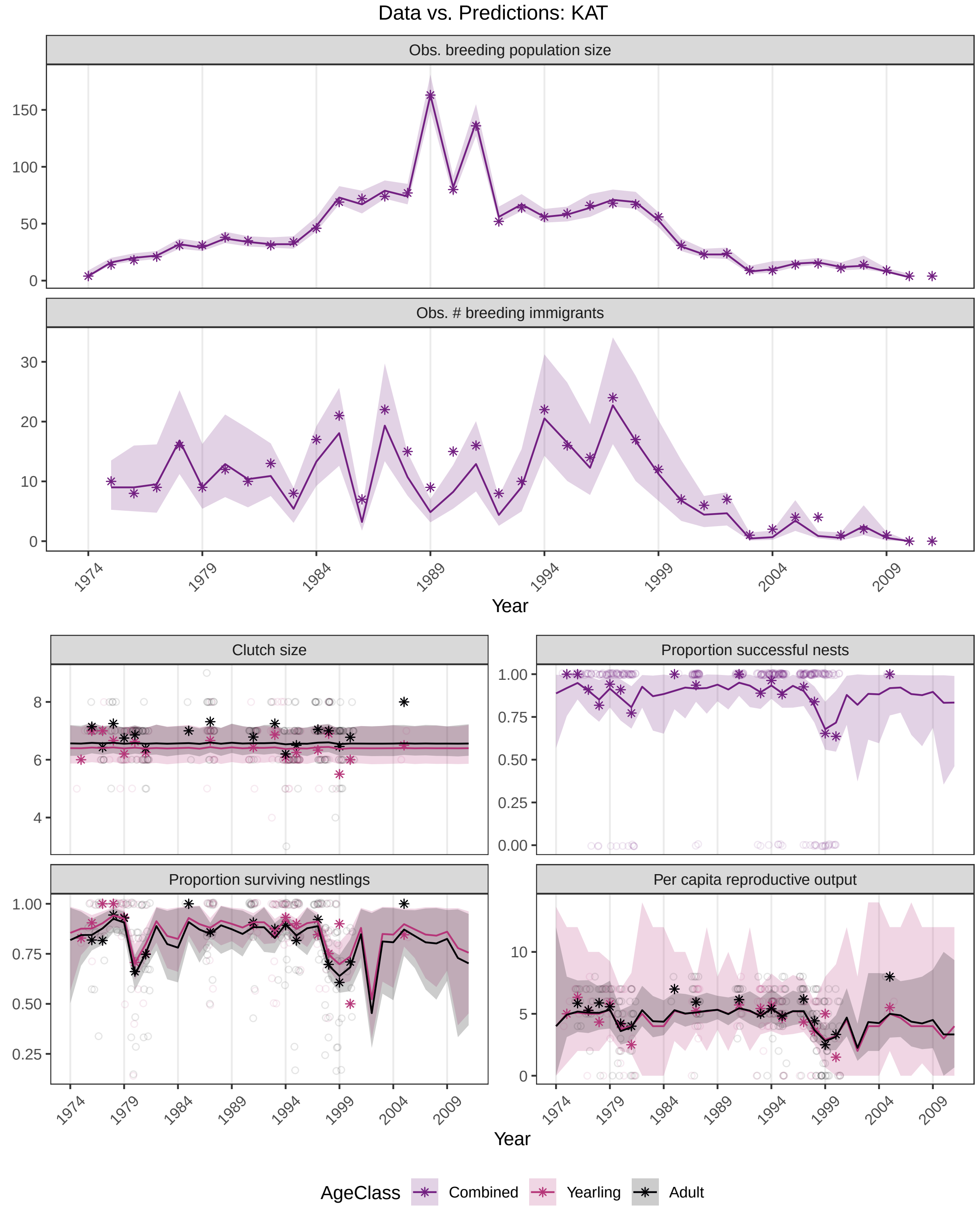

Figure S2.7: Comparison of model predictions to observational data for a variety of quantities for population KAT. Stars represent data values, solid lines are posterior median estimates, ribbons mark the associated 95% credible intervals. Purple is used for quantities pertaining to all age classes, while black indicates adult females and pink indicates yearling females.

S2.2 Comparing estimates from integrated vs. independent analyses

To check for major discrepancies among datasets, and between datasets and the population model, we re-ran the different vital rate models independently, i.e. without linkage among each other or to the population model. We subsequently compared posterior distributions of average age-specific vital rates (μ/`Mu` parameters) and standard deviations of among-year variation in vital rates (σ/`sigma` parameters) from the independent analyses to those from the integrated analyses.

The results are visualized in Figures S2.8-S2.14. For the majority of parameters, overlap of posterior distributions from independent and integrated models was very high, and for all study populations, integrated analysis increased precision of estimates of among-year variation in vital rates (σ/`sigma` parameters). For population KAT, the population with the most sparse data, improvements in parameter precision under integrated analysis were also visible for some vital rate averages (μ/`Mu` parameters, Figure S2.14). What all populations had in common was that integrated analyses estimated lower average adult annual survival (μ^{sA}/`Mu.sA`) than independent analyses. Several populations also had somewhat lower estimates of adult clutch size and nest success probabilities when data was analysed with an integrated model.

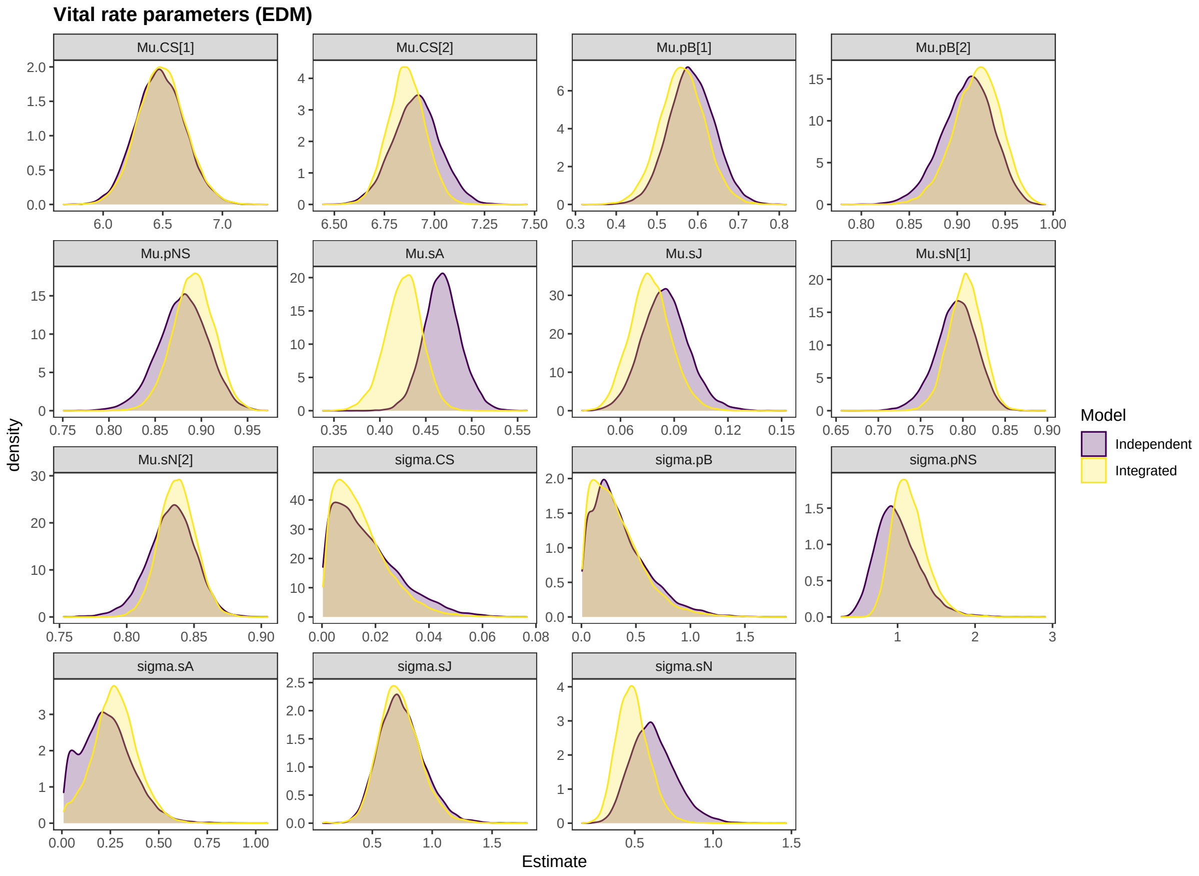

Figure S2.8: Posterior distributions of average age-specific vital rates (μ/`Mu` parameters) and standard deviations of among-year variation in vital rates (σ/`sigma` parameters) for population EDM obtained from independent (purple) and integrated (yellow) analyses.

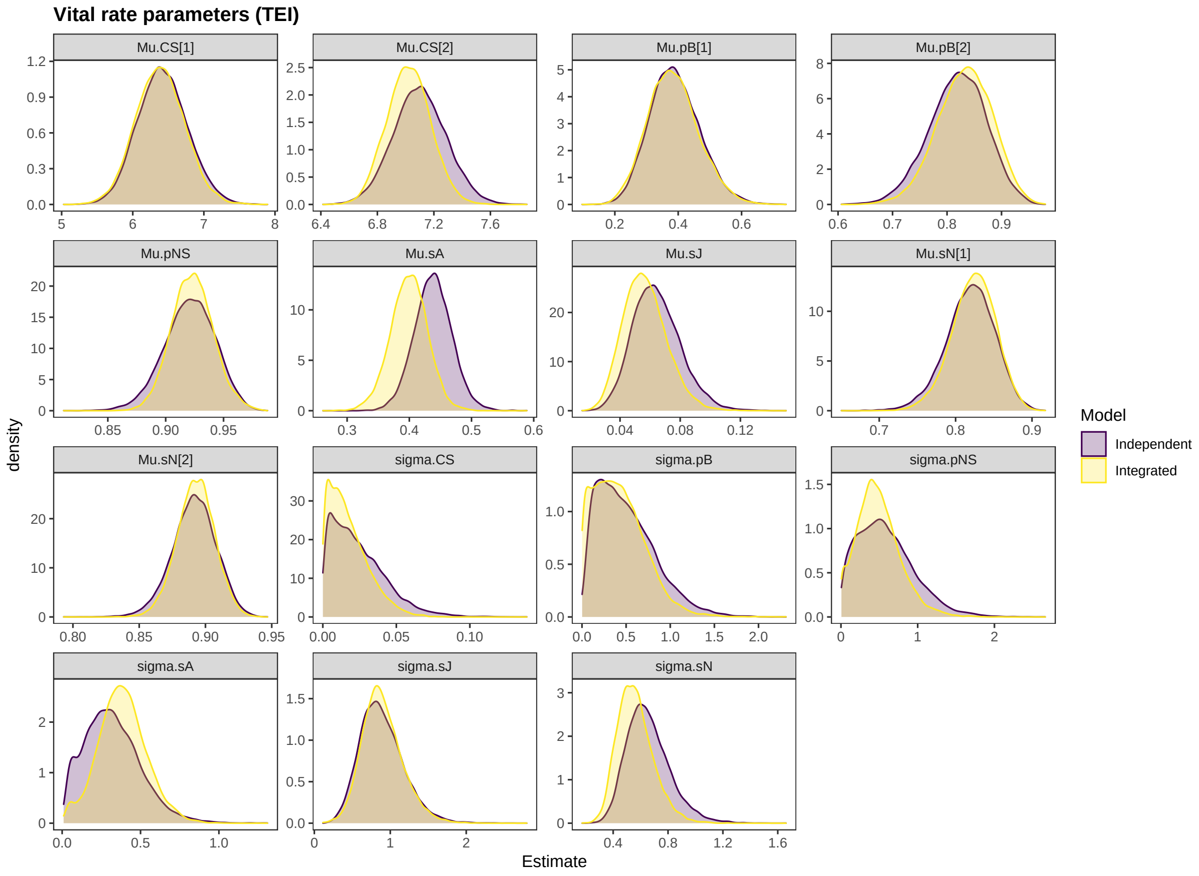

Figure S2.9: Posterior distributions of average age-specific vital rates (μ/Mu parameters) and standard deviations of among-year variation in vital rates (σ/sigma parameters) for population TEI obtained from independent (purple) and integrated (yellow) analyses.

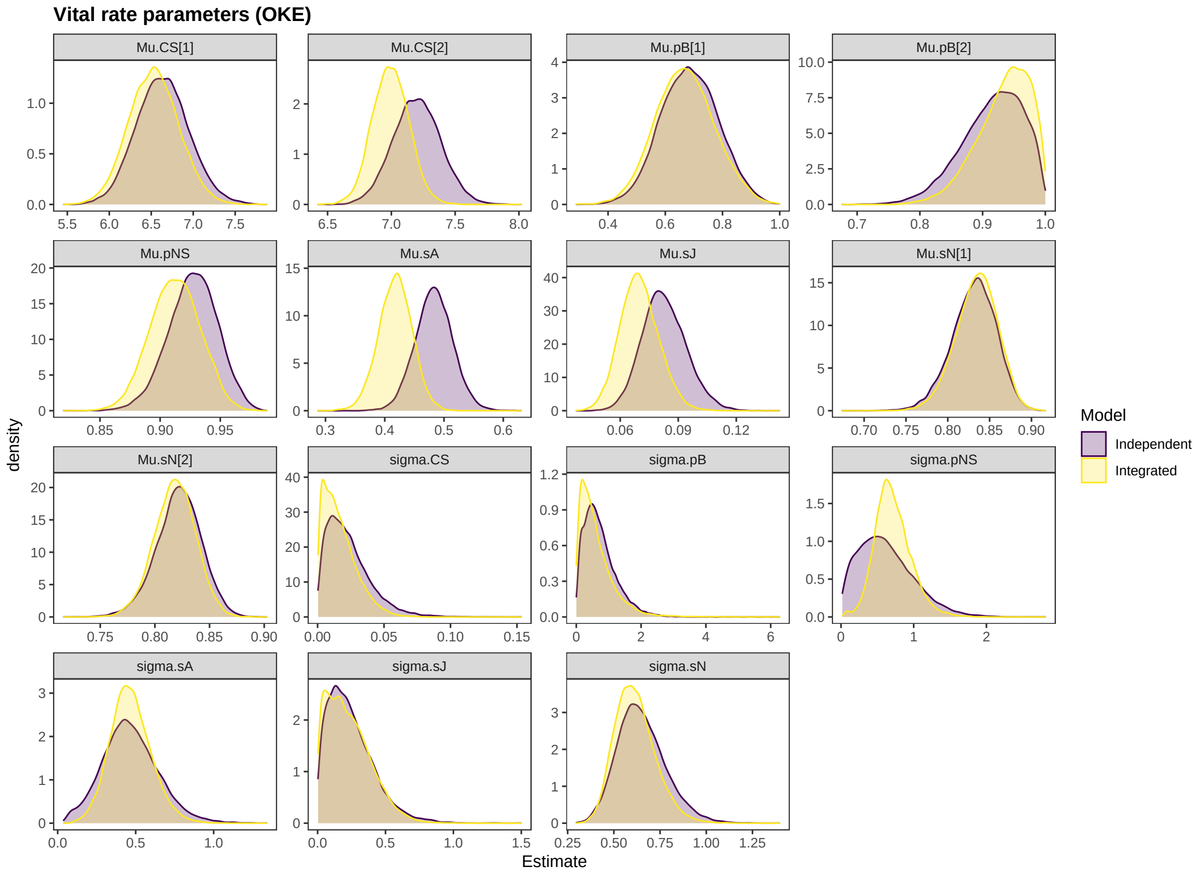

Figure S2.10: Posterior distributions of average age-specific vital rates (μ/Mu parameters) and standard deviations of among-year variation in vital rates (σ/sigma parameters) for population OKE obtained from independent (purple) and integrated (yellow) analyses.

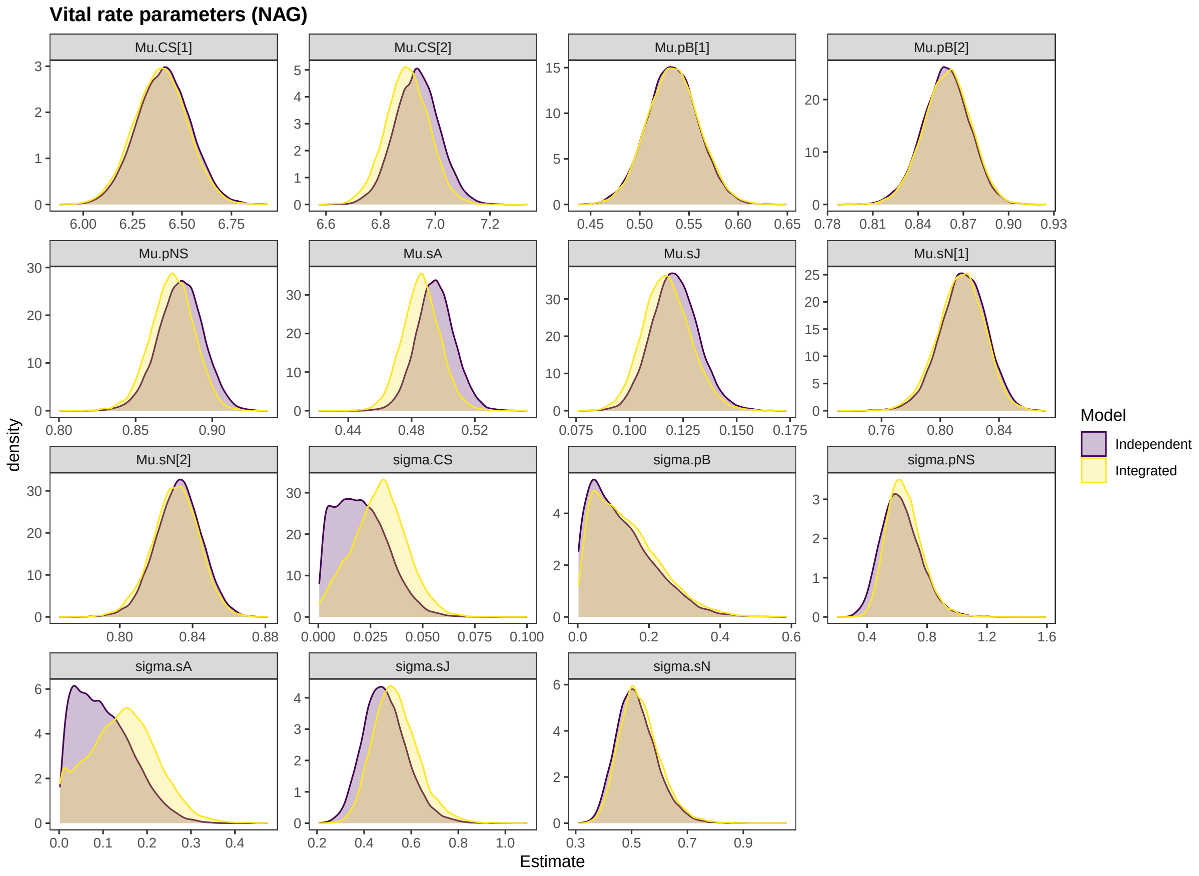

Figure S2.11: Posterior distributions of average age-specific vital rates (μ/`Mu` parameters) and standard deviations of among-year variation in vital rates (σ/`sigma` parameters) for population NAG obtained from independent (purple) and integrated (yellow) analyses.

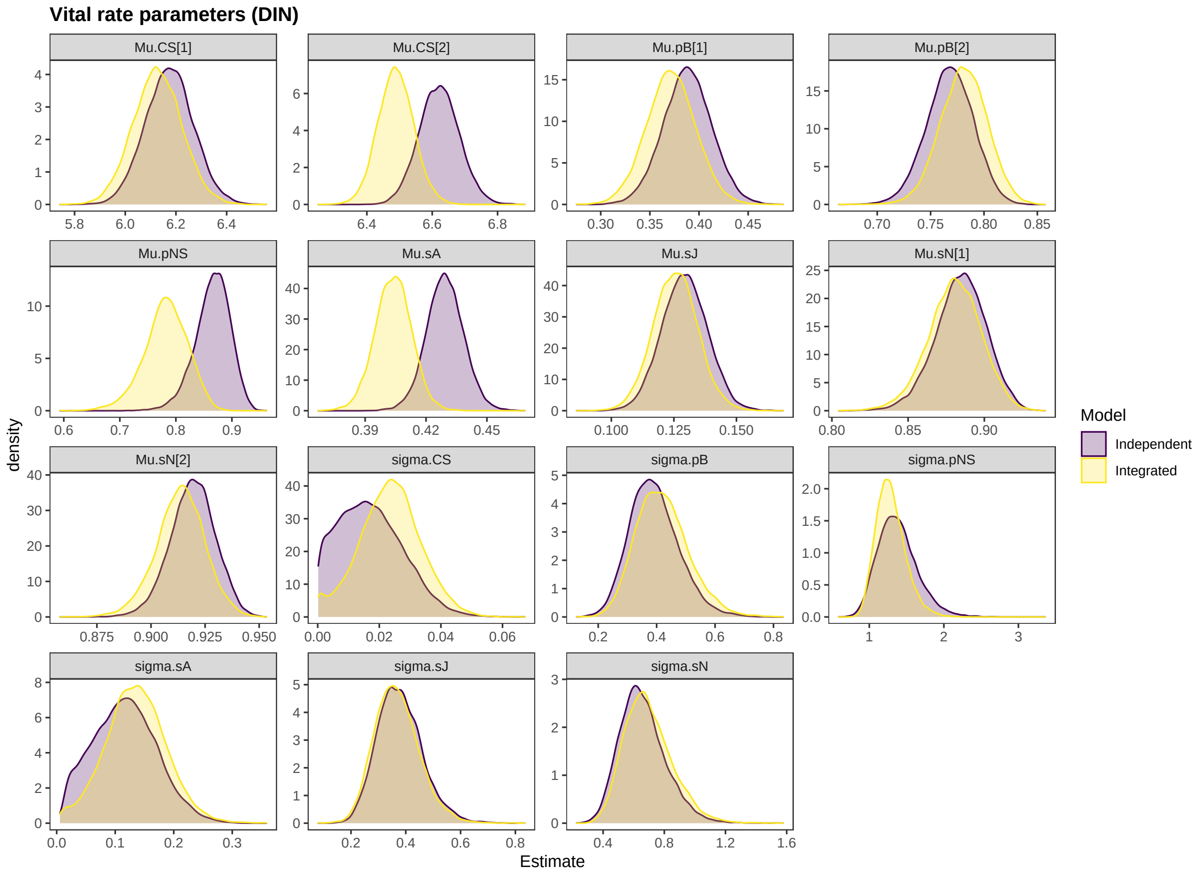

Figure S2.12: Posterior distributions of average age-specific vital rates (μ/`Mu` parameters) and standard deviations of among-year variation in vital rates (σ/`sigma` parameters) for population DIN obtained from independent (purple) and integrated (yellow) analyses.

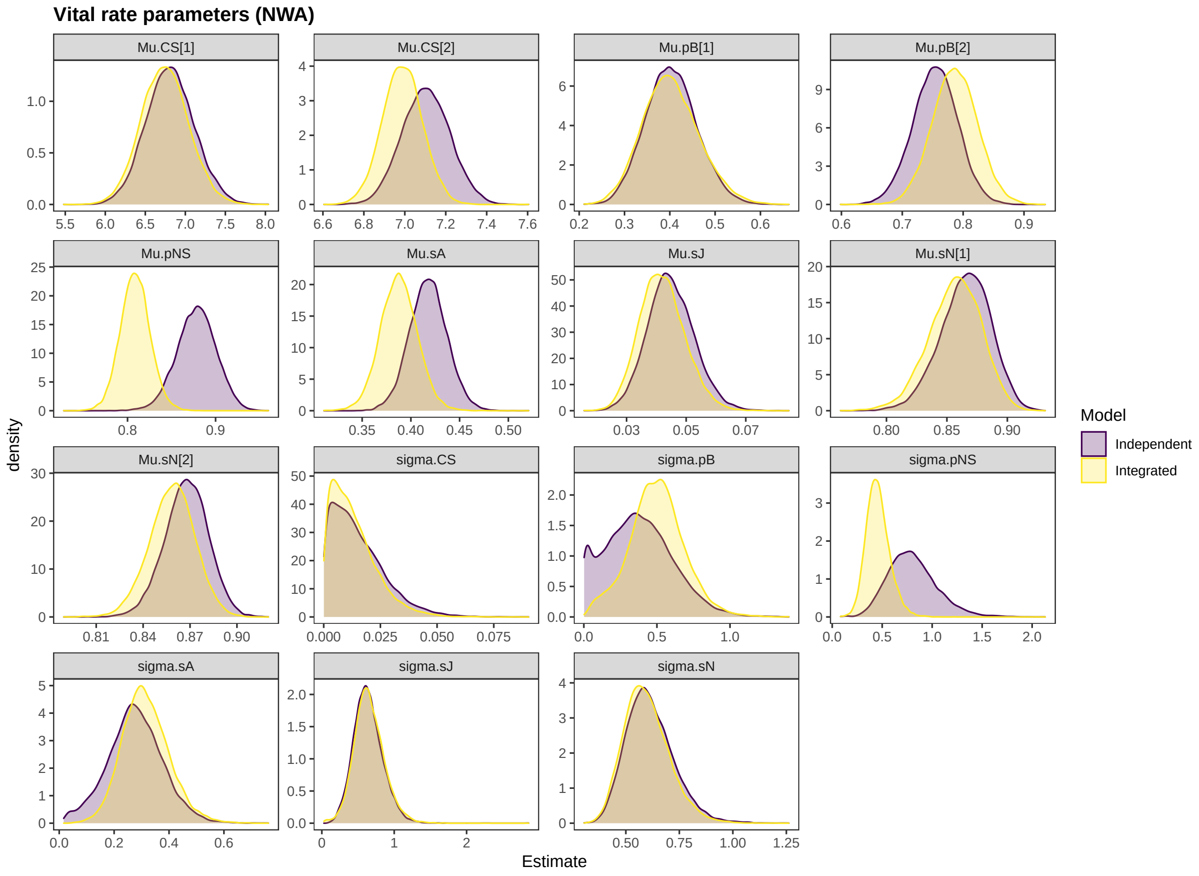

Figure S2.13: Posterior distributions of average age-specific vital rates (μ/`Mu` parameters) and standard deviations of among-year variation in vital rates (σ/`sigma` parameters) for population NWA obtained from independent (purple) and integrated (yellow) analyses.

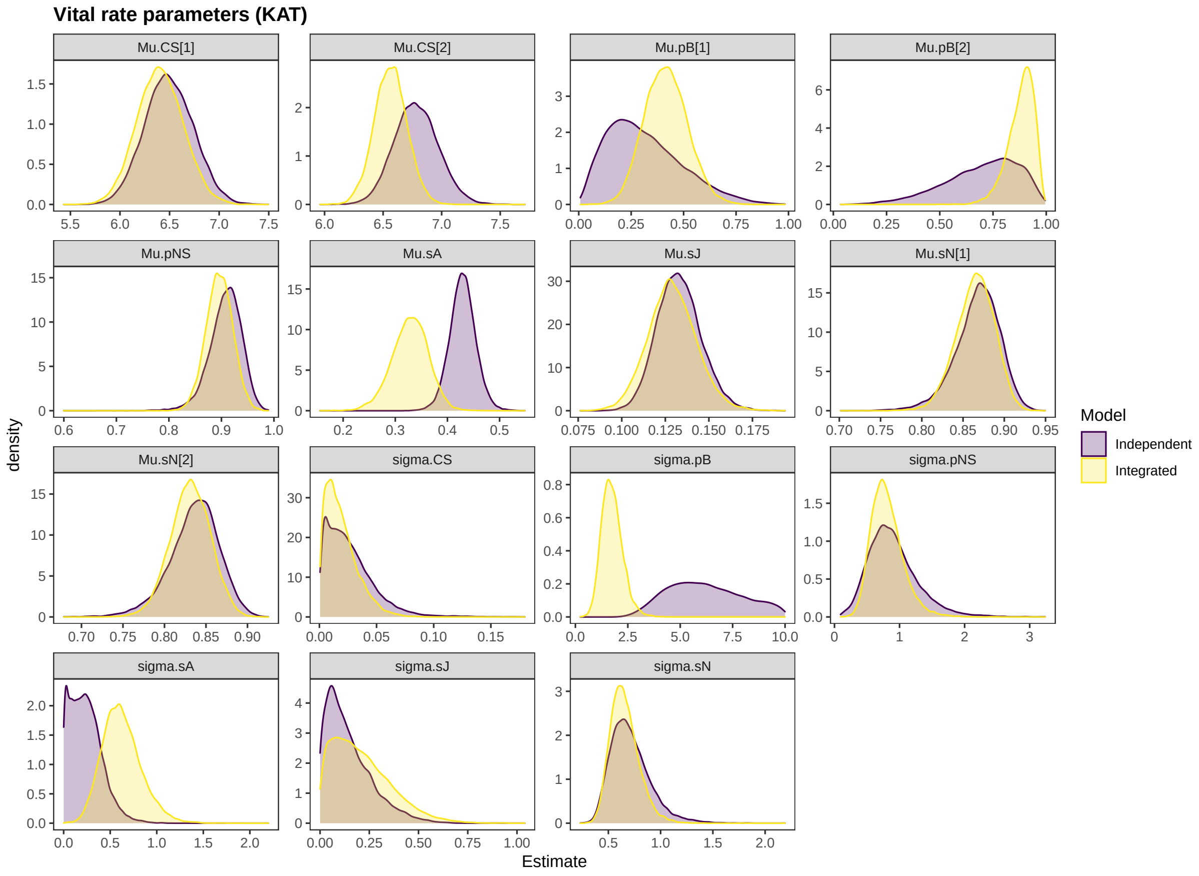

Figure S2.14: Posterior distributions of average age-specific vital rates (μ/Mu parameters) and standard deviations of among-year variation in vital rates (σ/sigma parameters) for population KAT obtained from independent (purple) and integrated (yellow) analyses.

S2.3 Projecting stochastic population dynamics

To verify that our model was able to make realistic projections of population dynamics, we ran stochastic matrix projections based on the posterior median estimates of age- and time-specific vital rates and immigrant numbers. For each population, we first assembled the projection matrix for each year within the study period:

$$T_t = \begin{bmatrix} 0.5pB_{Y,t}CS_{Y,t}pNS_tsN_{Y,t}sJ_t & 0.5pB_{Y,t}CS_{Y,t}pNS_tsN_{Y,t}sJ_t \\ sA_t & sA_t \end{bmatrix}$$

Next, we randomly sampled 200 sequences of 100 year indices t each, where each year index had the same chance of being sampled, the same year index could be included in the sample several times. We then and proceeded to project population trajectories using the population projection matrices and estimated median immigrant numbers corresponding to each year index within the sequence using matrix projection:

$$N_{t+1} = T_tN_t + Imm_{t+1}$$

As starting population size, we used the posterior median estimates of the number of yearling and adult females for the year corresponding to the first index t in the projection sequence.

For all populations, stochastic projections over 200 time-steps produced realistic population dynamics with regards to both population size and population structure. Dynamics were predicted to be relatively stable and without unrealistically large population increases or extinctions.

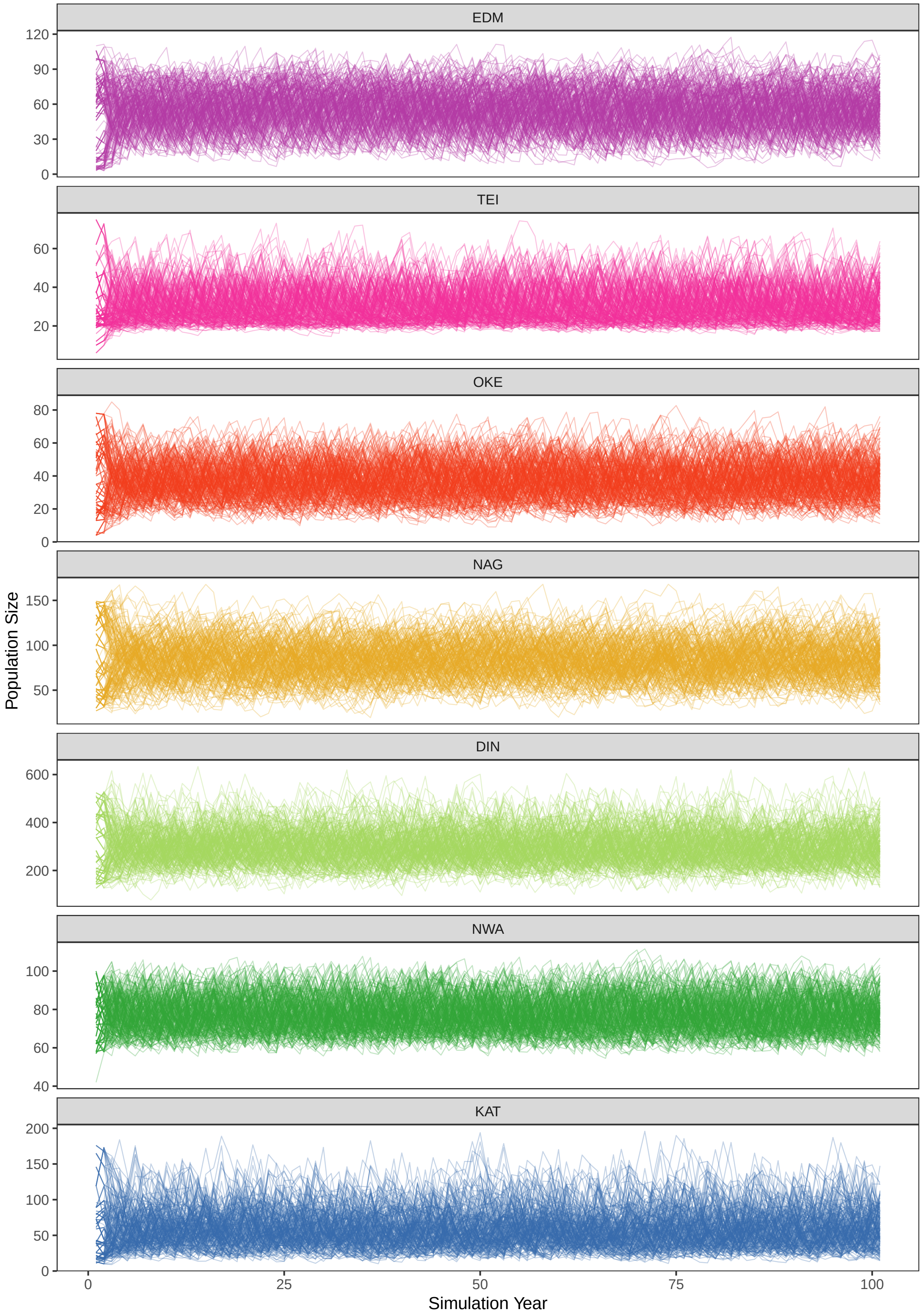

Figure S2.15: Population size trajectories for each study population predicted based on stochastic projections using matrices parameterized with posterior median estimates for vital rates and immigrant numbers. Each line represents one of 200 projected trajectories.

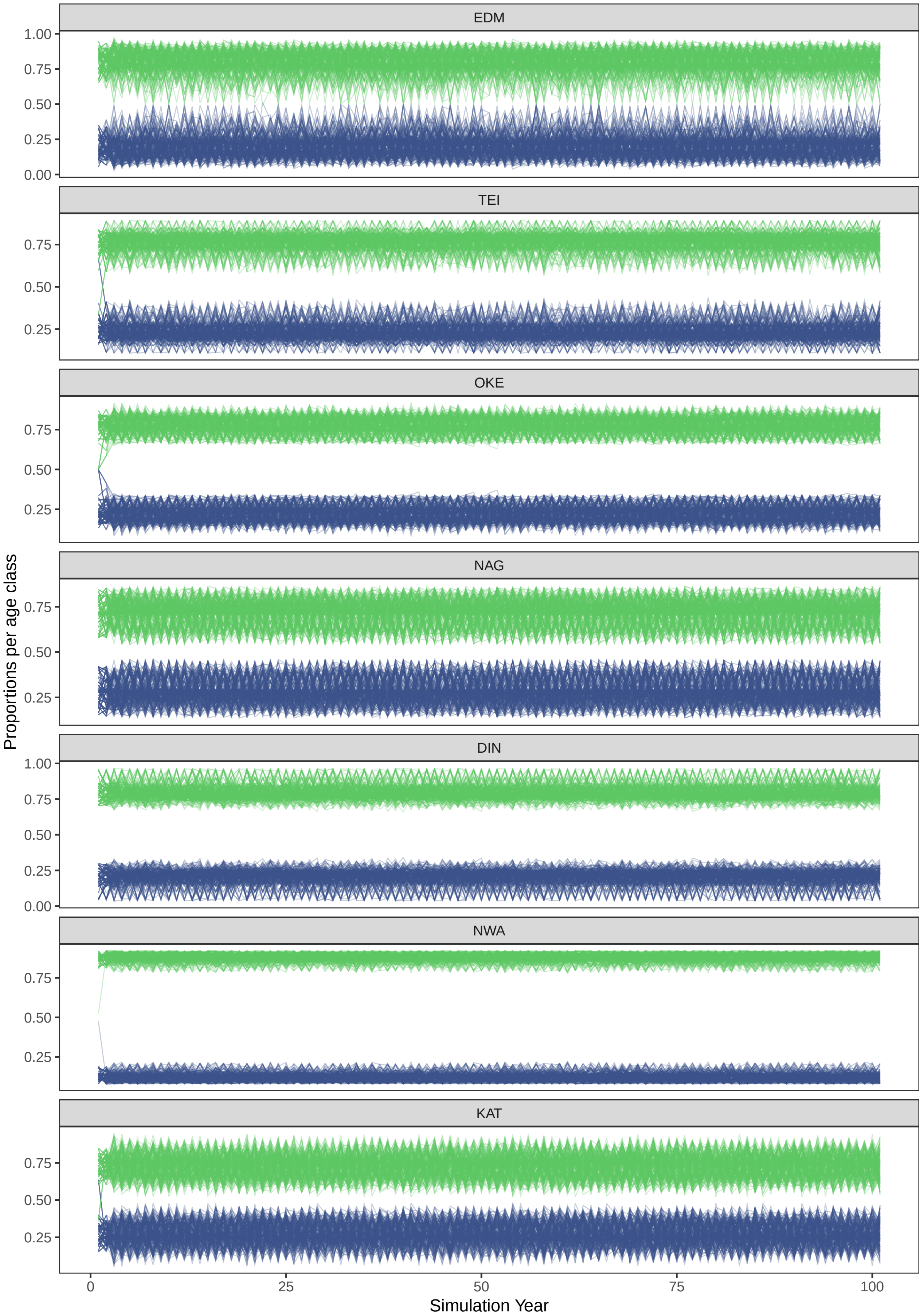

Figure S2.16: Population structure (as proportion yearlings in blue and proportion adults in green) trajectories for each study population predicted based on stochastic projections using matrices parameterized with posterior median estimates for vital rates and immigrant numbers. Each line represents one of 200 projected trajectories.

S3 Sensitivity derivation for random- and fixed-design LTRE

Realized population growth rate over time interval from t to $t+1$ (λ_t) can be described using matrix model notation as:

$$\lambda_t = \frac{N_{tot,t+1}}{N_{tot,t}} = \frac{T_t N_{,t} + Imm_{,t+1}}{N_{tot,t}} \tag{S3.1}$$

Here, $N_{tot,t}$ and $N_{,t}$ represent total and age-structured population size at census in year t, respectively, and $Imm_{,t}$ the number of immigrants of each age class entering the population prior to census in year t. T_t is the transition matrix for the two age classes (yearlings Y and adults A) from one census to the next:

$$T_t = \begin{bmatrix} F_{Y,t}sJ_t & F_{A,t}sJ_t \\ sA_t & sA_t \end{bmatrix} = \begin{bmatrix} 0.5pB_{Y,t}CS_{Y,t}pNS_t sN_{Y,t}sJ_t & 0.5pB_{A,t}CS_{A,t}pNS_t sN_{A,t}sJ_t \\ sA_t & sA_t \end{bmatrix} \tag{S3.2}$$

Equation S3.1 can therefore be rewritten in terms of absolute populations sizes as:

$$\lambda_t = \frac{F_{Y,t}sJ_tN_{Y,t} + sA_tN_{Y,t} + F_{A,t}sJ_tN_{A,t} + sA_tN_{A,t} + Imm_{Y,t+1} + Imm_{A,t+1}}{N_{Y,t} + N_{A,t}} \tag{S3.3}$$

and in terms of proportional population sizes as

$$\lambda_t = F_{Y,t}sJ_tn_{Y,t} + sA_tn_{Y,t} + F_{A,t}sJ_tn_{A,t} + sA_tn_{A,t} + imm_{Y,t+1} + imm_{A,t+1} \tag{S3.4}$$

Here, $n_{a,t} = \frac{N_{a,t}}{N_{Y,t}+N_{A,t}}$ and $imm_{a,t} = \frac{Imm_{a,t}}{N_{Y,t}+N_{A,t}}$. Using equation and S3.4, we calculate sensitivity (s) of λ_t to small changes in any parameter θ_t as the first-order derivative: $s_\theta = \frac{\delta\lambda_t}{\delta\theta_t}$.

S3.1 Sensitivities with regards to vital rates

$$\frac{\delta\lambda_t}{\delta sJ_t} = F_{Y,t}n_{Y,t} + F_{A,t}n_{A,t} = 0.5pB_{Y,t}CS_{Y,t}pNS_tsN_{Y,t}n_{Y,t} + 0.5pB_{A,t}CS_{A,t}pNS_tsN_{A,t}n_{A,t} \quad \text{(S3.5)}$$

$$\frac{\delta\lambda_t}{\delta sA_t} = n_{Y,t} + n_{A,t} = 1 \quad \text{(S3.6)}$$

$$\frac{\delta\lambda_t}{\delta pB_{a,t}} = 0.5CS_{a,t}pNS_tsN_{a,t}sJ_tn_{a,t} \quad \text{(S3.7)}$$

$$\frac{\delta\lambda_t}{\delta CS_{a,t}} = 0.5pB_{a,t}pNS_tsN_{a,t}sJ_tn_{a,t} \quad \text{(S3.8)}$$

$$\frac{\delta\lambda_t}{\delta pNS_t} = 0.5pB_{Y,t}CS_{Y,t}sN_{Y,t}n_{Y,t} + 0.5pB_{A,t}CS_{A,t}sN_{A,t}n_{A,t} \quad \text{(S3.9)}$$

$$\frac{\delta\lambda_t}{\delta sN_{a,t}} = 0.5pB_{a,t}CS_{a,t}pNS_tsJ_tn_{a,t} \quad \text{(S3.10)}$$

S3.2 Sensitivities with regards to population structure

$$\frac{\delta\lambda_t}{\delta n_{Y,t}} = sJ_t(F_{Y,t} - F_{A,t}) = sJ_tpNS_t(0.5pB_{Y,t}CS_{Y,t}sN_{Y,t} - 0.5pB_{A,t}CS_{A,t}sN_{A,t}) \quad \text{(S3.11)}$$

$$\frac{\delta\lambda_t}{\delta n_{A,t}} = sJ_t(F_{A,t} - F_{Y,t}) = sJ_tpNS_t(0.5pB_{A,t}CS_{A,t}sN_{A,t} - 0.5pB_{Y,t}CS_{Y,t}sN_{Y,t}) \quad \text{(S3.12)}$$

$$\frac{\delta\lambda_t}{\delta imm_{a,t+1}} = 1 \quad \text{(S3.13)}$$

S4 Period-design LTRE with immigration

The following sections provide some additional information on the transient "period-design" LTRE derived by Koons *et al.* (2016) and our extension of it to include immigration (= open populations). For additional background and more in-depth details of the method, we refer the reader to Koons *et al.* (2016) and Koons *et al.* (2017). We also note that this particular analysis becomes much more accessible when working with code in addition to the theoretical equations only. Our code for implementing an open-population period-design LTRE for `SPI-IPM` is available on GitHub: `https://github.com/SPI-Birds/SPI-IPM/tree/main/SPI-IPM_Code/07_Follow-up_Analyses/LTRE_PeriodDesign`.

S4.1 Calculating real-time elasticities

Performing a period-design LTRE requires calculation of four types of real-time elasticities:

- $e^T_{\mu_{i,t}}$: direct effects of changes in vital rate means
- $e^T_{\sigma_{i,t}}$: direct effects of changes in vital rate standard deviations
- $e^{n_t}_{\mu_{i,t}}$ indirect effects of changes in vital rate means (via population structure)
- $e^{n_t}_{\sigma_{i,t}}$ indirect effects of changes in vital rate standard deviations (via population structure)

Real-time elasticities are derived as introduced by Haridas *et al.* (2009), and Koons *et al.* (2016) provided alternative notation that is more familiar to ecologists. Below, we largely adopt Koons' notation with the exception that we designate the population projection matrix as T instead of A (to avoid confusion with our use of A as a subscript indicating the adult age class), and use a instead of k to index age for consistency with the rest of the article.

Derivation of the direct-effect elasticities, $e^T_{\mu_{i,t}}$ and $e^T_{\sigma_{i,t}}$ is relatively straightforward:

$$e^T_{\mu_{i,t}} = \sum_j \sum_a \frac{\mu_i \times \frac{\delta T_{jia,t}}{\delta \theta_{i,t}} \times n_{a,t}}{\lambda_t} \tag{S4.1}$$

$$e^T_{\sigma_{i,t}} = \sum_j \sum_a \frac{(\theta_{i,t} - \mu_i) \times \frac{\delta T_{jia,t}}{\delta \theta_{i,t}} \times n_{a,t}}{\lambda_t} \tag{S4.2}$$

Here, $\theta_{i,t}$ and μ_i are the time-specific value and time-period-average of vital rate i, respectively. $T_{ija,t}$ represents the elements of the population projection matrix (see Equation S4.8), $n_{a,t}$ the proportion of

the population in class a, and λ_t the realized population growth rate from year t to $t+1$.

Note that the term $\frac{\delta T_{jia,t}}{\delta \theta_{i,t}} \times n_{a,t}$ is essentially equivalent to the transient sensitivity on a per-matrix-element basis, and that the equations therefore take on the classical form of an elasticity equation: $e_{\theta_i} = \frac{\theta_i}{\lambda_t} \times s_{\theta_i}$ where s_{θ_i} is the sensitivity (Caswell 2001).

The calculation of the indirect-effect real-time elasticities, $e^{n_t}_{\mu_{i,t}}$ and $e^{n_t}_{\sigma_{i,t}}$, is more complex and easily the most challenging part of the period-design LTRE. Following Haridas *et al.* (2009):

$$e^{n_t}_{\mu_{i,t}} = \sum_j \sum_a \frac{T_{jia,t} \times w_{a,t}}{\lambda_t} \tag{S4.3}$$

where $w_{a,t}$ summarises the cumulative effect of changes in $\mu_{i,t}$ (or $\theta_{i,t} - \mu_{i,t}$ when calculating $e^{n_t}_{\sigma_{i,t}}$) via perturbation of population structure. w is obtained via a recursive calculation:

$$w_{t+1} = \frac{(I - n_{t+1}e')(C_t n_t + T_t w_t)}{\lambda_t} \tag{S4.4}$$

I is an identity matrix and e a vector of 1's (both of them are necessary to ensure correct dimensionality). C_t is the perturbation matrix; in practice, the term $C_t \times n_t$ contains sensitivities and – on a per-vital-rate-basis – corresponds to either $\mu_i \times \frac{\delta T_{jia,t}}{\delta \theta_{i,t} \times n_t}$ or $(\theta_{i,t} - \mu_i) \times \frac{\delta T_{jia,t}}{\delta \theta_{i,t} \times n_t}$ (depending on which real-time elasticity is calculated). w_t starts at a value of 0 at $t = 0$.

The population simulation underlying the calculation of real-time elasticities is done for a hypothetical reference population (Koons *et al.* 2016). This reference population is defined to have vital rates and population structure corresponding to the average of the two time-periods being compared in the period-design LTRE. All time-dependent vital rates $\theta_{i,t}$ are thus calculated as the mean of θ_i at the t-th time-step of the first time-period and θ_i at the t-th time-step of the second time-period. The same applies for the population proportions ($n_{a,t}$) used in the calculation of direct-effect real-time elasticities. Time-average vital rates (μ_i) are defined as the means of vital rate averages for the first and second time-period. The population growth rates (λ_t) using in the elasticity calculations, on the other hand, are extracted from the simulation.

S4.2 Extension to open populations

The period-design LTRE developed by Koons *et al.* (2016) assumes a closed population, i.e. no immigration into the population. Since the pied flycatcher IPMs used in this study do include immigration, we extended the period-design LTRE to take into account immigration (rates) of both yearling and adult individuals. This required deriving direct- and indirect-effect real-time elasticities for changes in mean and variation of immigration rates between time-periods. Notably, the extension to open populations does not affect the real-time elasticities of any of the local vital rates since the immigration rates in the present models are assumed to be insensitive to changes in local vital rates and population structure.

We derived the direct-effect real-time elasticities for immigration rates, $e^{imm_k}_{\mu_{i,t}}$ and $e^{imm_k}_{\sigma_{i,t}}$ both analytically following Haridas *et al.* (2009) and by substituting transient sensitivity into equations S4.1 and S4.2, and arrived at the same solution of:

$$e^{imm_a}_{\mu_{i,t}} = \frac{\mu_i}{\lambda_t} = \frac{\mu_{imm_a}}{\lambda_t} \tag{S4.5}$$

and

$$e^{imm_a}_{\mu_{i,t}} = \frac{(\theta_{i,t} - \mu_i)}{\lambda_t} = \frac{(imm_{a,t} - \mu_{imm_a})}{\lambda_t} \tag{S4.6}$$

A change in immigration rates at time t will affect population structure and thus also have indirect effects on population growth rates (at subsequent times $t + x$, where $x > 0$). These indirect effects are mediated by altered contributions of **local** vital rates as a consequence of population structure perturbation. There are no indirect effects altering contributions of immigration rates at future time-steps as immigration rates are assumed to be independent of local population structure. Hence, indirect-effect real-time elasticities for immigration rates can be calculated by substituting $C_t n_t$ (representing the current time-step) in equation S4.7 by μ_{imm_a} (or $imm_{a,t} - \mu_{imm_a}$) while leaving $T_t w_t$ (representing carry-over effects from earlier time-steps) as it is:

$$w_{t+1} = \frac{(I - n_{t+1}e')(\mu_{imm_a} + T_t w_t)}{\lambda_t} \tag{S4.7}$$

To verify that our derivations of real-time elasticities for immigration rates were indeed correct, we implemented a second period-design LTRE in which we re-parameterized the population such that yearling

and adult immigration rates were contained within the projection matrix $T_{alt,t}$:

$$T_{alt,t} = \begin{bmatrix} F_{Y,t}sJ_t + imm_{Y,t} & F_{A,t}sJ_t + imm_{Y,t} \\ sA_t + imm_{A,t} & sA_t + imm_{A,t} \end{bmatrix} \tag{S4.8}$$

Note that even though the immigration rates are within the projection matrix in this model, they are still functionally independent of local population structure. This model is therefore mathematically equivalent to our original model in which a number of immigrants gets added to the population "externally" at each projection step. Using this alternative model, we then ran a closed-population period-design LTRE by adapting the code from Koons *et al.* (2017) and compared the results to those obtained using our newly derived open population period-design LTRE.

Posterior distributions of LTRE contributions were largely identical for both approaches (Figure S4.1), confirming that our implementation of an open-population period-design LTRE is valid.

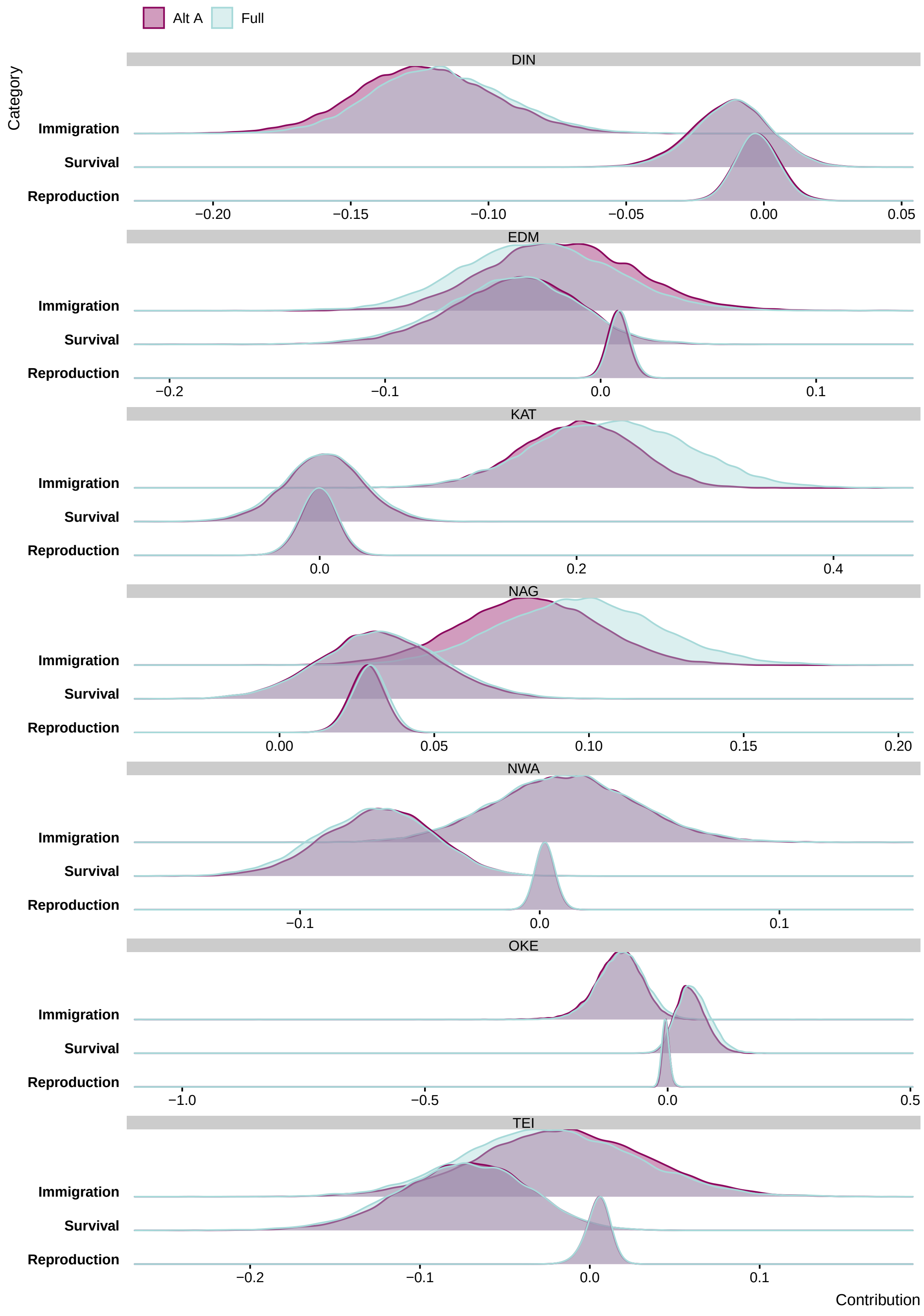

Figure S4.1: Posterior distributions for contributions of immigration, survival, and reproduction to changes in longer-term population-growth rate, calculated using a newly derived open-population period-design LTRE (light blue) and an established closed-population period-design LTRE with an alternative population model formulation (pink).

S4.3 Dealing with zeros in vital rate estimates

One practical issue we came across when implementing period-design LTREs for the pied flycatcher IPMs was that, depending on the population, time-period, and MCMC sample chosen, it could happen that μ_i and σ_i were 0 (the affected vital rate i was always immigration rate, typically for yearlings, imm_Y). Since the calculation of contributions in the period-design LTRE involves $log(\mu_i)$ and $log(\sigma_i)$ (see equation in section 2.6 of the main text), this resulted in the introduction of NA values in some of the LTRE contributions for the affected parameters. This generates "incomplete sets" of contributions, which can potentially lead to biased inference about the relative importance of different vital rates.

To circumvent this problem, we tested out 2 different approaches to handling samples with $\mu_i = \sigma_i = 0$:

1. Identify and remove entire affected MCMC samples (i.e. drop estimates for all parameters)
2. Identify all affected μ_i and σ_i and replace the 0 with the smallest estimated value > 0 (contained in any other MCMC sample for the same population)

The second approach was inspired by the recommendations of Warton & Hui (2011). We found that both approaches yielded nearly identical results, and implemented the first approach for obtaining the results presented in this article. We note, however, that the two approaches yielding identical results was likely due to the relatively rare occurrence of $\mu_i = \sigma_i = 0$ in our IPMs. While commonly occurring vital rate estimates of 0 across entire MCMC samples should be a warning by themselves, this may warrant additional caution when implementing period-design LTREs.

References

Caswell, H. (2001) *Matrix population models: construction, analysis, and interpretation.* Sunderland, Mass.: Sinauer Associates.

Haridas, C., Tuljapurkar, S. & Coulson, T. (2009) Estimating stochastic elasticities directly from longitudinal data. *Ecology letters* **12**, 806–812.

Koons, D.N., Arnold, T.W. & Schaub, M. (2017) Understanding the demographic drivers of realized population growth rates. *Ecological Applications* **27**, 2102–2115.

Koons, D.N., Iles, D.T., Schaub, M. & Caswell, H. (2016) A life-history perspective on the demographic drivers of structured population dynamics in changing environments. *Ecology letters* **19**, 1023–1031.

Warton, D.I. & Hui, F.K. (2011) The arcsine is asinine: the analysis of proportions in ecology. *Ecology* **92**, 3–10.
