# Supplementary material for: Spatial consistency in drivers of population dynamics of a declining migratory bird
Source: J Anim Ecol. 2022 Nov 18;92(1):97–111. doi: 10.1111/1365-2656.13834 (PMC10099983; doi:10.1111/1365-2656.13834)
Supplement: Supplementary file 1 — Appendix S1 [file JANE-92-97-s002.zip › JANE_13834_SPI-IPM_CodeManual_Ch2-4.pdf]

Chapter 2

IPM Construction

2.1 Open population model with 2 age classes

2.1.1 Model description

Population dynamics are represented using a female-based age-structured open population model with a pre-breeding census in spring. At census, females are divided into two age classes: "yearlings" (1-year old birds hatched during the breeding season of the previous year) and "adults" (birds older than one year). The motivation underlying this distinction is that reproductive output often differs for these two age classes in passerine birds. The dynamics of the female segment of the population over the time-interval from census in year t to census in year $t+1$ can be described with classic matrix notation (Caswell, 2001) as:

$$N_{tot,t+1} = \begin{bmatrix} N_{Y,t+1} \\ N_{A,t+1} \end{bmatrix} = \begin{bmatrix} 0.5F_{Y,t}sJ_t & 0.5F_{A,t}sJ_t \\ sA_t & sA_t \end{bmatrix} \begin{bmatrix} N_{Y,t} \\ N_{A,t} \end{bmatrix} + \begin{bmatrix} Imm_{Y,t+1} \\ Imm_{A,t+1} \end{bmatrix}$$

$N_{tot,t+1}$ represents the total number of yearling and adult females in the population upon arrival in the breeding areas in year t. The total female population size, $N_{tot,t+1}$, is the sum of the numbers of yearling and adult females in the population in year $t+1$ ($N_{Y,t+1}$ and $N_{A,t+1}$, respectively) and consists of local survivors and recruits from the previous breeding season, as well as immigrant yearling ($Imm_{Y,t+1}$) and adult ($Imm_{A,t+1}$) females.

$F_{a,t}$ represents the expected number of fledglings produced by age class a females during the breeding season in year t and is the product of several vital rates.

First, females in age class a may breed in a nestbox with probability $pB_{a,t}$ upon arrival to breeding areas in year t. Each breeding female may then lay a clutch containing a certain number of eggs (expected number = $CS_{a,t}$), and each egg within the clutch may hatch and survive to fledging. The probability of an egg hatching and surviving to fledging is divided into an age-independent probability of nest success (pNS_t, probability of complete clutch failure = $1 - pNS_t$) and a survival probability of every egg/chick to fledging provided that the nest has not failed entirely ($sN_{a,t}$, with a = age of the mother). Consequently, the expected number of fledglings produced by age class a females in year t is defined as:

$$F_{a,t} = pB_{a,t} \times CS_{a,t} \times pNS_t \times sN_{a,t} \tag{2.1}$$

Fledglings that survive to the next breeding season and remain within the population (probability = sJ_t) contribute to next year's yearling class ($N_{Y,t+1}$). Yearlings and adults that survive to the next breeding season and remain within the population (probability = sA_t) become part of next year's adult age class ($N_{A,t+1}$).

2.1.2 Code implementation including demographic stochasticity

Population process models within IPMs are typically implemented as stochastic models that account for randomness in the outcomes of demographic processes at the individual level ("demographic stochasticity", Caswell, 2001; Kéry and Schaub, 2011). The model described here is no different, meaning that the numbers of breeders, fledglings, and survivors are treated as binomial and Poisson random variables.

Reproduction is modelled via two sets of random variables: a binomial random variable representing the number of breeders in age class a in year t, $B_{a,t}$ and a Poisson random variable representing the number of fledlings produced by breeders of age class a in year t, $Juv_{a,t}$. The implementation in the BUGS language used in the SPI-IPM code (`IPMSetup.R`, lines 231-241) looks like:

```
for (t in 1:Tmax){
  for(a in 1:A){

    ## 1) Breeding decision
    B[a,t] ~ dbin(pB[a,t], N[a,t])

    ## 2) Offspring production
    Juv[a,t] ~ dpois(B[a,t]*CS[a,t]*pNS[t]*sN[a,t]*0.5)
  }
}
```

In the code, the age indeces $a = 1$ and $a = A = 2$ correspond to yearlings and adults, respectively.

Analogous to breeders, the numbers of local survivors – both fledglings surviving their first year and becoming yearlings, and yearlings and adults surviving to the next year – are implemented as binomial random variables (`IPMSetup.R`, lines 243-255):

```
for (t in 1:(Tmax-1)){

  ## 3) Annual survival of local birds
  # Juveniles -> Yearlings
  localN[1,t+1] ~ dbin(sJ[t], sum(Juv[1:A,t]))
  # Yearlings/Adults -> adults
  localN[2,t+1] ~ dbin(sA[t], sum(N[1:A,t]))

  ## 4) Immigration
  for(a in 1:A){
    N[a,t+1] <- localN[a,t+1] + Imm[a,t+1]
  }
}
```

Immigrant numbers are also treated as outcomes of stochastic processes, and these are detailed in 2.2.5 Immigrant count data likelihood.

2.2 Data likelihoods

IPMs obtain information on the population model's parameters (population sizes and vital rates) from several different data sets. Information in each data set is channeled into model parameters via one or multiple data likelihoods. The SPI-IPM contains five data modules consisting of a total of eight data likelihoods: nest count data (one likelihood), clutch size data (two likelihoods), fledgling count data (three likelihoods), mark-recapture data (one likelihood), and immigrant count data (one likelihood). The likelihoods contained in each data module are described in detail in the following sub-chapters. The underlying data sets are introduced in Chapter 1 of the manual.

2.2.1 Nest count data likelihood

The population model defines the true size of the female segment of the breeding population in any year t via the year- and age-specific number of breeding females:

$$B_{a,t} \sim Binomial(N_{a,t}, pB_{a,t}) \quad (2.2)$$

The total size of the female breeding population (the sum of $B_{a,t}$ over all age classes) in year t is expected to correspond closely with the observed number of first clutches laid in any year ($NestCount_t$). The breeding population count thus contains information about both breeding probability ($pB_{a,t}$) and population size ($N_{a,t}$) and its likelihood can be defined as:

$$NestCount_t \sim Poisson((B_{Y,t} + B_{A,t}) \times NS\_Data_t) \tag{2.3}$$

Here, the year-specific variable NS_Data_t is a correction factor introduced for dealing with years in which no nest survey data was collected ($NestCount_t = 0$ due to lack of sampling). NS_Data_t is set to 0 for years without data collection, and to 1 in all other years.

The likelihood is written almost identically in the code, the only difference being the use the `sum()` function over age classes 1 to $A = 2$ when specifying the Poisson distribution (this provides more flexibility for extending the model to more than two age classes):

```
for(t in 1:Tmax){
  NestCount[t] ~ dpois(sum(B[1:A,t])*NS_Data[t])
}
```

2.2.2 Clutch size data likelihoods

The counting of incubated eggs provides information about both individual-level clutch size ($CS_{a,t}$) and reproductive output at the population level. Consequently, two separate likelihoods can be specified for within the clutch size data module.

Each individual clutch size observation can be treated as the outcome of a Poisson process with an expected value of $CS_{a,t}$ (where a is the age of the female that laid the clutch, and t the year in which the clutch was laid). In the code, the likelihood is formulated for each clutch size observation x (out of a total of `CS_X` observations) and includes nested indexing of the expectation using data on female age (`CS_FAge`) and year (`CS_year`):

```
for(x in 1:CS_X){
    ClutchSize[x] ~ dpois(CS[CS_FAge[x], CS_year[x]])
}
```

Since both year and female age need to be part of the provided data, the individual-level clutch size likelihood can only be used with complete observations, i.e. clutches for which both number of eggs and age of the mother are known (year should always be known).

For the population-level likelihood, on the other hand, data on clutch sizes can be included irrespective of whether the age of the mother is known. The total number of eggs counted in all nests laid in year t can be described as:

$$EggNoTot_t \sim Poisson(sum(B_{Y:A,t} \times CS_{Y:A,t}) \times p_t^{EggNo}) \qquad (2.4)$$

Since the product $B_{a,t} \times CS_{a,t}$ corresponds to all eggs laid in all nests/nestboxes within the study site, another correction factor (p_t^{EggNo}) is needed to account for the fact that eggs may not have been counted in all surveyed nests with breeding activity in each year. p_t^{EggNo} (`EggNoSP[t]` in code) thus contains information of the year-specific proportionof surveyed nests for which the numbers of eggs were counted.

In code, the likelihood for the number of eggs at the population is written as:

```
for(t in 1:Tmax){

    # Expected "true" egg number (by mother age class)
    EggNo.ex[1:A,t] <- B[1:A,t]*CS[1:A,t]

    # Observed egg number (corrected by data availaility)
    EggNoTot[t] ~ dpois(sum(EggNo.ex[1:A,t])*EggNoSP[t])

}
```

2.2.3 Fledgling count data likelihoods

Analogous to observations of clutch size, counts of fledglings contain information on reproduction at both individual and population level. Distributions of number of fledglings produced from a clutch are often 0-inflated because incidents of harsh weather, predation, adandonment, etc. may result in complete loss of entire clutches. To account for this, we split data on fledgling numbers and formulated separate likelihoods for the survival of the clutch as a whole (probability of a nest not failing completely, pNS_t) and for each chick subsequently surviving to fledgling (probability $sN_{a,t}$).
Whether or not a clutch succeeded (i.e. at least one chick survived to fledging, `anyFledged`) was coded using 1 (success) and 0 (failure) and modelled as the outcome of a Bernoulli process with a year-dependent success probability pNS_t:

```
for(x in 1:F_X){
  anyFledged[x] ~ dbern(pNS[F_year[x]])
}
```

For the subset of successful clutches, the number of fledglings produced (`NoFledged`) was modelled as a binomial random variable with each egg laid

in the clutch (`NoLaid`) having a probability of $sN_{a,t}$ (where a = age of the mother) to survive and fledge:

```
for(x in 1:NoF_X){
    NoFledged[x] ~ dbin(sN[NoF_FAge[x], NoF_year[x]], NoLaid[x])
}
```

At the population level, both processes (nest success and survival to fledging conditional on nest success) were combined into a single Poisson likelihood describing the total number of fledglings counted in the population in a given year t as the product of the number of breeding females, clutch size, nest success, and survival to fledging:

$$FledgedTot_t \sim Poisson(sum(B_{Y:A,t} \times CS_{Y:A,t} \times pNS_t \times sN_{Y:A,t}) \times p_t^{Fledged}) \quad (2.5)$$

As in the clutch size data module (Chapter 2.2.2), a correction factor quantifying the proportion of nests for which data is availeble ($p_t^{Fledged}$, `FledgedSP[t]` in code) is used to account for missing records of fledgling numbers.

In the code, calculation of the expected number of fledglings is based on the expected number of eggs as calculated in the clutch size data module (Chapter 2.2.2):

```
for(t in 1:Tmax){

    # Expected "true" fledgling number (by mother age class)
    Fledged.ex[1:A,t] <- EggNo.ex[1:A,t]*pNS[t]*sN[1:A,t]

    # Observed egg number (corrected by data availaility)
    FledgedTot[t] ~ dpois(sum(Fledged.ex[1:A,t])*FledgedSP[t])
}
```

2.2.4 Mark-recapture data likelihood

In the SPI-IPM, capture histories of marked birds are analysed using an age-specific Cormack-Jolly-Seber (CJS) model (Cormack, 1964; Jolly, 1965; Seber, 1965), which allows estimation of parameters associated with both annual survival and the (re-)capturing process.
The survival parameters sJ_t and sA_t describe the probability of surviving from one breeding season to the next for fledglings/juveniles and adult females, respectively. Since field-based sexing fledglings is not possible in the hatch year, first-year survival in the CJS likelihood was set to $0.5sJ_t$, representing the probability that a fledgling is female and survives (recaptures of adult males were

removed from the capture histories).
The time- and age-dependent recapture parameters $p^{Recap}_{Age,t}$ describe the probability that an age class a individual is recaptured and identified during the breeding season of year t given that it survived the time interval $t-1$ to t. For many of the bird populations included in SPI-Birds, (re-)captures of birds are carried out at the nestboxes during the breeding season only. Consequently, two conditions need to be met for a (re-)capture besides the individual being alive: 1) the individual breeds in a nestbox (as non-breeders and birds breeding in natural cavities are not captured) and 2) the individual is actually captured and marked/identified while breeding in a nestbox. The recapture probability is therefore the product of the probability of breeding in a nestbox ($pB_{Age,t}$) and the probability of capture and identification given breeding in a nestbox (p^{CapB}_t):

$$p^{Recap}_{Age,t} = pB_{Age,t} \times p^{CapB}_t \tag{2.6}$$

The parameters $pB_{Age,t}$ and p^{CapB}_t are confounded, and auxiliary data on one of them is required to separately estimate them. Here, raw data on the annual proportions of surveyed nests for which the identity of the breeding female was recorded (as a result of ringing or recapture) is used to approximated p^{CapB}_t, allowing the CJS model to estimate age- and time-specific breeding probabilities ($pB_{Age,t}$).
CJS models in Bayesian frameworks have traditionally been implemented either as latent states models with Bernouilli likelihoods or as multinomial likelihoods for data re-formatted as "m-arrays" (Gimenez et al., 2007; Kéry and Schaub, 2011). The former typically results in long runtimes and high computational costs, while the improved efficiency of the latter is tied to an implementation that suffers from being neither particularly intuitive nor user-friendly. To avoid these pitfalls, the CJS model in the SPI-IPM is implemented using a much more efficient marginalized likelihood (that integrates over all latent states) that can be applied to unique (not individual) capture histories only (following Turek et al., 2016):

```
## Likelihood with custom distribution
for (i in 1:n.CH){

  y.sum[i, first.sum[i]:last.sum[i]] ~ dCJS_vv_sum(
      probSurvive = phi.CH[i, first.sum[i]:last.sum[i]],
      probCapture = p.CH[i, first.sum[i]:last.sum[i]],
      len = last.sum[i]-first.sum[i]+1,
      mult = CHs.count[i])
}
```

In practice, this involves the use of a custom distribution `dCJS_vv_sum`, and more information on the likelihood and its implementation using NIMBLE is provided in Chapter 4.1 (Efficient implementation using NIMBLE).

2.2.5 Immigrant count data likelihood

In many nestbox studies, chick ringing is an exhaustive effort and it is not unreasonable to assume that all chicks hatched in nestboxes within a study population are ringed before fledging. Similarly, assuming that females captured in nestboxes are breeding (or attempting to breed) is often not far from the truth. If those two assumptions are largely met, then the annual numbers of newly ringed adult females ($ImmNoObs_t$) can provide information about immigration or – more specifically – about the latent number of newly immigrated females breeding in nestboxes each year ($ImmNoTot_t$). Typically, age will be unknown for immigrant females (unless they were marked as chicks elsewhere) and hence there is no age index on $ImmNoObs_t$ and $ImmNoTot_t$. In `SPI-IPM`, we use a Poisson observation model to describe the relationship between observed and true numbers of breeding immigrant females:

$$ImmNoObs_t \sim Poisson(ImmNoTot_t \times p_t^{ImmDetect}) \quad (2.7)$$

The expected value represents the true number of female breeding immigrants in year t ($ImmNoTot_t$) corrected by an annual probability of detecting (= ringing) such individuals ($p_t^{ImmDetect}$). In most nestbox studies, ringing and recapturing of breeding birds are conducted in tandem as part of the same protocol, and we can consequently approximate adult ringing probability using recapture probability (as used in the likelihood for capture histories): $p_t^{ImmDetect} = p_t^{CapB}$). In a few exceptional years in some datasets, however, adult birds may have been ringed, but no recaptures recorded (i.e. $p_t^{CapB} = 0$ but $p_t^{ImmDetect} > 0$). These cases call for estimation of (or, in other words, accounting for the uncertainty in) the corresponding $p_t^{ImmDetect} > 0$. As long as such cases are relatively few, this can be accommodated by specifying a non-informative prior for the unknown value[1]:

```
## Immigrant detection (= marking) probability
for(t in 1:Tmax){
  PropImmDetect[t] ~ dunif(0, 1)
}
```

The latent number of breeding immigrants, informed by data as outlined above, is estimated as pooled across age classes ($ImmNoTot_t$), but the since the population model in `SPI-IPM` is age structured $ImmNoTot_t = ImmB_{Y,t} + ImmB_{A,t}$, where $ImmB_{Y,t}$ and $ImmB_{A,t}$ the numbers of breeding yearling and adult immigrants, respectively. These quantities are linked to the total numbers of immigrants in each age class (breeding and non-breeding), $Imm_{a,t}$, that appear

[1]For estimating only a subset of values within a vector/array (= partially observed variables), data (here `PropImmDetect[t]`) is provided including `NA` for unknown values and initial values are provided for the corresponding indeces (with initial values matching indeces of known data points being set to `NA`). Handling partially observed variables is discussed some more under 3.2.3 Imputation of missing covariate values

in the age-structured population model (Chapter 2.1 through age-specific breeding probabilities. By assuming equal breeding probabilities for locally recruited and newly immigrated females, we can use the breeding probabilities estimated via the mark-recapture likelihood ($pB_{a,t}$) to make the connection:

$$ImmB_{a,t} \sim Binomial(Imm_{a,t}, pB_{a,t}) \tag{2.8}$$

In the code, the sub-model for immigration contains both the likelihood for the immigrant count data pooled across ages and the relationship between breeding immigrant females and all immigrant females in both age classes:

```
## Likelihood for the number/age distribution of immigrant females
for (t in 2:Tmax){

  # Latent true number of breeding immigrants (count observation model)
  ImmNoObs[t] ~ dpois(sum(ImmB[1:A,t])*PropImmDetect[t])

  # Number of breeding immigrants per age class
  for(a in 1:A){
    ImmB[a,t] ~ dbin(pB[a,t], Imm[a,t])
  }
}

ImmNoObs[1] <- 0
ImmB[1:A,1] <- 0
```

Note that various immigrant numbers are set to 0 at $t = 1$ since there is no way of distinguishing between locally-born and newly immigrated birds in the first year of study.

2.3 Priors and constraints

In the model description above, all vital rate parameters, as well as initial population size and immigrant numbers, appear as fully time- and age-dependent parameters (i.e. indexed by $t =$ year and $a =$ age class). `SPI-IPM` is a hierarchical model, meaning it also describes how demographic quantities vary across time and age classes and requires priors for the parameters defining these underlying processes. The priors in the model's standard implementation are all non- or only weakly informative, but they can easily be replaced with more informative priors if required (see e.g. Chapter 8.2).

Time- and age-dependence in all vital rates is expressed using generalized mixed-effect models. The exact model structure for each vital rate, including link function, is described in detail in Chapter 3. Irrespective of the vital rate, the basic

model parameters fall into three categories: (age-specific) intercepts representing time-average vital rates (μ), slope parameters for the effects of temporal covariates (β), and standard deviations describing the distribution of random year effects (σ). The intercept parameters μ are defined on the natural scale to allow for more intuitive setting of priors. As such, non-informative Uniform(0,1) priors can be used for the μ of all vital rates representing probabilities (μ_a^{pB}, μ^{pNS}, μ_a^{sN}, μ^{sJ}, and μ^{sA}). For the age-specific average clutch size (μ_a^{CS}), we use a Uniform(0,10) prior. This latter prior is not completely uninformative, as it sets an upper limit of 10 eggs for the average clutch size (this is reasonable for pied flycatchers *Ficedula hypoleuca* for which `SPI-IPM` was initially developed, but may have to be adjusted for species that lay larger clutches). All slope parameters β are given Uniform(-5,5) priors, and all σ parameters (except for the one linked to immigration, σ_a^{Imm}) are given Uniform(0,10) priors.

Priors and constraints are also required for the initial population size, i.e. $N_{Y,1}$ and $N_{A,1}$ since the population process model only begins predicting N from the second year ($t = 2$) onward. Immigrant numbers have their own set of priors (see below), and since $N_{a,t} = localN_{a,t} + Imm_{a,t}$, priors have to be set for $localN_{a,1}$ specifically. `SPI-IPM` uses what is essentially a discrete uniform prior for initial local population size:

```
## Initial population sizes

for(a in 1:A){
  localN[a,1] ~ dcat(DU.prior[1:N1.limit])
  N[a,1] <- localN[a,1] + Imm[a,1]
}

DU.prior[1:N1.limit] <- 1/N1.limit
```

Here, `N1.limit` is a user-specified maximum possible number of local females in any age class at the first time-step. The discrete uniform distribution is approximated using a categorical distribution in which each integer number between 1 and `N1.limit` has the same probability (`1/N1.limit`) of occurring. This approach is equivalent to using a regular Uniform(0,`N1.limit`) distribution and subsequently rounding it.

The final set of priors and constraints in the model are for immigrant numbers $Imm_{Y,t}$ and $Imm_{A,t}$. At the first time-step ($t = 1$), immigrant numbers are set to 0 (since there is no way of distinguishing previously local and newly immigrated individuals in the first year of a study). Numbers at any subsequent time-step are assumed to follow a 0-truncated normal distribution with age-specific mean $AvgImm_a$ (`AvgImm[a]`) and standard deviation σ_a^{Imm} (`sigma.Imm[a]`)):

```
## Latent number of all immigrants (breeding & non-breeding) per age class
Imm[1:A,1] <- 0
```

```
for(t in 2:Tmax){
  for(a in 1:A){

    ImmCont[a,t] ~ T(dnorm(AvgImm[a], sd = sigma.Imm[a]), 0, Inf)
    Imm[a,t] <- round(ImmCont[a,t])
  }
}
```

The rounding function ensures that immigrant numbers are integers. Priors are then required for $AvgImm_a$ and σ_a^{Imm}, and these are given as uniform distributions with values ranging from 0 to (a multiple of) a user-specified maximum possible number of immigrant females in any age class:

```
for(a in 1:A){
  AvgImm[a] ~ dunif(0, AvgImm.limit)
  sigma.Imm[a] ~ dunif(0, AvgImm.limit*10)
}
```

Chapter 3

Modelling temporal variation

Vital rates are expected to vary among years due to changes in environmental conditions. `SPI-IPM` is set up to account for among-year variation in different (age-specific) vital rates $X_{a,t}$ using fixed effects of supplied environmental covariates ($cov1_t$, $cov2_t$,...) and year random effects (ϵ_t^X). The resulting generalized linear mixed-models for time- and age-specific vital rates therefore take the following form:

$$link(X_{a,t}) = link(\mu_a^X) + \beta_{cov1}^X \times cov1_t + \beta_{cov2}^X \times cov2_t + ... + \epsilon_t^X \tag{3.1}$$

Here, μ_a^X is the age-specific average vital rate (intercept) and β_{cov1} and β_{cov2} are the slopes for the effects of covariates $cov1$ and $cov2$ on the link scale, respectively. The link function depends on the vital rate, and is set to logit for breeding probabilities ($pB_{a,t}$), nest success probabilities (pNS_t), and survival probabilities ($sN_{a,t}$, sJ_t, sA_t) and log for clutch size ($CS_{a,t}$).

3.1 Random year variation

The random year effects included in the basic implementation of `SPI-IPM` are assumed to be normally distributed such that

$$\epsilon_t^X \sim Normal(0, \sigma^X) \tag{3.2}$$

where σ^X is the standard deviation of random year effects on vital rate $X_{a,t}$. The random effects in the basic implementation are also age-independent, meaning that ϵ_t^X is included in the equations for both the yearling vital rate $X_{Y,t}$ and the adults vital rate $X_{A,t}$. The exception are the annual survival rates for juveniles

(sJ_t) and (sA_t), which both have separate random effects because the drivers of survival variation are expected to vary between those two age classes, and the basic implementation includes an additional covariate effect sJ_t only[1]:

```
for(t in 1:Tmax){

  ## Age- and time-dependent survival probabilities
  logit(sJ[t]) <- logit(Mu.sJ) + beta3.sJ*cov3[t] + epsilon.sJ[t]
  logit(sA[t]) <- logit(Mu.sA) + epsilon.sA[t]

  ## Temporal random effects
    epsilon.sJ[t] ~ dnorm(0, sd = sigma.sJ)
    epsilon.sA[t] ~ dnorm(0, sd = sigma.sA)
}
```

All random effects are treated as independent (= not correlated) in the basic implementation of the model, but the inclusion of correlation of random effects across age-classes and/or vital rates is straightforward to implement[2] using e.g. multivariate normal distributions or approaches similar to the one used in Nater et al. (2020).

3.2 Temporal covariates

The basic implementation of `SPI-IPM` features an example covariate model structure that was motivated by an analysis of populations of pied flycatcher ((Ficedula hypoleuca)) breeding in the UK. It involves three different covariates ($cov1$, $cov2$, and $cov3$) that are assumed to affect nest success probability (pNS_t), nestling survival ($sN_{a,t}$), and juvenile survival (sJ_t). However, it is very straightforward to alter the code to fit whatever alternative covariate structure is suitable for your particular analysis since the inclusion different/additional continuous and categorical covariates always works according to the same principles (see also Kéry and Schaub, 2011). All covariates are need to be passed to `SPI-IPM` as vectors or arrays.

[1]Note that the implementation of this in `IPMSetup.R` looks slightly different as it uses a vectorized formulation (calculations done over all time-steps simultaneously instead of using a for-loop). Vectorized calculations are a nifty feature available in NIMBLE (but not BUGS and JAGS); more on this in Chapter 4.1.

[2]Wheter or not formally including random effects correlations is useful or not depends on the biological questions of interest and the amoung of data available. When testing a model with correlated random effects for juvenile and adult survival on seven datasets from breeding populations of pied flycatchers in the UK, I found that estimates did not differ from those obtained from a model with independent random effects, and the posterior distribution for the correlation coefficient was so wide that no inference on strength or direction of the correlation was possible.

3.2.1 Continuous variables

Continuous variables are included as covariate effects using a specific slope parameter (β). Temporal effects then take the form $\beta_{cov1} \times cov1_t$, where β_{cov1} is an estimated parameter quantifying strength and direction of the effect and $cov1_t$ is the value of covariate $cov1$ at time t. In (generalized) linear mixed effects models as they are used in `SPI-IPM`, effects of several different covariates can just be added up on the link scale. For pNS_t, for example, this codes as[3]:

```
for(t in 1:Tmax){
  logit(pNS[t]) <- logit(Mu.pNS) + beta1.pNS*cov1[t] + beta2.pNS*cov2[t] + epsilon.pNS[t]
}
```

The basic implementation of `SPI-IPM` includes the following covariate models:

$$logit(pNS_t) = logit(\mu^{pNS}) + \beta^{pNS}_{cov1} \times cov1_t + \beta^{pNS}_{cov2} \times cov2_t + \epsilon^{pNS}_t$$
$$logit(sN_{a,t}) = logit(\mu^{pNS}_a) + \beta^{sN}_{cov1} \times cov1_t + \beta^{sN}_{cov2} \times cov2_t + \epsilon^{sN}_t$$
$$logit(sJ_t) = logit(\mu^{sJ}) + \beta^{sJ}_{cov3} \times cov3_t + \epsilon^{sJ}_t$$

All covariates are continuous annual variables that have been standardized and centered (mean = 0, sd = 1) prior to analysis. $cov1$ and $cov2$ represent environmental conditions during the incubation and nestling period and hence influence nest success (pNS_t) and nestling survival ($sN_{a,t}$). In the case of the latter, covariates are further assumed to have the same magnitude of effect on the nests of yearling and adult females (i.e. the β parameters are independent of age). $cov3$, on the other hand, symbolizes environmental conditions after fledging which impact juvenile annual survival (sJ_t). No covariate effects are included for the other vital rates.

3.2.2 Categorical variables

The basic `SPI-IPM` does not include any categorical covariates, but since such covariates may be relevant to a wide range of questions (e.g. some of the points raised in Chapter 8.2), I briefly illustrate how they could be included into vital rate models.

Generally, there are two approaches to modelling categorical covariates in this context.

The first approach works analogous to the approach for continuous covariates, i.e. it uses the form $\beta_{cov} \times cov_t$. This is most relevant for binary categorical covariates that symbolize some sort of "on-off" process. An example of this would be if you would like to model the effect of an experimental treatment that has been performed in some years ($cov_t = 1$) but not others ($cov_t = 0$).

---
[3] see Footnote 1

Your binary covariate then works as a "switch" that determines whether or not the effect of the experimental treatment (β_{cov}) applied in a given year t or not since $link(X_t) = link(\mu^X) + \beta_{cov_t} \times cov_t$ becomes $link(\mu^X) + \beta_{cov_t}$ when $cov_t = 1$ and $link(\mu^X)$ when $cov_t = 0$.

The second approach works via (nested) indexing and is more flexible since it can technically account for any number of levels in your categorical covariate. This can be relevant, for example, for categories of years ("good", "average", "bad"), habitat types ("deciduous forest", "coniferous forest"), or individuals ("male", "female"). The approach still uses β parameters, but instead of multiplying the β with the covariate value, we index the β by the covariate value such that β_1 corresponds to the effect of category 1 ($cat = 1$), β_2 corresponds to the effect of category 2 ($cat = 2$), and so on:

$$link(X_{cat}) = link(\mu^X) + \beta_{cat} \tag{3.3}$$

Priors then need to be provided for each category-specific β.

In practice, `SPI-IPM` still requires vital rates X to be indexed by age class and year (at least with the population model described in Chapter 2). That's where nested indexing becomes relevant. The relationship of a vital rate $X_{a,t}$ with a categorical year covariate can, for example, be coded as follows:

```
for(a in 1:A){
  for(t in 1:Tmax){
      log(X[a,t]) <- log(Mu.X[a]) + beta[cov[t]]
  }
  Mu.X ~ dunif(0, 10)
  beta ~ dunif(-5, 5)
}
```

where `cov[t]` is a vector of integer numbers that represent the different year categories.

Introducing categorical effects that rely on additional structure beyond year and age (for example effects of sex or location) requires changing the underlying population model. Such extensions are currently not implemented in `SPI-IPM`, but see Chapter 8 for some perspectives.

3.2.3 Imputation of missing covariate values

Perhaps you have been wondering about how to deal with NAs in your covariate data? The good news is that `SPI-IPM` (just like any other Bayesian hierarchical model) can accommodate NAs in both continuous and categorical covariates. The (perhaps) less good news is that how well it works really depends on how large a proportion of your covariate data is NA.

There are three practical requirements for working with partially observed covariate data:

1. Your covariate data containing numbers for your observed covariate values and NAs for your unobserved/unknown covariate values
2. Initial values with the same dimensions as your covariate data containing numbers in the positions of NA covariate values and NAs in the positions of observed covariate values.
3. A model describing the distribution of missing covariate values.

Numbers 1. and 2. are pretty self-explanatory (but see Chapter 4.2 for more information on sampling initial values). Number 3. is going to depend on what type of covariate data you are dealing with.

The basic implementation of `SPI-IPM` is set up to be able to deal with NA values in the continuous temporal covariates $cov1$, $cov2$, and $cov3$. These covariates are assumed to have been standardized and centered, i.e. they should more or less follow a $Normal(mean = 0, sd = 1)$ distribution. If we assume that the observed and unobserved covariate values follow the same distribution (i.e. the missing values are a random subset of all values), this can be used to specify the process model for the missing covariates in the code:

```
for(t in 1:Tmax){
  cov1[t] ~ dnorm(0, sd = 1)
  cov2[t] ~ dnorm(0, sd = 1)
  cov3[t] ~ dnorm(0, sd = 1)
}
```

There are numerous alternatives for specifying distributions of missing values in continous covariates, and they can be accommodated by changing the above section in the code.

The number of candidate distributions are a bit more limited when there are missing values in categorical covariates. Chapter 8.2.1 outlines an example for dealing with partially missing information on individual age. It may also be helpful to remember that models with partially observed categorical variables are essentially “mixture models” including auxiliary data about the underlying distribution.

3.3 Notes on covariate selection

To be added later.

Chapter 4

IPM Implementation

4.1 Efficient implementation using NIMBLE

`SPI-IPM` is implemented in a Bayesian framework and, more specifically, it is fit using NIMBLE (de Valpine et al., 2017). In many ways, NIMBLE is the successor to BUGS and JAGS, using very similar syntax but offering higher efficiency, much more flexibility, and a range of smaller "quality-of-life improvements". Unlike its predecessors, NIMBLE is not a software on its own and is installed via the R-package `nimble` (de Valpine et al., 2021). A lot of resources on setting up and working with NIMBLE are available on the NIMBLE website and this manual will not give a comprehensive overview of NIMBLE or the `nimble` R package. In the following, I will instead briefly outline some of NIMBLE's features that `SPI-IPM` makes use of, and that may be good to be aware of particularly for people switching over from BUGS/JAGS. I will also briefly outline the structure for the basic call to NIMBLE to run a model.

4.1.1 Alternative specification of distributions

First, NIMBLE allows alternative specifications for statistical distributions (see the NIMBLE manual for an overview). This is particularly convenient for some commonly used distributions such as the normal distribution, which had to be specified via a mean and a precision (τ) in BUGS/JAGS. The latter was then often related to the (some might say) more useful standard deviation (σ):

```
for(t in 1:Tmax){
  epsilon[t] ~ dnorm(0, tau)
}
tau <- pow(sigma, -2)
sigma ~ dunif(0, 5)
```

Looks familiar?
NIMBLE allows to specify the distribution parameters exactly as above, but also offers alternatives such as parameterisation by mean and standard deviation directly, e.g.:

```
for(t in 1:Tmax){
  epsilon[t] ~ dnorm(0, sd = sigma)
}
sigma ~ dunif(0, 5)
```

4.1.2 Vectorized calculations

A second useful feature is vectorized calculation of deterministic nodes. At some point, everyone has heard that when programming in `R`, vectorization outperforms for-loops when it comes to efficiency and speed. In many cases, this is also true for MCMC, and NIMBLE therefore supports vectorization of calculations for deterministic nodes (i.e. nodes assigned via `<-` in the code). `SPI-IPM` uses vectorized calculations instead of for-loops in a variety of cases, for example for defining the models underlying temporal variation in vital rates:

```
logit(pNS[1:Tmax]) <- logit(Mu.pNS) + beta1.pNS*cov1[1:Tmax] + beta2.pNS*cov2[1:Tmax]
```

instead of

```
for(t in 1:Tmax){
  logit(pNS[t]) <- logit(Mu.pNS) + beta1.pNS*cov1[t] + beta2.pNS*cov2[t] + epsilon.pNS
}
```

It is important to note that vectorization works for deterministic nodes, but – per today – not for stochastic nodes (i.e. nodes assigned via `~`).

4.1.3 Custom distributions

The third class of NIMBLE features that `SPI-IPM` capitalizes on is the ability to define custom distributions. Specifically, `SPI-IPM` uses a custom distribution in the likelihood for the mark-recapture data.
In most IPMs, the analysis of mark-recapture data to estimate survival represents the bottleneck for MCMC efficiency: sampling hundreds – if not thousands – of latent alive or dead states is computationally expensive and results in long MCMC runtimes (Gimenez et al., 2007). Summarising individual capture histories into "m-arrays" has long been the only way to reduce runtimes of Bayesian mark-recapture models (Kéry and Schaub, 2011), but many find this format less intuitive and it quickly becomes convoluted and impractical when working with

predictors/groups other than age class. With the rise of NIMBLE, Turek et al. (2016) developed an approach to defining marginalized likelihoods that integrate over latent states and lead to tremendous increases in MCMC efficiency. Basic implementations of the marginalized likelihood for mark-recapture models were included in the `nimbleEcology` R package (Goldstein et al., 2021).
`SPI-IPM` uses an extended version of the `dCJS_vv` distribution contained in `nimbleEcology` which not only integrates over latent states, but also runs on only unique – instead of all – capture histories (analogous to the goose example in Turek et al., 2016). Preliminary tests have shown that mark-recapture models with a likelihood specified using this distribution (`dCJS_vv_sum`) have very similar runtimes as implementations using m-arrays for small to medium-sized datasets, and outspeed m-array formulations in for larger datasets.
The specification of the custom distribution is contained in `dCJS_CustomDist.R` and its implementation within the model code looks quite minimalistic:

```
## Likelihood with custom distribution
for (i in 1:n.CH){
  y.sum[i, first.sum[i]:last.sum[i]] ~ dCJS_vv_sum(
       probSurvive = phi.CH[i, first.sum[i]:last.sum[i]],
       probCapture = p.CH[i, first.sum[i]:last.sum[i]],
       len = last.sum[i]-first.sum[i]+1,
       mult = CHs.count[i])
}
```

`y.sum` is a matrix containing all `n.CH` unique capture histories and the likelihood is fit for the range of observations between the first capture of an individual (`first.sum`) and the last possible time this individual could still have been alive[1](`last.sum`). `probSurvive` and `probCapture` are vectors containing the survival and recapture probabilities relevant for the capture history (see `IPMSetup.R` and Chapter 2.2.4 for details). The argument `len` quantifies the length of `y.sum` and is required for successful compilation of the custom function. Finally, `mult` is an integer number specifying how many individual birds shared capture history `i`.

4.1.4 Calling NIMBLE from R

The simplest way to implement and run a Bayesian model in NIMBLE is via the wrapper-function `nimbleMCMC`. The structure of the `nimbleMCMC` call is very similar to the functions provided with the different R-packages for running BUGS/JAGS and – for `SPI-IPM` – looks like this (code line 49 in `IPMRun_PopID.R`):

[1] in practice this can be set to e.g. year of the last recorded observation of the individual plus maximum lifespan, plus some. In my analysis of UK-breeding pied flycatchers, for example, I have set it – generously – to 20 years after the last recorded capture.

```
SPI.IPM <- nimbleMCMC(code = SPI.IPMcode, constants = SPI.IPMconstants, data = SPI.IPMc
```

`code` is the model code, formatted as `nimbleCode`. `constants` and `data` contain all information relevant for parameterising the model, as well as all the observational data (see Chapter 1.4). `inits` is a list of initial values that is described further below, and `monitors` is a character vector containing the names of all paramters that should be monitored. `niter`, `nburnin`, `nchains`, and `thin` are the numbers of iterations, samples to discard as burn-in, number of chains, and thinning interval for the MCMC. `setSeed` is absolutely crucial! By setting the seed within the call to NIMBLE, your entire MCMC becomes reproducible which is invaluable also for trouble-shooting. The final argument, `samplesAsCodaMCMC`, is for people like me who like to get back raw samples and make their own summaries instead of having the function return larger objects containing the samples along with a variety of summaries and other stats.

4.2 Simulation of initial values

As a general rule, all parameters that have priors provided for them within Bayesian models also require initial values. For `SPI-IPM`, this applies to:

- Vital rate averages (μ /`Mu`)
- Environmental effects on vital rates (β /`beta`)
- Standard deviation for year random effects on vital rates (σ /`sigma`)
- Initial population sizes (`localN[,1]`)
- Average and standard deviation of immigrant numbers (`AvgImm`, `sigma.Imm`)
- Missing covariate values (subset of `cov1`, `cov2`, `cov3`)
- Missing observation probabilities (subset of `PropImmDetect`)

Provided that initial values for all of these parameters have been provided, NIMBLE is able to calculate initial values for all of the remaining downstream nodes using the realtionships specified in the model code. In practice, however, the more different data sources and sub-models are included in an integrated model, the more likely it is that this "automatic" initialization of nodes will result in conflicts, i.e. initial values that are not compatible with each other.

While it may work for a single IPM to re-sample initial values repeatedly until a set without conflicts has been found by chance, this is vastly impractical for a framework like `SPI-IPM` which is designed to also work well for comparative analyses, i.e. may be run on several datasets/populations at a time.
For that reason, manual initialization of all nodes makes sense for `SPI-IPM` and is implemented in the function `SPI_IPM.inits` (in file `InitSim.R`).

The function runs a simulation of the entire population model, including the reconstruction of missing covariate and detection parameter values in four steps:

1. Simulation of missing covariate values
2. Simulation of age- and time-specific vital rates
3. Simulation of initial population size and population trajectory over the study period, incl. immigration
4. Simulation of missing detection parameters

The elements passed to the function are the collated data and constants (`IPM.data` and `IPM.constants`, see Chapter 1.4), as well as a logical argument determining whether initial values for year random effects should be sampled from their simulated distribution or set to 0 (`sampleRE`).

To sample initial values for several chains, the function is called multiple times within a list.

```
Inits <- list(
  SPI_IPM.inits(IPM.data = SPI.IPMdata, IPM.constants = SPI.IPMconstants, sampleRE = FALSE),
  SPI_IPM.inits(IPM.data = SPI.IPMdata, IPM.constants = SPI.IPMconstants, sampleRE = FALSE))
```

is an example for initializing two chains.

Note that I am sampling the initial values and storing them in an object called `Inits` in the example, which I then pass to `nimbleMCMC` afterwards, instead of calling the `SPI_IPM.inits` directly within the call to `nimbleMCMC`. The reason for "pre-sampling" initial values in this way is that it makes trouble-shooting much easier: if initialization problems occur at specific nodes, I can investigate both the associated data AND pre-sampled initial values to work out the cause of the discrepancy.
For the purpose of facilitating trouble-shooting, but also to promote reproducibility, it is also good practice to set a seed prior to pre-sampling initial values (in `IPMRun_PopID.R`, for example, the seed is set right at the start of the script).

4.3 Test runs and full runs: chains, iterations, burn-in, and thinning

If you have ample experience working with Bayesian models and MCMC, you can jump over this section.

The following is a (perhaps obvious) practical tip for users that are newer to Bayesian modelling and/or IPMs: always do a test-run of your implementation to make sure it works before running a long MCMC!

In the original code, `SPI-IPM` is set up for a full run of 4 chains with 200 000 iterations each (see code lines 26-29 in `IPMRun_PopID.R`). The first 50 000 iterations of each chain are discarded as burn-in and the remainder thinned by

30, resulting in a combined posterior consisting of $4\times$ 5000 = 20 000 samples. This is rather generous, and it is not unlikely that your analysis will require substantially less iterations to reach convergence (see Chapter 5.1 for how to assess convergence). Nonetheless, running the full MCMC can easily take several hours and ideally, you would discover issues such as bad initialization or mistakes made during code adjustments **before** having to wait that long.
That's where short test runs become useful. The shortest MCMC you can run is two iterations long, no burn-in, and with thinning interval 1 (= no thinning). By running that first, you can check that your model builds smoothly and contains no unwanted NAs, and that initialization works as it should creates no conflicts. With slightly more iterations in a test chain, you can also make sure that all nodes that should be updating are in fact updating. Most importantly, this will allow you to test your model's setup and trouble-shoot some basic implementation issues without having to wait for hours.

4.4 Trouble-shooting implementation issues

Specific content will be added later. In the meantime, a lot of helpful information can be found in the materials on the NIMBLE website and via the Google group "nimble-users".

For issues that are related specifically to `SPI-IPM`, you can also get in touch via email.

Bibliography

Caswell, H. (2001). *Matrix population models: construction, analysis, and interpretation*. Sunderland, Mass.: Sinauer Associates.

Cormack, R. M. (1964). Estimates of survival from the sighting of marked animals. *Biometrika*, 51(3/4):429–438.

de Valpine, P., Paciorek, C., Turek, D., Michaud, N., Anderson-Bergman, C., Obermeyer, F., Wehrhahn Cortes, C., Rodrìguez, A., Temple Lang, D., and Paganin, S. (2021). *NIMBLE: MCMC, Particle Filtering, and Programmable Hierarchical Modeling*. R package version 0.12.1.

de Valpine, P., Turek, D., Paciorek, C. J., Anderson-Bergman, C., Lang, D. T., and Bodik, R. (2017). Programming with models: writing statistical algorithms for general model structures with nimble. *Journal of Computational and Graphical Statistics*, 26(2):403–413.

Gimenez, O., Rossi, V., Choquet, R., Dehais, C., Doris, B., Varella, H., Vila, J.-P., and Pradel, R. (2007). State-space modelling of data on marked individuals. *Ecological Modelling*, 206(3-4):431–438.

Goldstein, B. R., Turek, D., Ponisio, L., and de Valpine, P. (2021). nimbleEcology: Distributions for ecological models in nimble. R package version 0.4.1.

Jolly, G. M. (1965). Explicit estimates from capture-recapture data with both death and immigration-stochastic model. *Biometrika*, 52(1/2):225–247.

Kéry, M. and Schaub, M. (2011). *Bayesian population analysis using WinBUGS: a hierarchical perspective*. Academic Press.

Nater, C. R., Vindenes, Y., Aass, P., Cole, D., Langangen, Ø., Moe, S. J., Rustadbakken, A., Turek, D., Vøllestad, L. A., and Ergon, T. (2020). Size- and stage-dependence in cause-specific mortality of migratory brown trout. *Journal of Animal Ecology*, 89(9):2122–2133.

Plard, F., Fay, R., Kéry, M., Cohas, A., and Schaub, M. (2019). Integrated population models: powerful methods to embed individual processes in population dynamics models. *Ecology*, page e02715.

Seber, G. A. (1965). A note on the multiple-recapture census. *Biometrika*, 52(1/2):249–259.

Turek, D., de Valpine, P., and Paciorek, C. J. (2016). Efficient markov chain monte carlo sampling for hierarchical hidden markov models. *Environmental and ecological statistics*, 23(4):549–564.
